# Synthesis and Biological Evaluation of Disubstituted Pyrimidines as Selective 5-HT_2C_ Agonists

**DOI:** 10.3390/molecules24183234

**Published:** 2019-09-05

**Authors:** Juhyeon Kim, Yoon Jung Kim, Ashwini M. Londhe, Ae Nim Pae, Hyunah Choo, Hak Joong Kim, Sun-Joon Min

**Affiliations:** 1 Center for Neuro-Medicine, Korea Institute of Science and Technology (KIST), Seoul 02792, Korea; 2 Department of Chemistry, Korea University, Seoul 02841, Korea; 3 Department of Applied Chemistry, Hanyang University, Ansan, Gyeonggi-do 15588, Korea; 4 Division of Bio-Medical Science & Technology, KIST School, Korea University of Science and Technology, Seoul 02792, Korea; 5 Convergence Research Center for Diagnosis, Treatment and Care System of Dementia, Korea Institute of Science and Technology, Seoul 02792, Korea; 6 Department of Chemical & Molecular Engineering, Hanyang University, Ansan, Gyeonggi-do 15588, Korea

**Keywords:** disubstituted pyrimidine, 5-HT_2C_ receptor, cell-based assay, binding affinity, selectivity

## Abstract

Here, we describe the synthesis of disubstituted pyrimidine derivatives and their biological evaluation as selective 5-HT_2C_ agonists. To improve selectivity for 5-HT_2C_ over other subtypes, we synthesized two series of disubstituted pyrimidines with fluorophenylalkoxy groups at either the 5-position or 4-position and varying cyclic amines at the 2-position. The in vitro cell-based assay and binding assay identified compounds **10a** and **10f** as potent 5-HT_2C_ agonists. Further studies on selectivity to 5-HT subtypes and drug-like properties indicated that 2,4-disubstituted pyrimidine **10a** showed a highly agonistic effect on the 5-HT_2C_ receptor, with excellent selectivity, as well as exceptional drug-like properties, including high plasma and microsomal stability, along with low CYP inhibition. Thus, pyrimidine **10a** could be considered a viable lead compound as a 5-HT_2C_ selective agonist.

## 1. Introduction

Serotonin (5-hydroxytryptamine, 5-HT), a monoamine neurotransmitter, plays a critical role in the regulation of various neurological functions, including mood, sleep, cognition, anxiety, sexual behavior, and appetite [1,2]. There are 14 variants of serotonin receptors (5-HT receptors), which mostly belong to G-protein-coupled receptors (GPCRs), except for the 5-HT_3_ receptor, a ligand-gated ion channel. Activation of the 5-HT receptors induces both inhibitory and excitatory signal transduction by modulating the release of many neurotransmitters, such as glutamate, dopamine, epinephrine, and acetylcholine. Therefore, they have been considered crucial therapeutic targets for a variety of neurological diseases, including depression, psychiatric disorders, sexual dysfunction, obesity, and urinary incontinence [3,4,5].

The 5-HT_2_ receptors comprise three subtypes: 5-HT_2A_, 5-HT_2B_, and 5-HT_2C_. Recent studies revealed that activation of 5-HT_2C_ receptors in the central nerve system (CNS) is a potential drug target for effective treatment of schizophrenia, as well as obesity [6,7,8,9,10]. One of the crucial problems faced during the development of 5-HT_2C_ agonists is selectivity over the structurally related 5-HT_2A_ and 5-HT_2B_ receptors. 5-HT_2A_ agonism in humans is implicated in acute adverse effects, such as hallucination and cardiovascular effects. Stimulation of the 5-HT_2B_ receptor is related to other side effects, including chronic cardiac valvulopathy and pulmonary hypertension.

**Figure 1:**
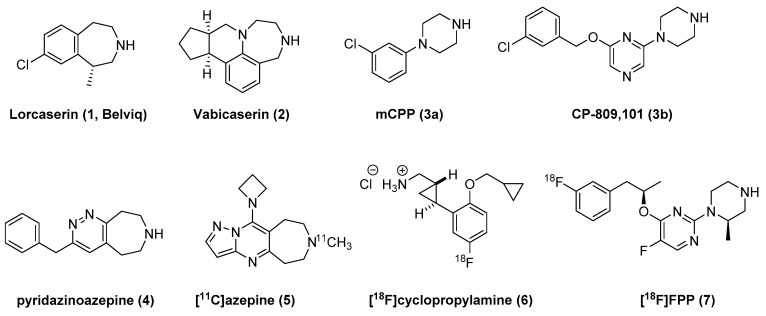
The representative 5-HT_2C_ selective agonists and PET radioligands.

Potent and selective 5-HT_2C_ agonists have been developed, as shown in Figure 1. Lorcaserin **1** was approved by the FDA (Food and Drug Administration) in 2012 for treating obesity [11], and vabicaserin **2** was clinically evaluated as a potential antipsychotic [12]. Numerous 5-HT_2C_ agonists that have undergone preclinical or early clinical studies have been reported [13,14,15,16,17,18,19]. In addition, several 5-HT_2C_-selective positron emission tomography (PET) radioligands (**5** and **6**) have been developed as chemical tools for in vivo imaging of the 5-HT_2C_ receptor [20,21].

**Figure 2:**
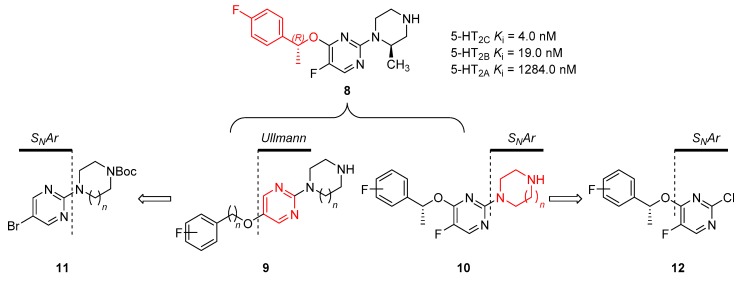
Design of new pyrimidine derivatives as 5-HT_2C_ agonists.

Despite the discovery of potential 5-HT_2C_ agonists, the selectivity of these compounds over other 5-HT_2_ subtypes is still moderate; therefore, the search for highly selective 5-HT_2C_ agonists remains challenging. In this regard, our group has previously reported optically active pyrimidine derivative **8** as a potent and selective 5-HT_2C_ agonist (Figure 2) [22]. The structure activity relationship (SAR) between related pyrimidine derivatives and 5-HT_2_ subtypes revealed that a subtle change in the fluorophenylalkoxy moiety attached to pyrimidine **8** was essential to control the activation of selectivity for 5-HT_2C_ over 5-HT_2A_ and 5-HT_2B_. Although compound **8** was discovered as a lead compound with excellent potency and good selectivity for the 5-HT_2_ subtypes, further structural modification of these pyrimidine derivatives is required to improve selectivity over other 5-HT subtypes. Furthermore, selective 5-HT_2C_ agonists could be applied in the development of chemical probes such as PET radiotracers [23,24,25,26].

We first planned to alter the substitution pattern of pyrimidines for the structural adjustment of compound **8** toward the 5-HT_2C_ binding pocket. Recent studies demonstrated that pyridazine-fused azepine **4** containing a remote phenyl group (Figure 1) showed high potency and selectivity to 5-HT_2C_ over 5-HT_2B_ with a good stability profile [19]. Accordingly, we hypothesized that the linear form of 2,5-disubstituted pyrimidine **9,** possessing a flexible fluorophenylalkoxy group at the 5-position, might be suitable for exploring the relationship between structural change and selectivity (Figure 2). Based on the molecular docking study of several 5-HT_2C_ agonists published in literature [27,28], we also assumed that the terminal-free amine of the piperazine moiety could be a key functional group binding to a conserved residue, D134, in 5-HT_2C_ protein. Taken together with the fact that compound **8** derived from (*R*)-isomer of secondary benzyl alcohol showed better binding affinity and selectivity to 5-HT_2C_ than its diastereomer [22], we designed an alternative series of pyrimidine derivatives **10** possessing a variety of cyclic amines to examine their agonistic effect on 5-HT receptor activation. Herein, we report the synthesis of disubstituted pyrimidine derivatives with different substitution patterns and their biological evaluation as selective 5-HT_2C_ receptor agonists.

## 2. Results and Discussion

### 2.1. Synthesis of 2,5-Disubstituted Pyrimidine Derivatives

**Scheme 1:**
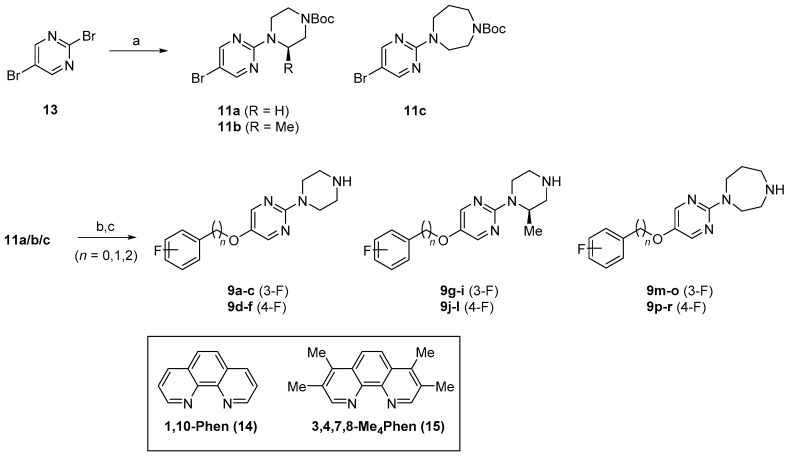
Synthesis of 2,5-disubstituted pyrimidine derivatives **9a–r**. Reagents and conditions: (**a**) *N*-Boc-protected amines, K_2_CO_3_, CH_3_CN, 80 °C, 88–97%; (**b**) 3- or 4-fluorophenol/benzyl alcohol/phenethyl alcohol, CuI or CuO_2_, **14** or **15**, Cs_2_CO_3_, toluene, 110 °C, 42–85%; (**c**) TFA, CH_2_Cl_2_, 0 °C, 52–67%.

Initially, we synthesized 2,5-disubstituted pyrimidines with flexible phenylalkoxy groups at the 5-position, as shown in Scheme 1. Starting from 2,5-dibromopyrimidine **13**, three kinds of 2-aminopyrimidines **11a–c** were prepared through S_N_Ar type amination. Pyrimidine derivatives **11a, 11b,** and **11c** were reacted with 3- or 4-fluoro-phenols, benzyl alcohols, and phenethyl alcohols in the presence of copper catalyst and 1,10-phenanthroline ligands **14** or **15** [29,30,31] to afford 2,5-disubstituted pyrimidines possessing an *N*-Boc-protecting group at the terminal amine. It is noted that the use of **15** as a ligand in this transformation led to formation of the desired products in higher yields. Finally, the 2-amino-5-alkoxypyrimidines **9a–r** were obtained after removal of the Boc group under acidic conditions.

### 2.2. Synthesis of 2,4-Disubstituted Pyrimidine Derivatives

**Scheme 2:**
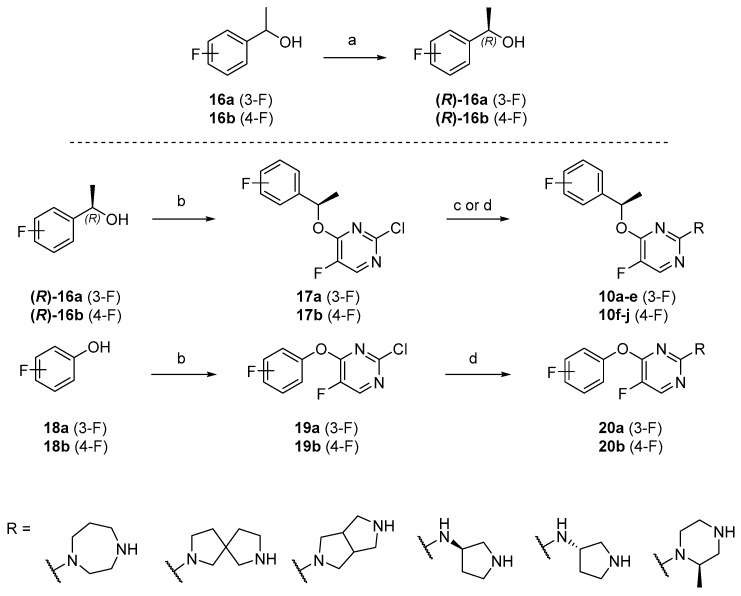
Synthesis of 2,4-disubstituted pyrimidine derivatives **10a–j** and **20ab**. Reagents and conditions: (**a**) (i) Vinyl acetate (0.4 eq), CAL-B, pyridine, hexane, rt, 66–67%; (ii) 1 M NaOH, MeOH, rt, 78%; (**b**) 2,4-dichloro-5-fluoropyrimidine, *t-*BuONa, toluene, 0 °C, 51–93%; (**c**) amines, Et_3_N, toluene (0.5 M), 100 °C, 12 h, 61–98%; (**d**) (i) *N*-Boc-protected amines, Et_3_N, DMSO (0.5 M), 100 °C, 12 h, 44–94%; (ii) HCl (4.0 M in dioxane), CH_3_CN, 0 °C or TFA, CH_2_Cl_2_, 0 °C.

The synthesis of a series of 2,4-disubstituted pyrimidines **10**, possessing different cyclic amines, is described in Scheme 2. According to our protocol [22], we efficiently synthesized optically active 1-(3- or 4-fluorophenyl)ethan-1-ol (*R*)-**16a** and (*R*)-**16b** using an enzymatic kinetic resolution method. As per the procedure previously reported in literature [32], nucleophilic aromatic substitution (S_N_Ar) of 2,4-dichloropyrimidine with alcohols (*R*)-**16a** and (*R*)-**16b** selectively produced 4-alkoxypyrimidines **17a** and **17b**, which were subjected to an additional S_N_Ar reaction to afford 2-alkoxy-4-aminopyrimidines as free or *N*-Boc-protected amine forms. The latter were further treated with a strong acid, such as HCl or TFA, to give the desired disubstituted pyrimidines. Besides pyrimidine analogues **10**, we synthesized compounds **20a** and **20b** possessing a direct connection between the fluorophenyl group and pyrimidine via ether linkage using a similar experimental process. In this case, these compounds **20a/b** might allow us to examine the optimal size of the fluorophenylalkoxy group through a comparison of their in vitro activity with that of **8**.

### 2.3. In Vitro Evaluation of Pyrimidine Derivatives

In vitro agonistic activities of pyrimidines against 5-HT_2C_ were evaluated using a fluorescence-based receptor functional assay, and the results are summarized in Table 1. Among the first series of pyrimidines possessing different sizes of phenylalkoxy groups at the 5-position, we found that the cellular agonistic effect of only compound **9b** was higher than 50% at a concentration of 10 μM. Regarding the effect of cyclic amines at the 2-position of pyrimidine, only compounds **9g–l** with methyl piperazine showed low to moderate activities, with up to 48% activation. Based on this result, we concluded that the substitution pattern of cyclic amines and the fluorophenylalkoxy group on the pyrimidine ring is important for 5-HT_2C_ agonism. Thus, we decided not to proceed with further biological evaluation of this first series of compounds because an activation value higher than 50% is considered to represent significant agonistic effects of the compounds.

Next, the in vitro biological activities of 2,4-disubstituted pyrimidines **10a–j** and **20a–b** against the 5-HT_2C_ receptor were evaluated, as shown in Table 2. At this point, the ligand binding assay, as well as the cell-based functional assay, were performed to identify more potent and selective 5-HT_2C_ agonists. Among the tested compounds, four pyrimidine derivatives showed greater than 50% activation against 5-HT_2C_ in the cell-based assay. Studies on the binding affinity of this series to 5-HT_2C_ indicated that compounds **10a** and **10f**, with 1,4-diazepane at the 2-position of pyrimidine, exhibited excellent *K*i values of 7.9 nM and 19.0 nM, respectively. Although the result of the cell-based assay is important to explore the cellular function of the 5-HT_2C_ receptor, binding affinity plays a key role in determining whether the compound is suitable for the development of a 5-HT_2C_-specific chemical probe. Thus, we selected compounds **10a** and **10f** for further examination of the 5-HT subtype selectivity and drug-like properties.

The evaluation of compounds **10a** and **10f** for binding affinity to the 5-HT receptor subtypes is illustrated in Table 3. We calculated the selectivity index (SI) of binding affinities between 5-HT_2C_ and other subtypes by dividing the given subtype *K*i value with the 5-HT_2C_ *K*i value. The results indicate that compound **10a** displayed higher selectivity for most 5-HT subtypes compared to compound **10f**, except for 5-HT_1B_ and 5-HT_1E_, to which the binding affinities of both compounds were negligible. Most importantly, compound **10a** showed good selectivity for 5-HT_2C_ over 5-HT_2A_ and 5-HT_2B_, which are associated with the primary side effects of non-specific 5-HT_2C_ agonists.

Considering the 5-HT_2C_ cellular agonistic effect, binding affinity, and subtype selectivity of **10a**, we further investigated its drug-like properties, such as plasma stability, microsomal stability, and CYP inhibition, as shown in Table 4. For plasma stability, 10 μM of compound **10a** was incubated in human plasma; the remaining amount of **10a** was measured after incubation for 30 min and 2 h. For microsomal stability, human liver microsomes were treated with **10a** at a concentration of 1 μM; the % remaining amount of **10a** was analyzed at 30 min after treatment. CYP inhibition values against five human CYP450 isozymes were recorded as % remaining activity values of the given isozymes. We found that compound **10a** was stable in both the plasma and liver, indicating that compound **10a** would be less affected by hepatic metabolism or plasma decomposition throughout systemic circulation. Furthermore, the inhibitory activity of **10a** against all the tested CYP isozymes was significantly low (%remaining activity of isozymes >60%), suggesting that compound **10a** might not exert toxic effects due to drug–drug interaction (DDI). Overall, compound **10a** exhibited considerably excellent plasma and microsomal stability with low CYP inhibitory activity.

### 2.4. Molecular Docking Study

In order to investigate the good selectivity of **10a** to 5-HT_2C_ over 5-HT_2B_, the molecular docking study was performed using Discovery Studio software. Thus, the interaction of **10a** and **8** with those receptors were evaluated using the crystal structures of 5-HT_2C_ (PDB ID = 6BQH) and 5-HT_2B_ (PDB ID = 4IB4), which were recently reported in the literatures [28,33].

**Figure 3:**
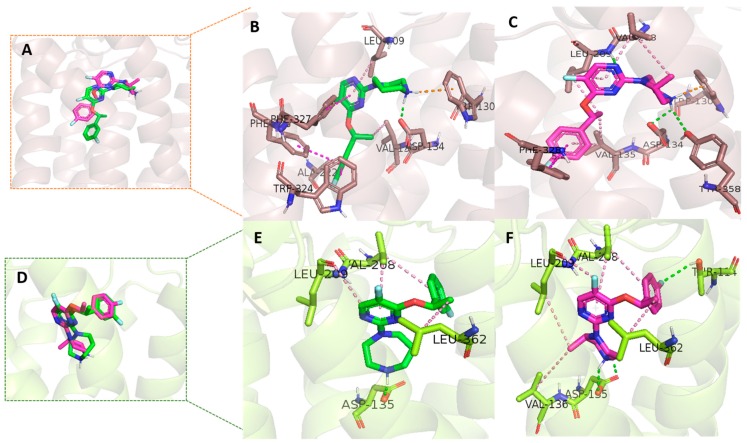
Interactions of **10a** and **8** inside the 5-HT_2C_ and 5-HT_2B_ receptors. Ligands **10a** and **8** are shown in green and magenta color stick format. 5-HT_2C_ (wheat color) and 5-HT_2B_ (green color) protein structures are shown in cartoon format. Hydrogen bond, π-cationic, π–π stacking, and hydrophobic interactions are shown in green, orange, dark pink, and light pink dash line format. (**A**) Overlap of **10a** and **8** inside 5-HT_2C_ protein. (**B**) Interactions between ligand **10a** and the 5-HT_2C_ receptor. (**C**) Interactions between ligand **8** and the 5-HT_2C_ receptor. (**D**) Overlap of **10a** and **8** inside 5-HT_2B_ protein. (**E**) Interactions between ligand **10a** and the 5-HT_2B_ receptor. (**F**) Interactions between ligand **8** and the 5-HT_2B_ receptor.

In the case of 5-HT_2C_ receptor docking study, the binding orientation of both ligands **10a** (-CDOCKER score 36.31) and **8** (-CDOCKER score 36.96) are similar but not precisely overlapping over each other (Figure 3A). Both of these ligands have shown hydrogen bonds with crucial aspartic acids Asp134 (Figure 3B,C). NH groups of piperazine (**8**) and 1,4-diazepane ring (**10a**) are involved in hydrogen bonds and π-cationic interactions with Trp130 and Asp134. Additionally, **8** has shown two more hydrogen bonds with Tyr358 and Leu209. The hydrogen of the NH group of the piperazine ring has shown a hydrogen bond with Tyr358 and nitrogen of the pyrimidine ring has shown one hydrogen bond with Leu209. In total, we recognized four hydrogen bond interactions between 5-HT_2C_ and **8**. Fluorophenyl rings of **8** were involved in π–π stacking interactions with Phe328 residue. In the case of **10a**, in addition to hydrogen bonds, strong π–π stacking contacts were observed with Phe327, phe328, and Trp324. Several hydrophobic contacts in both ligands were identified, mediated by Val135 and Leu209 in **10a** and Val135, Val208, and Leu209 in **8**.

In the case of 5-HT2B receptor docking study, overlap of ligand **10a** (-CDOCKER score 37.40) and **8** (-CDOCKER score 39.53) inside 5-HT_2B_ binding pocket (Figure 3D) shows that ligand bind at the same place but differ in pyrimidine, fluorophenyl ring, and diazepane ring/piperidine orientation. Compound **8** has shown three hydrogen bonds with binding site residues. The hydrogen (NH group) of the piperazine ring showed two hydrogen bonds with Asp135 (Figure 3F). The fluorine group on the phenyl ring in **8** has shown an additional hydrogen bond with Thr114. Beside these hydrogen bonding interactions, several hydrophobic residues, Val136, Val208, Leu209, and Leu362, hold the molecule by several hydrophobic interactions. Compound **10a** has only one hydrogen bond with Asp135 (Figure 3E). Compared to **8**, **10a** has fewer hydrophobic contacts with the 5-HT_2B_ receptor binding pocket (Val2018, Leu209, and Leu362).

Although both **10a** and **8** bind in a similar way inside the 5-HT_2B_ binding pocket, a minor difference in aromatic ring orientation affects the hydrophobic interactions of **10a**. The difference in fluorophenyl ring orientation is responsible for the additional hydrogen bond between Thr114 and **8**. These extra hydrogen bonds and hydrophobic contacts inside the 5-HT_2B_ receptors make the potency of compound **8** superior to that of compound **10a** in the 5-HT_2B_ binding assay. Compared to the 5-HT_2B_ receptor, **10a** has stronger interactions (hydrogen bond and hydrophobic) with the 5-HT_2C_ receptor. Overall, the decrease in hydrophobic contacts inside the 5-HT_2B_ receptor makes our molecule **10a** more selective towards the 5-HT_2C_ receptor.

## 3. Materials and Methods

### 3.1. General Methods

All reactions were conducted under oven-dried glassware, under an atmosphere of nitrogen. All commercially available reagents were purchased and used without further purification. Solvents and gases were dried according to standard procedures. Organic solvents were evaporated with reduced pressure using a rotary evaporator. Reactions were followed by analytical thin layer chromatography (TLC) analysis using glass plates precoated with silica gel (0.25 mm). TLC plates were visualized by exposure to UV light (UV), and then were visualized with a KMnO_4_ or *p*-anisaldehyde stain followed by brief heating on a hot plate. Flash column chromatography was performed using silica gel 60 (230–400 mesh, Merck) with the indicated solvents. ^1^H-NMR spectra were measured with 400 MHz and ^13^C-NMR spectra were measured with 100 MHz using CDCl_3_ and CD_3_OD. ^1^H NMR spectra are represented as follows: Chemical shift, multiplicity (s = singlet, d = doublet, t = triplet, q = quartet, m = multiplet), integration, and coupling constant (*J*) in Hertz (Hz). ^1^H NMR chemical shifts are reported relative to CDCl_3_ (7.26 ppm). ^13^C NMR was recorded relative to the central line of CDCl_3_ (77.0 ppm). High resolution mass spectra (LR-MS) were obtained using positive electrospray ionization and mass/charge (*m/z*) ratios are reported as values in atomic mass units.

### 3.2. Synthesis of 2,5-Disubstituted Pyrimidines

#### 3.2.1. General Procedure for Preparing Compounds 11a–c

In a dry sealed tube under argon were placed, to a solution of 1-Boc-piperazine (5.04 mmol), potassium carbonate (13.1 mmol) in acetonitrile (12.6 mL) was added 2,5-dibromo pyrimidine **13** (5.04 mmol). The mixture was allowed to stir at 80 °C for 12 h. After completion of the reaction (monitored by TLC), the mixture was then cooled at room temperature, then it was quenched with saturated aqueous NH_4_Cl (10 mL) and extracted with EtOAc. The organic layers were dried over anhydrous MgSO_4_ and concentrated in vacuo. The resulting residue was purified by flash column chromatography on silica gel (EtOAc:*n*-hexane = 1:8) to afford piperazine **11a**.

*tert-Butyl 4-(5-bromopyrimidin-2-yl)piperazine-1-carboxylate* (**11a**): Yield: 88%; ^1^H-NMR (400 MHz, CDCl_3_) *δ* 8.30 (s, *J* = 2.0 Hz, 2H), 3.78-–3.75 (t, *J* = 5.2 Hz, 4H), 3.49–3.47 (t, *J* = 5.2 Hz, 4H), 1.48 (s, 9H). ^13^C-NMR (100 MHz, CD_3_OD) *δ* 161.2, 159.2, 156.46, 107.15, 81.5, 44.8, 28.6.

*tert-Butyl (R)-4-(5-bromopyrimidin-2-yl)-3-methylpiperazine-1-carboxylate* (**11b**): Yield: 92%; ^1^H-NMR (400 MHz, CDCl_3_) *δ* 8.28 (s, *J* = 2.0 Hz, 1H), 4.81–4.70 (m, 1H), 4.39–4.36 (m, 1H), 4.15–3.90 (m, 2H), 3.19–3.08 (m, 1H), 3.08–2.90 (m, 2H), 1.47 (s, 9H), 1.16 (d, *J* = 6.7 Hz, 3H). ^13^C-NMR (100 MHz, CD_3_OD) *δ* 160.9, 159.2, 156.9, 107.0, 81.4, 45.1, 44.0, 39.4, 14.5.

*tert-Butyl 4-(5-bromopyrimidin-2-yl)-1,4-diazepane-1-carboxylate* (**11c**): Yield: 97%; ^1^H-NMR (400 MHz, CDCl_3_) *δ* 8.28 (s, 2H), 3.86–3.80 (m, 2H), 3.73–3.70 (t, *J* = 6.1 Hz, 2H), 3.55–3.52 (t, *J* = 5.4 Hz, 2H), 3.38–3.35 (t, *J* = 5.9 Hz, 1H), 3.28–3.25 (t, *J* = 6.1 Hz, 1H), 1.96–1.90 (m, 2H), 1.43 (d, *J* = 8.4 Hz, 9H). ^13^C-NMR (100 MHz, CDCl_3_) *δ* 159.5, 158.1, 155.3, 105.8, 79.6, 49.0, 48.5, 47.4, 46.9, 46.3, 46.1, 28.5, 25.4.

#### 3.2.2. General Procedure for Preparing Compounds 9a–r

In a dry sealed tube under argon were placed **11b** (0.56 mmol), 3-fluorobenzyl alcohol (1.68 mmol), cesium carbonate (0.84 mmol), copper iodide (0.056 mmol) (or copper(I) oxide), 1,10-phenanthroline (0.11 mmol) (or 3,4,7,8-tetramethyl-1,10-phenanthroline) in toluene (1.1 mL) and the mixture was heated at 130 °C for 20 h. After completion of the reaction (monitored by TLC), the mixture was then cooled at room temperature and it was quenched with saturated aqueous NH_4_Cl (3 × 50 mL), extracted with EtOAc. The organic layers were dried over anhydrous MgSO_4_ and concentrated in vacuo. The resulting residue was purified by flash column chromatography on silica gel (EtOAc: Acetone: Hexane = 1:1:15) to afford intermediate **pre-9h**.

To a solution of methyl piperazine carboxylate **pre-9h** (0.136 mmol) in dichloromethane (1.3 mL) was added trifluoroacetic acid (0.21 mL). The mixture was allowed to stir at 0 °C for 3 h. After completion of the reaction (monitored by TLC), then neutralized by sodium hydrogen carbonate until the mixture to be pH 8 and extracted with EtOAc. The organic layers were dried over anhydrous MgSO_4_ and concentrated in vacuo. The resulting residue was purified by flash column chromatography on silica gel (MeOH:DCM = 1:15) to afford pyrimidine **9h**, which was isolated as HCl salt form after treating with 4 M HCl in diethyl ether.

*tert-Butyl 4-(5-((3-fluorophenoxy)pyrimidin-2-yl)piperazine-1-carboxylate* (**pre-9a**): Yield: 21%; ^1^H-NMR (400 MHz, CDCl_3_) *δ* 8.17 (s, 2H), 7.27–7.21 (m, 1H), 6.77–6.73 (m, 1H), 6.71–6.62 (m, 2H), 3.80 (t, *J* = 5.1 Hz, 4H), 3.52 (t, *J* = 5.0 Hz, 4H), 1.49 (s, 9H).

*4-(5-((3-fluorophenoxy)pyrimidin-2-yl)piperazin-1-ium∙hydrochloride* (**9a**): Yield: 21%; ^1^H-NMR (400MHz, CDCl_3_) *δ* 8.17 (s, 2H), 7.24 (dd, *J* = 15.4, 7.5 Hz, 1H), 6.75 (m, 1H), 6.70 (d, *J* = 8.4 Hz, 1H), 6.64 (d, *J* = 10.2 Hz, 1H), 3.89 (t, *J* = 4.5 Hz, 4H), 3.07 (t, *J* = 4.7 Hz, 4H). ^13^C-NMR (100 MHz, CD_3_OD) *δ* 162.4 (d, ^1^*J* = 259 Hz), 159.7, 152.3, 152.0, 144.5, 132.2 (d, ^3^*J* = 9 Hz), 113.4 (d, ^4^*J* = 3 Hz), 110.9 (d, ^2^*J* = 21 Hz), 105.4 (d, ^2^*J* = 25 Hz), 44.4, 42.5; LRMS-EI (*m/z*): [M–Cl]^+^ calcd for C_14_H_15_FN_4_O: 274.30, found: 274.30

*tert-Butyl 4-(5-((3-fluorobenzyl)oxy)pyrimidin-2-yl)piperazine-1-carboxylate***(pre-9b):** Yield: 79%; ^1^H-NMR (400 MHz, CDCl3) δ 8.11 (s, 2H), 7.37–7.32 (m, 1H), 7.17–7.11 (m, 2H), 7.05–6.99 (m, 1H), 5.01 (s, 2H), 3.71 (t, *J* = 5.2 Hz, 4H), 3.49 (t, *J* = 5. 2 Hz, 4H), 1.48 (s, 9H).

*4-(5-((3-fluorobenzyl)oxy)pyrimidin-2-yl)piperazin-1-ium∙hydrochloride* (**9b**): Yield: 58%; ^1^H-NMR (400 MHz, CD_3_OD) *δ* 8.23 (s, 1H), 7.42–7.37 (m, 1H), 7.25–7.17 (m, 2H), 7.06 (td, *J* = 8.5, 2.4 Hz, 1H), 7.07–7.02 (m, 1H), 5.12 (s, 2H), 3.95 (t, *J* = 5.3 Hz, 4H), 3.23 (t, *J* = 5.3 Hz, 4H). ^13^C-NMR (100 MHz, CD_3_OD) *δ* 164.3 (d, ^1^*J* = 243 Hz), 158.2, 147.7, 147.4, 140.8 (d, ^3^*J* = 7 Hz), 131.5 (d, ^3^*J* = 8 Hz), 124.4 (d, ^4^*J* = 3 Hz), 115.8 (d, ^2^*J* = 21 Hz), 115.3 (d, ^2^*J* = 22 Hz), 71.9, 44.3, 42.6; LRMS-EI (*m/z*): [M–Cl]^+^ calcd for C_15_H_17_FN_4_O: 288.33, found: 288.33

*tert-Butyl 4-(5-((3-fluorophenethoxy)pyrimidin-2-yl)piperazine-1-carboxylate* (**pre-9c**): Yield: 46%; ^1^H-NMR (400 MHz, CDCl_3_) *δ* 8.05 (s, 1H), 7.24–7.20 (m, 2H), 7.02–6.98 (m, 2H), 4.12 (t, *J* = 6.8 Hz, 2H), 3.69 (t, *J* = 5.2 Hz, 2H), 3.48 (t, *J* = 5.2 Hz, 2H), 3.03 (t, *J* = 6.8 Hz, 2H), 1.48 (s, 9H).

*4-(5-((3-fluorophenethoxy)pyrimidin-2-yl)piperazin-1-ium∙hydrochloride* (**9c**): Two-step yield: 46%; ^1^H-NMR (400 MHz, CD_3_OD) *δ* 8.17 (s, 2H), 7.34–7.29 (m, 1H), 7.13 (d, *J* = 8 Hz, 1H), 7.07 (d, *J* = 10.1 Hz, 1H), 6.96 (td, *J* = 8.7, 2.3 Hz, 1H), 4.25 (t, *J* = 6.5 Hz, 2H), 3.97 (t, *J* = 5.2 Hz, 4H), 3.27 (t, *J* = 5.2 Hz, 4H), 3.09 (t, *J* = 6.5 Hz, 2H). ^13^C-NMR (100 MHz, CD_3_OD) *δ* 164.3 (d, ^1^*J* = 242 Hz), 158.1, 148.2, 146.8, 142.5 (d, ^3^*J* = 7 Hz), 131.1 (d, ^3^*J* = 8 Hz), 125.9 (d, ^4^*J* = 3 Hz), 116.7 (d, ^2^*J* = 21 Hz), 114.3 (d, ^2^*J* = 21 Hz), 71.28, 44.3, 42.7, 36.4, 36.4; LRMS-EI (*m/z*): [M–Cl]^+^ calcd for C_16_H_19_FN_4_O: 302.35, found: 302.35

*tert-Butyl 4-(5-((4-fluorophenoxy)pyrimidin-2-yl)piperazine-1-carboxylate* (**pre-9d**): Yield: 21%; ^1^H-NMR (400 MHz, CDCl_3_) *δ* 8.17 (s, 2H), 7.03–6.99 (m, 2H), 6.92–6.89 (m, 2H), 3.80 (t, *J* = 5.2 Hz, 4H), 3.52 (t, *J* = 5.2 Hz, 4H), 1.49 (s, 9H).

*4-(5-((4-fluorophenoxy)pyrimidin-2-yl)piperazin-1-ium∙hydrochloride* (**9d**): Yield: 60%; ^1^H-NMR (400 MHz, CDCl_3_) *δ* 8.15 (s, 2H), 7.02-6.98 (m, 2H), 6.91–6.88 (m, 2H), 3.77 (t, *J* = 5.2 Hz, 4H), 3.51 (t, *J* = 5.1 Hz, 4H). ^13^C-NMR (100 MHz, CD_3_OD) *δ* 158.9 (d, ^1^*J* = 241 Hz), 157.9, 153.8, 150.1, 144.4, 118.6 (d, ^3^*J* = 8 Hz), 116.6 (d, ^2^*J* = 23 Hz), 43.4, 41.5; LRMS-EI (*m/z*): [M–Cl]^+^ calcd for C_14_H_15_FN_4_O: 274.30, found: 274.30.

*tert-Butyl 4-(5-((4-fluorobenzyl)oxy)pyrimidin-2-yl)piperazine-1-carboxylate* (**pre-9e**): Yield: 76%; ^1^H-NMR (400 MHz, CDCl_3_) *δ* 8.10 (s, 1H), 7.37 (dd, *J* = 8.7, 5.3 Hz, 2H), 7.09–7.05 (m, 2H), 4.98 (s, 2H), 3.71 (t, *J* = 5.2 Hz, 4H), 3.49 (t, *J* = 5.2 Hz, 4H), 1.48 (s, 9H).

*4-(5-((4-fluorobenzyl)oxy)pyrimidin-2-yl)piperazin-1-ium∙hydrochloride* (**9e**): Two-step yield: 67%; ^1^H-NMR (400 MHz, CD_3_OD) *δ* 8.22 (s, 2H), 7.47–7.43 (m, 2H), 7.13–7.08 (m, 2H), 5.08 (s, *J* = 2.5 Hz, 2H), 3.95 (t, *J* = 5.3 Hz, 4H), 3.24 (t, *J* = 5.2 Hz, 4H). ^13^C-NMR (100 MHz, CD_3_OD) *δ* 164.0 (d, ^1^*J* = 243 Hz), 158.4, 147.4, 134.0 (d, ^4^*J* = 3 Hz), 132.3, 131.0 (d, ^3^*J* = 8 Hz), 116.3 (d, ^2^*J* = 22 Hz), 72.1, 44.7, 43.3; LRMS-EI (*m/z*): [M**–**Cl]^+^ calcd for C_15_H_17_FN_4_O: 288.33, found: 288.33.

*tert-Butyl 4-(5-((4-fluorophenethoxy)pyrimidin-2-yl)piperazine-1-carboxylate* (**pre-9f**): Yield: 54%; ^1^H-NMR (400 MHz, CDCl_3_) *δ* 8.05 (s, 1H), 7.24–7.20 (m, 2H), 7.02–6.98 (m, 2H), 4.12 (t, *J* = 6.8 Hz, 2H), 3.70 (t, *J* = 5.2 Hz, 2H), 3.48 (t, *J* = 5.2 Hz, 2H), 3.03 (t, *J* = 6.8 Hz, 2H), 1.48 (s, 9H).

*4-(5-((4-fluorophenethoxy)pyrimidin-2-yl)piperazin-1-ium∙hydrochloride* (**9f**): Two-step yield: 53%; ^1^H-NMR (400 MHz, CD_3_OD) *δ* 8.16 (s, 2H), 7.32–7.28 (m, 2H), 7.04–6.99 (m, 2H), 4.21 (t, *J* = 6.6 Hz, 2H), 3.95 (t, *J* = 5.1 Hz, 4H), 3.27 (t, *J* = 5.1 Hz, 4H), 3.05 (t, *J* = 6.6 Hz, 2H). ^13^C-NMR (100 MHz, CD_3_OD) *δ* 163.1 (d, ^1^*J* = 241 Hz), 158.6, 147.7, 146.8, 135.51, 131.7 (d, ^3^*J* = 8 Hz), 116.0 (d, ^2^*J* = 21 Hz), 71.7, 45.3, 44.5, 35.9; LRMS-EI (*m/z*): [M–Cl]^+^ calcd for C_16_H_19_FN_4_O: 302.35, found: 302.35.

*tert-Butyl (R)-4-(5-((3-fluorophenoxy)pyrimidin-2-yl)-3-methylpiperazine-1-carboxylate* (**pre-9g**): Yield: 17%; ^1^H-NMR (400 MHz, CDCl_3_) *δ* 8.18 (s, 2H), 7.27–7.22 (m, 1H), 6.78–6.70 (m, 2H), 6.65–6.63 (m, 1H), 4.90–4.84 (m, 1H), 4.43–4.40 (m, 1H), 4.18–3.93 (m, 2H), 3.24–3.18 (m, 1H), 3.13–2.94 (m, 2H), 1.49 (s, 9H), 1.20 (d, *J* = 6.8 Hz, 3H).

*(R)-4-(5-((3-fluorophenoxy)pyrimidin-2-yl)-3-methylpiperazine-1-ium∙hydrochloride* (**9g**): Yield: 21%; ^1^H-NMR (400 MHz, CDCl_3_) *δ* 8.18 (s, 2H), 7.27–7.21 (m, 1H), 6.77–6.65 (m, 2H), 6.63–6.62 (m, 1H), 4.78–4.75 (m, 1H), 4.41–4.38 (m, 1H), 3.16–3.08 (m, 2H), 3.04–3.00 (m, 1H), 2.92–2.89 (m, 1H), 2.83–2.76 (m, 1H), 1.28 (d, *J* = 6.8 Hz, 3H). ^13^C-NMR (100 MHz, CD_3_OD) *δ* 159.3, 152.1, 146.9, 144.3, 132.2 (d, ^3^*J* = 9 Hz), 131.8, 113.4, 110.8 (d, ^2^*J* = 21 Hz), 105.4 (d, ^2^*J* = 25 Hz), 45.8, 44.8, 36.9, 35.9, 13.7; LRMS-EI (*m/z*): [M**–**Cl]^+^ calcd for C_15_H_17_FN_4_O: 288.33, found: 288.33.

*tert-Butyl (R)-4-(5-((3-fluorobenzyl)oxy)pyrimidin-2-yl)-3-methylpiperazine-1-carboxylate* (**pre-9h**): Yield: 43%; ^1^H-NMR (400 MHz, CDCl_3_) *δ* 8.11 (s, 2H), 7.37–7.32 (m, 1H), 7.15–7.11 (m, 2H), 7.04–6.99 (m, 1H), 5.00 (s, 2H), 4.75 (s, 1H), 4.31 (d, *J* = 12.5 Hz, 1H), 4.10 (d, *J* = 12.5 Hz, 1H, broad), 3.17–3.10 (m, 1H), 3.10–2.91 (brs, 1H), 1.48 (s, 9H), 1.14 (d, *J* = 6.7 Hz, 3H).

*(R)-4-(5-((3-fluorobenzyl)oxy)pyrimidin-2-yl)-3-methylpiperazine-1-ium∙hydrochloride* (**9h**): Yield: 70%; ^1^H-NMR (400 MHz, CDCl_3_) *δ* 8.12 (s, 2H), 7.37–7.33 (m, 1H), 7.17–7.11 (m, 2H), 7.05–7.00 (td, *J* = 8.4, 2.3 Hz, 1H), 5.01 (s, 1H), 4.85–4.82 (m, 1H), 4.45–4.40 (m, 1H), 3.23–3.17 (m, 2H), 3.10–3.01 (m, 2H), 2.89–2.82(m, 1H), 1.30 (d, *J* = 6.9 Hz, 3H). ^13^C-NMR (100 MHz, CD_3_OD) *δ* 165.6 (d, ^1^*J* = 244 Hz), 157.8, 147.4, 140.8, 131.4 (d, ^3^*J* = 9 Hz), 124.3, 115.8 (d, ^2^*J* = 20 Hz), 115.3 (d, ^2^*J* = 22 Hz), 71.9, 45.8, 44.8, 37.2, 13.4; LRMS-EI (*m/z*): [M**–**Cl]^+^ calcd for C_16_H_19_FN_4_O: 302.35, found: 302.35

*tert-Butyl (R)-4-(5-((3-fluorophenethoxy)pyrimidin-2-yl)-3-methylpiperazine-1-carboxylate* (**pre-9i**): Yield: 24%; ^1^H-NMR (400 MHz, CDCl_3_) *δ* 8.06 (s, 2H), 7.30–7.26 (m, 1H), 7.04–7.02 (m, 1H), 6.98–6.91 (m, 2H), 4.98 (s, 2H), 4.80–4.70 (m, 1H), 4.31–4.28 (m, 1H), 4.14 (t, *J* = 6.7 Hz, 2H), 3.97–3.90 (m, 2H), 3.17–3.10 (m, 2H), 3.06 (t, *J =* 6.7 Hz, 2H), 3.00–2.80 (m. 2H), 1.48 (s, 9H), 1.14 (d, *J* = 6.7 Hz, 3H).

*(R)-4-(5-((3-fluorophenethoxy)pyrimidin-2-yl)-3-methylpiperazin-1-ium∙hydrochloride* (**9i**): Two-step yield: 37%; ^1^H-NMR (400 MHz, CDCl_3_) *δ* 8.06 (s, 2H), 7.28 (dd, *J* = 13.9, 7.9 Hz, 1H), 7.03 (d, *J* = 7.6 Hz, 1H), 6.98–6.91 (m, 2H), 4.86 (s, 2H), 4.45 (d, *J* = 12.4 Hz, 1H), 4.15 (t, *J* = 6.7 Hz, 2H), 3.28–3.14 (m, 2H), 3.11–3.08 (m, 1H), 3.06 (t, *J* = 6.7 Hz, 2H), 2.87 (td, *J* = 12.6, 4.1 Hz, 2H), 1.26 (d, *J* = 6.9 Hz, 2H). ^13^C-NMR (100 MHz, CD_3_OD) *δ* 163.10 (d, ^1^*J* = 245 Hz), 157.6, 147.9, 146.1, 142.5 (d, ^3^*J* = 7 Hz), 131.1 (d, ^3^*J* = 8 Hz), 125.9 (d, ^4^*J* = 3 Hz), 116.7 (d, ^2^*J* = 21 Hz), 114.2 (d, ^2^*J* = 21 Hz), 71.3, 45.6, 44.6, 36.7, 36.4, 30.9, 13.3; LRMS-EI (*m/z*): [M**–**Cl]^+^ calcd for C_17_H_21_FN_4_O: 316.38, found: 316.38.

*tert-Butyl (R)-4-(5-((4-fluorophenoxy)pyrimidin-2-yl)-3-methylpiperazine-1-carboxylate* (**pre-9j**): Yield: 27%; ^1^H-NMR (400 MHz, CDCl_3_) *δ* 8.17 (s, 2H), 7.02–6.98 (m, 2H), 6.91–6.89 (m, 2H), 4.90–4.83 (m, 1H), 4.43–4.39 (m, 1H), 4.18–3.92 (m, 3H), 3.25–3.18 (m, 1H), 3.15–2.93 (m, 2H), 1.49 (s, 9H), 1.20 (d, *J* = 6.7 Hz, 3H).

*(R)-4-(5-((4-fluorophenoxy)pyrimidin-2-yl)-3-methylpiperazine-1-ium∙hydrochloride* (**9j**): Yield: 39%; ^1^H-NMR (400 MHz, CDCl_3_) *δ* 8.15 (s, 2H), 7.01–6.96 (m, 2H), 6.91–6.87 (m, 2H), 4.79–4.76 (m, 1H), 4.42–4.38 (m, 1H), 3.16–3.09 (m, 2H), 3.05–3.01 (m, 1H), 2.94–2.91 (m, 1H), 2.84–2.77 (m, 1H), 2.77–2.70 (m, 1H), 1.28 (d, *J* = 6.8 Hz, 3H). ^13^C-NMR (100 MHz, CD3OD) *δ* 160.1 (d, ^1^*J* = 239 Hz), 159.0, 155.5, 155.5 (d, ^4^*J* = 2 Hz), 145.5, 119.8 (d, ^3^*J* = 8 Hz), 117.4 (d, ^2^*J* = 24 Hz), 45.9, 44.9, 37.2, 30.9, 30.7, 13.5; LRMS-EI (*m/z*): [M**–**Cl]^+^ calcd for C_15_H_17_FN_4_O: 288.33, found: 288.33.

*tert-Butyl (R)-4-(5-((4-fluorobenzyl)oxy)pyrimidin-2-yl)-3-methylpiperazine-1-carboxylate* (**pre-9k**): Yield: 41%; ^1^H-NMR (400 MHz, CDCl_3_): *δ* 8.10 (s, 2H), 7.37 (dd, *J* = 8.5, 5.4 Hz, 2H), 7.09–7.05 (m, 2H), 4.97 (s, 2H), 4.80–4.74 (m, 1H), 4.32–4.28 (m, 1H), 4.15–3.89 (m, 3H), 3.17–3.10 (m, 1H), 3.10–2.90 (m, 2H), 1.48 (s, 9H), 1.14 (d, *J* = 6.7 Hz, 3H).

*(R)-4-(5-((4-fluorobenzyl)oxy)pyrimidin-2-yl)-3-methylpiperazin-1-ium∙hydrochloride* (**9k**): Yield: 73%; ^1^H-NMR (400 MHz, CDCl_3_) δ 8.11 (s, 2H), 7.39–7.35 (m, 2H), 7.09–7.05 (m, 2H), 4.98 (s, 2H), 4.94–4.89 (m, 1H), 4.64–4.61 (m, 1H), 3.48–3.39 (m, 2H), 3.28–3.25 (m, 1H), 3.20–3.12 (m, 1H), 3.01–2.94 (m, 1H), 1.42 (d, *J* = 7.0 Hz, 3H). ^13^C-NMR (100 MHz, CD_3_OD) δ 162.8 (d, ^1^*J* = 245 Hz), 156.4, 146.4, 146.1, 131.9, 129.7 (d, ^3^*J* = 8 Hz), 115.8 (d, ^2^*J* = 21 Hz), 71.5, 47.2, 44.3, 43.3, 35.7, 14.0; LRMS-EI (m/z): [M**–**Cl]^+^ calcd for C_16_H_19_FN_4_O: 302.35, found: 302.35.

*tert-Butyl (R)-4-(5-((4-fluorophenethoxy)pyrimidin-2-yl)-3-methylpiperazine-1-carboxylate* (**pre-9l**): Yield: 39%; ^1^H-NMR (400MHz, CDCl_3_) *δ* 8.05 (s, 2H), 7.23–7.20 (m, 2H), 7.02–6.98 (m, 2H), 4.80–4.70 (m, 1H), 4.30–4.27 (m, 1H), 4.12 (t, *J* = 6.9 Hz, 2H), 3.98–3.87 (m, 2H), 3.17–3.09 (m, 1H), 3.03 (t, *J* = 6.8 Hz, 2H), 3.01–2.89 (m, 2H), 1.48 (s, 9H), 1.14 (d, *J* = 6.7 Hz, 3H).

*(R)-4-(5-((4-fluorophenethoxy)pyrimidin-2-yl)-3-methylpiperazin-1-ium∙hydrochloride* (**9l**): Yield: 85%; ^1^H-NMR (400 MHz, CDCl_3_): *δ* 8.06 (s, 2H), 7.22 (dd, *J* = 8.4, 5.5 Hz, 2H), 7.00 (t, *J =* 8.7 Hz, 2H), 4.87–4.85 (m, 1H), 4.46–4.42 (m, 1H), 4.12 (t, *J* = 6.8 Hz, 2H), 3.27–3.16 (m, 2H), 3.09–3.05 (m, 2H), 3.03 (t, *J* = 6.7 Hz, 2H), 2.90–2.84 (m, 1H), 1.37 (d, *J* = 7.0 Hz, 3H). ^13^C-NMR (100 MHz, CD3OD): *δ* 163.08 (d, ^1^*J* = 241 Hz), 157.7, 147.8, 146.9, 135.5 (d, ^4^*J* = 3 Hz), 131.7 (d, ^3^*J* = 8 Hz), 116.0 (d, ^2^*J* = 21 Hz), 71.7, 45.8, 44.8, 37.2, 35.9, 30.9, 30.7, 13.3; LRMS-EI (*m/z*): [M**–**Cl]^+^ calcd for C_17_H_21_FN_4_: 316.38, found: 316.38.

*tert-Butyl 4-(5-((3-fluorophenoxy)pyrimidin-2-yl)-1,4-diazepane-1-carboxylate* (**pre-9m**): Yield: 19%; ^1^H-NMR (400 MHz, CDCl_3_) *δ* 8.17 (s, 2H), 7.28–7.22 (m, 1H), 6.78–6.70 (m, 2H), 6.71 (m, 1H), 6.65–6.60 (m, 1H), 3.90–3.88 (m, 2H), 3.80–3.78 (m, 2H), 3.59–3.58 (m, 2H), 3.40–3.39 (m, 1H), 3.33–3.30 (m, 1H), 1.99–1.96 (m, 2H), 1.44 (s, 9H).

*4-(5-((3-fluorophenoxy)pyrimidin-2-yl)-1,4-diazepan-1-ium∙hydrochloride* (**9m**): Yield: 40%; ^1^H-NMR (400 MHz, CDCl_3_) *δ* 8.17 (s, 2H), 7.26–7.22 (m, 1H), 6.77–6.73 (m, 1H), 6.70–6.67 (m, 1H), 6.65–6.62 (m, 1H), 4.08–4.01 (s, 2H), 3.90 (t, *J* = 6.3 Hz, 2H), 3.36–3.27 (m, 2H), 3.16–3.15 (m, 2H), 2.16–2.14 (m, 2H). ^13^C-NMR (100 MHz, CD_3_OD) *δ* 165.0 (d, ^1^*J* = 244 Hz), 159.6, 152.3, 143.9, 132.1 (d, ^3^*J* = 10 Hz), 131.6 (d, ^3^*J* = 8 Hz), 113.3, 110.7 (d, ^2^*J* = 21 Hz), 105.2 (d, ^2^*J* = 25 Hz), 46.8, 46.5, 44.7, 26.8; LRMS-EI (*m/z*): [M**–**Cl]^+^ calcd for C_15_H_17_FN_4_O: 288.33, found: 288.33.

*tert-Butyl 4-(5-((3-fluorobenzyl)oxy)pyrimidin-2-yl)-1,4-diazepane-1-carboxylate* (**pre-9n**): Yield: 79%; ^1^H-NMR (400 MHz, CDCl_3_): *δ* 8.09 (s, 2H), 7.37–7.32 (m, 1H), 7.17–7.11 (m, 2H), 7.04–6.99 (m, 1H), 4.99 (s, 2H), 3.84–3.78 (m, 2H), 3.71–3.68 (m, 2H), 3.53 (s, 2H), 3.35 (t, *J* = 5.8 Hz, 1H), 3.25 (t, *J* = 5.9 Hz, 1H), 1.96–1.90 (m, 2H), 1.42 (s, 9H, a mixture of rotamers).

*4-(5-((3-fluorobenzyl)oxy)pyrimidin-2-yl)-1,4-diazepan-1-ium∙hydrochloride* (**9n**): Two-step yield: 53%; ^1^H-NMR (400 MHz, CDCl_3_) *δ* 8.10 (s, 2H), 7.38–7.32 (m, 1H), 7.17–7.11 (m, 2H), 7.05–7.01 (m, 1H), 5.01 (s, 2H), 3.92–3.90 (m, 2H), 3.81–3.77 (m, 2H), 3.76–3.71 (m, 2H), 3.58 (t, *J* = 6.0 Hz, 1H), 3.52 (t, *J* = 6.0 Hz, 1H), 2.07–1.97 (m, 2H). ^13^C-NMR (100 MHz, CD_3_OD) *δ* 164.4 (d, ^1^*J* = 243 Hz), 157.9, 147.8, 147.1, 140.9 (d, ^3^*J* = 7 Hz), 131.4 (d, ^3^*J* = 8 Hz), 124.3 (d, ^4^*J* = 3 Hz), 115.8 (d, ^2^*J* = 21 Hz), 115.3 (d, ^2^*J* = 22 Hz), 72.1, 72.1, 47.0, 46.6, 44.6, 26.9; LRMS-EI (*m/z*): [M**–**Cl]^+^ calcd for C_16_H_19_FN_4_O: 302.35, found: 302.35.

*tert-Butyl 4-(5-((4-fluorophenoxy)pyrimidin-2-yl)-1,4-diazepane-1-carboxylate* (**pre-9p**): Yield: 31%; ^1^H-NMR (400 MHz, CDCl_3_) *δ* 8.14 (s, 2H), 7.01–6.96 (m, 2H), 6.87–6.75 (m, 2H), 3.89–3.84 (m, 2H), 3.76–3.73 (m, 2H), 3.58–5.55 (m, 2H), 3.39 (t, *J* = 6.0 Hz, 1H), 3.31 (d, *J* = 6.0 Hz, 1H), 3.31 (t, *J* = 6.0 Hz, 1H), 1.98–1.92 (m, 2H), 1.44 (s, 9H).

*4-(5-((4-fluorophenoxy)pyrimidin-2-yl)-1,4-diazepan-1-ium∙hydrochloride* (**9p**): Yield: 34%; ^1^H-NMR (400 MHz, CDCl_3_) *δ* 8.14 (s, 2H), 7.00–6.96 (m, 2H), 6.90–6.87 (m, 2H), 4.10–4.01 (m, 2H), 3.98–3.88 (m, 1H), 3.38–3.30 (m, 2H), 3.27–3.20 (m, 2H), 2.20–2.15 (m, 2H). ^13^C-NMR (100 MHz, CD3OD) δ 163.9 (d, ^1^*J* = 244 Hz), 157.9, 147.9, 147.1, 134.1, 131.0 (d, ^3^*J* = 8 Hz), 116.3 (d, ^2^*J* = 21 Hz), 72.4, 47.1, 46.6, 46.5, 44.5, 26.8; LRMS-EI (*m/z*): [M**–**Cl]^+^ calcd for C_15_H_17_FN_4_O: 288.33, found: 288.33.

*4-(5-((3-fluorophenethoxy)pyrimidin-2-yl)-1,4-diazepan-1-ium∙hydrochloride* (**9o**): Yield: 6.5% (2 steps); ^1^H-NMR (400 MHz, CDCl_3_) *δ* 8.04 (s, 2H), 7.30–7.28 (m, 2H), 7.04–7.03 (m, 1H), 6.99–6.94 (m, 2H), 4.15 (t, *J* = 6.6 Hz, 2H), 3.91–3.89 (m, 2H), 3.79–3.77 (m, 2H), 3.75–3.72 (m, 2H), 3.57–3.55(m, 1H), 3,50–3.49 (m, 1H), 3.06 (t, *J* = 6.5 Hz, 2H), 2.05–1.98 (m, 2H). ^13^C-NMR (100 MHz, CD_3_OD) *δ* 147.4, 147.3, 147.2, 131.1, 131.0, 125.9, 125.9, 116.8, 116.6, 114.2, 114.0, 71.59, 47.7, 46.66, 46.5, 36.5, 25.9; LRMS-EI (*m/z*): [M**–**Cl]^+^ calcd for C_17_H_21_FN_4_O: 316.38, found: 316.38.

*4-(5-((4-fluorobenzyl)oxy)pyrimidin-2-yl)-1,4-diazepan-1-ium∙hydrochloride* (**9q**): Two-step yield: 7%; ^1^H-NMR (400 MHz, CDCl_3_) *δ* 8.11 (s, 2H), 7.37 (dd, *J* = 8.4, 5.4 Hz, 2H), 7.08 (t, *J* = 8.6 Hz, 2H), 4.97 (s, 2H), 4.07–4.03 (s, 2H), 3.89–3.86 (m, 2H), 3.35–3.29 (m, 2H), 3.20–3.17 (m, 2H), 2.23–2.22 (m, 2H). ^13^C-NMR (100 MHz, CD_3_OD) *δ* 165.4 (d, ^1^*J* = 243 Hz), 155.0, 147.0, 146.8, 133.5, 131.2 (d, ^3^*J* = 8 Hz), 116.4 (d, ^2^*J* = 21 Hz), 72.5, 47.2, 46.9, 46.6, 27.0; LRMS-EI (*m/z*): [M**–**Cl]^+^ calcd for C_16_H_19_FN_4_O: 302.35, found: 302.35.

*4-(5-((4-fluorophenethoxy)pyrimidin-2-yl)-1,4-diazepan-1-ium∙hydrochloride* (**9r**): Two-step yield: 3.5%; ^1^H-NMR (400 MHz, CDCl_3_) *δ* 8.04 (s, 2H), 7.22 (dd, *J* = 8.4, 5.5 Hz, 2H), 7.01 (t, *J* = 8.6 Hz, 2H), 4.12 (t, *J* = 6.8 Hz, 2H), 3.92–3.89 (m, 2H), 3.81–3.77 (m, 2H), 3.75–3.71 (m, 2H), 3.57 (t, *J* = 6.0 Hz, 1H), 3.51 (t, *J* = 6.0 Hz, 1H), 3.03 (t, *J* = 6.7 Hz, 2H), 2.07–1.96 (m, 2H). ^13^C-NMR (100 MHz, CD3OD) *δ* 156.4, 146.2, 145.8, 134.2, 130.3 (d, ^3^*J* = 8 Hz), 114.6 (d, ^2^*J* = 21 Hz), 70.5, 60.1, 45.6, 45.2, 43.2, 34.6, 29.5, 25.5; LRMS-EI (*m/z*): [M**–**Cl]^+^ calcd for C_17_H_21_FN_4_O: 316.38, found: 316.38.

### 3.3. Synthesis of 2,4-Disubstituted Pyrimidines **10a–j**

#### 3.3.1. General Procedure for Preparing Compound (R)-(+)-16

To a solution of 1-(3 or 4-fluorophenyl)ethanol (6.67 mmol) in *n*-hexane (22.2 mL) was added CAL-B (147 mg), vinyl acetate (3.34 mmol), and triethylamine (0.667 mmol). The reaction mixture was allowed to stir at the room temperature for 1 h. After completion of the reaction (monitored by TLC), the mixture was filtered and concentrated in vacuo. The resulting residue was purified by flash column chromatography on silica gel (EtOAc:*n*-hexane = 1:8) to afford acetate intermediate (315 mg) as a colorless oil. To a solution acetate (1.73 mmol) in MeOH (3.45 mL) was added 1M NaOH (2.59 mmol). The reaction mixture was allowed to stir at the room temperature for 1 h. After completion of the reaction (monitored by TLC), it was quenched with distilled water and extracted with EtOAc. The organic layers were dried over anhydrous MgSO_4_ and concentrated in vacuo. The resulting residue was purified by flash column chromatography on silica gel (EtOAc:*n*-hexane = 1:8) to afford alcohol (*R*)-(+)-**16.**

*(R)-1-(3-Fluorophenyl)ethan-1-ol* ((*R*)-(+)-**16a**): Yield: 17%; ^1^H-NMR (400 MHz, CDCl_3_) *δ* 7.32–7.26 (m, 1H), 7.12–7.07 (m, 2H), 6.97–6.92 (m, 1H), 4.87 (q, *J* = 6.4 Hz, 1H), 2.18 (s, 1H), 1.47 (d, *J* = 6.4 Hz, 3H). ^13^C-NMR (100 MHz, CDCl_3_) *δ* 163.0 (d, ^1^*J* = 244 Hz), 148.5 (d, ^3^*J* = 6 Hz), 130.0 (d, ^3^*J* = 8 Hz), 121.0 (d, ^4^*J* = 3 Hz), 114.2 (d, ^2^*J* = 21 Hz), 112.3 (d, ^2^*J* = 21 Hz), 69.8, 25.2; optical rotation for (*R*)-(+)-**16a**: [α]_D_^26^ + 43.7 ° (c 0.7, CHCl_3_).

*(R)-1-(4-Fluorophenyl)ethan-1-ol* ((*R*)-(+)-**16b**): Yield: 32%; ^1^H-NMR (400 MHz, CDCl_3_) *δ* 7.32–7.26 (m, 2H), 7.03–6.97 (m, 2H), 4.84 (q, *J* = 6.1 Hz, 1H), 2.34 (s, 1H), 1.44 (d, *J* = 6.4 Hz, 3H). ^13^C-NMR (100 MHz, CDCl_3_) *δ* 162.1 (d, ^1^*J* = 244 Hz), 141.6 (d, ^4^*J* = 3 Hz), 127.1 (d, ^3^*J* = 8 Hz), 115.2 (d, ^2^*J* = 21 Hz), 69.7, 25.3; optical rotation for (*R*)-(+)-**16b**: [α]_D_^27^ + 51.9 ° (c 0.5, CHCl_3_).

#### 3.3.2. General Procedure for Preparing Compound 17

A solution of sodium *tert*-butoxide (1.43 mmol) in toluene (7.10 mL) was treated with primary or secondary alcohol **16** (0.713 mmol) dropwise at 0 °C. After 5 min, 2,4-dichloro-5-fluoropyrimidine (0.713 mmol) was added to the mixture. The reaction mixture was allowed to stir at the room temperature for 1 h. After completion of the reaction (monitored by TLC), it was quenched with saturated aqueous NH_4_Cl, extracted with EtOAc, and washed with brine. The organic layers were dried over anhydrous MgSO_4_ and concentrated in vacuo. The resulting residue was purified by flash column chromatography on silica gel (EtOAc:*n*-hexane = 1:8) to afford pyrimidine **17**.

*(R)-2-Chloro-5-fluoro-4-(1-(3-fluorophenyl)ethoxy)pyrimidine* ((*R*)-**17a**): Yield: 72%; ^1^H-NMR (400 MHz, CDCl_3_) *δ* 8.19 (d, *J* = 2.2 Hz, 1H), 7.37–7.31 (m, 1H), 7.23 (d, *J* = 7.7 Hz, 1H), 7.18–7.15 (m, 1H), 7.03–6.99 (m, 1H), 6.30 (q, *J* = 6.5 Hz, 1H), 1.71 (d, *J* = 6.6 Hz, 3H). ^13^C-NMR (100 MHz, CDCl_3_) *δ* 162.9 (d, ^1^*J* = 245 Hz), 158.6 (d, ^2^*J* = 11 Hz), 153.2 (d, ^4^*J* = 5 Hz), 146.0 (d, ^1^*J* = 263 Hz), 144.4 (d, ^2^*J* = 20 Hz), 142.9 (d, ^3^*J* = 7 Hz), 130.3 (d, ^3^*J* = 8 Hz), 122.0 (d, ^4^*J* = 3 Hz), 115.3 (d, ^2^*J* = 21 Hz), 113.3 (d, ^2^*J* = 22 Hz), 75.6 (d, ^4^*J* = 2 Hz), 22.2; optical rotation for (*R*)-**6d**: [α]_D_^27^ +178.3 ° (c 0.7, CHCl_3_).

*(R)-2-Chloro-5-fluoro-4-(1-(4-fluorophenyl)ethoxy)pyrimidine* ((*R*)-**17b**): Yield: 68%; ^1^H-NMR (400 MHz, CDCl_3_) *δ* 8.16 (d, *J* = 2.2 Hz, 1H), 7.47–7.42 (m, 2H), 7.08–7.02 (m, 2H), 6.30 (q, *J* = 6.6 Hz, 1H), 1.71 (d, *J* = 6.6 Hz, 3H). ^13^C-NMR (100 MHz, CDCl_3_) *δ* 162.6 (d, ^1^*J* = 245 Hz), 158.7 (d, ^2^*J* = 11 Hz), 153.1 (d, ^4^*J* = 4 Hz), 146.0 (d, ^1^*J* = 263 Hz), 144.3 (d, ^2^*J* = 20 Hz), 136.1 (d, ^4^*J* = 4 Hz), 128.4 (d, ^3^*J* = 9 Hz), 115.6 (d, ^2^*J* = 22 Hz), 75.9, 22.2; optical rotation for (*R*)-**6e**: [α]_D_^27^ +197.3 ° (c 0.8, CHCl_3_).

2-Chloro-5-fluoro-4-(3-fluorophenoxy)pyrimidine (**19a**): Yield: 51%; ^1^H-NMR (400MHz, CDCl_3_) *δ* 8.38 (d, *J* = 2.0 Hz, 1H), 7.45–7.39 (m, 1H), 7.06–7.01 (m, 2H), 6.99–6.96 (m, 1H). ^13^C-NMR (100 MHz, CD_3_OD) *δ* 164.5 (d, ^1^*J* = 245 Hz), 159.8 (d, ^2^*J* = 11 Hz), 154.2 (d, ^4^*J* = 4 Hz), 153.7 (d, ^3^*J* = 11 Hz), 147.4 (d, ^1^*J* = 262 Hz), 147.4 (d, ^2^*J* = 20 Hz), 132.0 (d, ^3^*J* = 9 Hz), 118.5 (d, ^4^*J* = 3 Hz), 114.4 (d, ^2^*J* = 21 Hz), 110.5 (d, ^2^*J* = 25 Hz).

2-Chloro-5-fluoro-4-(4-fluorophenoxy)pyrimidine (**19b**): Yield: 52%; ^1^H-NMR (400MHz, CDCl_3_) *δ* 8.36 (d, *J* = 2.0 Hz, 1H), 7.19–7.11 (m, 4H). ^13^C-NMR (100 MHz, CD_3_OD) *δ* 162.0 (d, ^1^*J* = 242 Hz), 160.2 (d, ^2^*J* = 11 Hz), 154.2 (d, ^4^*J* = 5 Hz), 148.7 (d, ^4^*J* = 2 Hz), 147.2 (d, ^2^*J* = 20 Hz), 146.10, 124.3 (d, ^3^*J* = 9 Hz), 117.4 (d, ^2^*J* = 24 Hz).

#### 3.3.3. General Procedure for Preparing Compounds pre-10b–e and pre-10g–j

To a solution of pyrimidine **17** (0.154 mmol) in toluene (0.300 mL) was added cyclic amine (0.231 mmol) and triethylamine (0.231 mmol). The reaction mixture was allowed to stir at 90 °C for 12 h. After completion of the reaction (monitored by TLC), it was quenched with saturated aqueous NH_4_Cl, extracted with EtOAc, and washed with brine. The organic layers were dried over anhydrous MgSO_4_ and concentrated in vacuo. The resulting residue was purified by flash column chromatography on silica gel (EtOAc/*n*-hexane = 1:3) to afford carboxylate **pre-10b–e** and **pre-10g–j**.

*tert-Butyl 7-(5-fluoro-4-((R)-1-(3-fluorophenyl)ethoxy)pyrimidin-2-yl)-2,7-diazaspiro[4.4]nonane-2- carboxylate* (**pre-10b**): Yield: 92%; ^1^H-NMR (400 MHz, CDCl_3_, a mixture of rotamers) *δ* 7.94 (s, 1H), 7.31–7.26 (m, 1H), 7.17 (d, *J* = 7.6 Hz, 1H), 7.11 (d, *J* = 9.6 Hz, 1H) 6.95 (t, *J* = 8.3 Hz, 1H), 6.15–6.10 (m, 1H), 3.59–3.23 (m, 8H), 1.94–1.83 (m, 4H), 1.66 (d, *J* = 6.6 Hz, 3H), 1.45 (s, 9H). ^13^C-NMR (100 MHz, CDCl_3_, a mixture of rotamers) *δ* 162.9 (d, ^1^*J* = 244 Hz), 157.1 (d, ^2^*J* = 11 Hz), 156.0, 154.6, 145.0 (d, ^3^*J* = 7 Hz), 143.4 (d, ^2^*J* = 20 Hz), 140.0 (d, ^1^*J* = 245 Hz), 130.0 (d, ^3^*J* = 8 Hz), 121.6, 114.6 (d, ^2^*J* = 21 Hz), 113.1 (d, ^2^*J* = 22 Hz), 79.4, 73.7, 55.9, 55.3, 54.7, 54.6, 48.6, 47.8, 46.0, 45.2, 35.3, 34.9, 34.5, 28.5, 22.7.

*tert-Butyl (3aR,6aS)-5-(5-fluoro-4-((R)-1-(3-fluorophenyl)ethoxy)pyrimidin-2-yl) hexahydropyrrolo [3,4-c]pyrrole-2(1H)-carboxylate* (**pre-10c**): Yield: 82%; ^1^H-NMR (400 MHz, CDCl_3_, a mixture of rotamers) δ 7.94 (d, *J* = 3.1 Hz, 1H), 7.32–7.28 (m, 1H), 7.17 (d, *J* = 7.7 Hz, 1H), 7.11 (dd, *J* = 2.0, 9.7 Hz, 1H), 6.95 (td, *J* = 2.0, 8.4 Hz, 1H), 6.14 (q, *J* = 6.6 Hz, 1H), 3.69–3.59 (m, 4H), 3.40–3.21 (m, 4H), 2.94 (bs, 2H), 1.66 (d, *J* = 6.6 Hz, 3H), 1.47 (s, 9H). ^13^C-NMR (100 MHz, CDCl_3_, a mixture of rotamers) δ 162.9 (d, ^1^*J* = 244 Hz), 157.1 (d, ^2^*J* = 11 Hz), 156.0 (d, ^4^*J* = 3 Hz), 154.5, 145.0 (d, ^3^*J* = 10 Hz), 143.4 (d, ^2^*J* = 20 Hz), 140.1 (d, ^1^*J* = 246 Hz), 130.0 (d, ^3^*J* = 8 Hz), 121.6, 114.6 (d, ^2^*J* = 21 Hz), 113.0 (d, ^2^*J* = 21 Hz), 79.5, 73.6, 50.8, 50.1, 49.8, 42.2, 41.2, 28.5, 22.7.

*tert-Butyl (R)-3-((5-fluoro-4-((R)-1-(3-fluorophenyl)ethoxy)pyrimidin-2-yl)aminopyrrolidine-1- carboxylate* (**pre-10d**): Yield: 68%; ^1^H-NMR (400 MHz, CDCl_3_, a mixture of rotamers) *δ* 7.89 (s, 1H), 7.31–7.28 (m, 1H), 7.14 (d, *J* = 7.7 Hz, 1H), 7.08 (d, *J* = 9.6 Hz, 1H) 6.94 (t, *J* = 7.9 Hz, 1H), 6.08 (d, *J* = 4.4 Hz, 1H), 5.12 (bs, 1H), 4.24–4.16 (m, 1H), 3.71–3.65 (m, 1H), 3.43–3.37 (m, 2H), 3.28–3.12 (m, 1H), 2.01–1.98 (m, 1 H), 1.65 (d, *J* = 6.6 Hz, 3H), 1.45 (s, 9H). ^13^C-NMR (100 MHz, CDCl_3_, a mixture of rotamers) *δ* 162.9 (d, ^1^*J* = 245 Hz), 157.6 (d, ^2^*J* = 11 Hz), 157.3, 154.6, 144.7 (d, ^3^*J* = 6 Hz), 143.7 (d, ^2^*J* = 20 Hz), 140.6 (d, ^1^*J* = 248 Hz), 130.1 (d, ^3^*J* = 8 Hz), 121.3, 114.6 (d, ^2^*J* = 22 Hz), 112.8 (d, ^2^*J* = 22 Hz), 79.5, 73.9, 51.8, 51.7, 51.4, 51.0, 44.1, 43.7, 31.8, 31.1, 28.5, 22.8.

*tert-Butyl (S)-3-((5-fluoro-4-((R)-1-(3-fluorophenyl)ethoxy)pyrimidin-2-yl)aminopyrrolidine-1- carboxylate* (**pre-10e**): Yield: 52%; ^1^H-NMR (400 MHz, CDCl_3_, a mixture of rotamers) *δ* 7.90 (d, *J* = 2.9 Hz, 1H), 7.33–7.26 (m, 1H), 7.15 (d, *J* = 7.7 Hz, 1H), 7.10 (d, *J* = 9.6 Hz, 1H) 6.98–6.94 (m, 1H), 6.11 (q, *J* = 6.5 Hz, 1H), 5.12 (bs, 1H), 4.28 (bs, 1H), 3.47–3.42 (m, 3H), 3.21–3.04 (m, 1H), 2.20–2.12 (m, 1H), 1.86 (bs, 1H), 1.65 (d, *J* = 6.6 Hz, 3H), 1.45 (s, 9H). ^13^C-NMR (100 MHz, CDCl_3_, a mixture of rotamers) *δ* 162.9 (d, ^1^*J* = 245 Hz), 157.6 (d, ^2^*J* = 11 Hz), 157.2, 154.6, 143.6 (d, ^2^*J* = 20 Hz), 143.3 (d, ^1^*J* = 271 Hz), 139.4, 130.1 (d, ^3^*J* = 8 Hz), 121.4, 114.7 (d, ^2^*J* = 21 Hz), 112.8 (d, ^2^*J* = 22 Hz), 79.5, 73.8, 51.9, 51.6, 51.4, 51.0, 44.1, 43.8, 31.9, 31.1, 28.5, 22.8.

*tert-Butyl 7-(5-fluoro-4-((R)-1-(4-fluorophenyl)ethoxy)pyrimidin-2-yl)-2,7-diazaspiro[4.4]nonane-2- carboxylate* (**pre-10g**): Yield: 98%; ^1^H-NMR (400 MHz, CDCl_3_, a mixture of rotamers) *δ* 7.93 (s, 1H), 7.40–7.37 (m, 2H), 7.03–6.99 (m, 2H), 6.18–6.13 (m, 1H), 3.61–3.22 (m, 8H), 1.95–1.82 (m, 4H), 1.66 (d, *J* = 6.6 Hz, 3H), 1.45 (s, 9H). ^13^C-NMR (100 MHz, CDCl_3_, a mixture of rotamers) *δ* 162.2 (d, ^1^*J* = 244 Hz), 157.2 (d, ^2^*J* = 11 Hz), 156.0, 154.6, 143.3 (d, ^2^*J* = 20 Hz), 140.1 (d, ^1^*J* = 246 Hz), 138.0, 127.9 (d, ^3^*J* = 8 Hz), 115.3 (d, ^2^*J* = 21 Hz), 79.4, 73.6, 55.9, 55.4, 54.8, 54.6, 48.6, 47.8, 46.0, 45.2, 44.9, 35.3, 34.9, 34.5, 28.5, 22.8.

*tert-Butyl (3aR,6aS)-5-(5-fluoro-4-((R)-1-(4-fluorophenyl)ethoxy)pyrimidin-2-yl)hexahydropyrrolo [3,4-c]pyrrole-2(1H)-carboxylate* (**pre-10h**): Yield: 93%; ^1^H-NMR (400 MHz, CDCl_3_, a mixture of rotamers) *δ* 7.92 (d, *J* = 3.1 Hz, 1H), 7.40–7.36 (m, 2H), 7.00 (t, *J* = 8.6 Hz, 2H), 6.15 (d, *J* = 5.7 Hz, 1H), 3.73–3.60 (m, 4H), 3.41–3.21 (m, 4H), 2.91 (bs, 2H), 1.64 (d, *J* = 6.6 Hz, 3H), 1.44 (s, 9H). ^13^C-NMR (100 MHz, CDCl_3_, a mixture of rotamers) *δ* 162.2 (d, ^1^*J* = 245 Hz), 157.2 (d, ^2^*J* = 11 Hz), 156.0 (d, ^4^*J* = 3 Hz), 154.5, 143.3 (d, ^2^*J* = 20 Hz), 140.1 (d, ^1^*J* = 245 Hz), 138.0, 127.8 (d, ^3^*J* = 8 Hz), 115.3 (d, ^2^*J* = 21 Hz), 79.5, 73.6, 50.8, 50.1, 49.8, 42.2, 41.2, 28.5, 22.8.

*tert-Butyl (R)-3-((5-fluoro-4-((R)-1-(4-fluorophenyl)ethoxy)pyrimidin-2-yl)amino)pyrrolidine-1- carboxylate* (**pre-10i**): Yield: 52%; ^1^H-NMR (400 MHz, CDCl_3_, a mixture of rotamers) *δ* 7.88 (d, *J* = 2.0 Hz, 1H), 7.38–7.34 (m, 2H), 7.01 (t, *J* = 8.5 Hz, 2H), 6.14–6.09 (m, 1H), 5.13 (bs, 1H), 4.26–4.19 (m, 1H), 3.69–3.66 (m, 1H), 3.40 (bs, 2H), 3.29–3.16 (m, 1H), 2.09–2.01 (m, 1H), 1.74–1.73 (m, 1H), 1.64 (d, *J* = 6.6 Hz, 3H), 1.46 (s, 9H). ^13^C-NMR (100 MHz, CDCl_3_, a mixture of rotamers) *δ* 162.2 (d, ^1^*J* = 245 Hz), 157.7 (d, ^2^*J* = 11 Hz), 157.3, 154.6, 143.5 (d, ^2^*J* = 21 Hz), 140.6 (d, ^1^*J* = 246 Hz), 137.8 (d, ^2^*J* = 20 Hz), 127.6 (d, ^3^*J* = 12 Hz), 115.4 (d, ^2^*J* = 21 Hz), 79.5, 73.9, 51.8, 51.7, 51.5, 51.0, 44.1, 43.8, 31.8, 31.1, 28.5, 22.8.

*tert-Butyl (S)-3-((5-fluoro-4-((R)-1-(4-fluorophenyl)ethoxy)pyrimidin-2-yl)aminopyrrolidine-1- carboxylate* (**pre-10j**): Yield: 44%; ^1^H-NMR (400 MHz, CDCl_3_, a mixture of rotamers) *δ* 7.89 (d, *J* = 3.0 Hz, 1H), 7.39–7.36 (m, 2H), 7.03 (t, *J* = 8.6 Hz, 2H), 6.14 (q, *J* = 6.5 Hz, 1H), 4.98 (bs, 1H), 4.31 (m, 1H), 3.62–3.44 (m, 3H), 3.24–3.06 (m, 1H), 2.28-2.13 (m, 1H), 1.86 (bs, 1H), 1.64 (d, *J* = 6.6 Hz, 3H), 1.45 (s, 9H). ^13^C-NMR (100 MHz, CDCl_3_, a mixture of rotamers) *δ* 162.3 (d, ^1^*J* = 244 Hz), 157.7 (d, ^2^*J* = 11 Hz), 157.2, 154.6, 143.5 (d, ^2^*J* = 19 Hz), 140.8 (d, ^1^*J* = 247 Hz), 137.7, 127.7, 130.1, 115.4 (d, ^2^*J* = 21 Hz), 79.5, 73.8, 52.0, 51.7, 51.5, 51.0, 44.1, 43.8, 31.9, 31.1, 29.8, 28.5, 22.8.

*tert-Butyl (R)-4-(5-fluoro-4-(3-fluorophenoxy)pyrimidin-2-yl)-3-methylpiperazine-1-carboxylate* (**pre-20a**): Yield: 74%; ^1^H-NMR (400MHz, CDCl_3_) *δ* 8.13 (d, *J* = 2.7 Hz, 1H), 7.39–7.33 (m, 1H), 6.99–6.94 (m, 3H), 4.44 (m, 1H), 4.05–3.80 (m, 3H), 3.06–3.2.99 (m, 2H), 2.99–2.82 (m, 1H), 1.45 (s, 9H), 1.04 (d, *J* = 6.6 Hz, 3H).

*tert-Butyl (R)-4-(5-fluoro-4-(4-fluorophenoxy)pyrimidin-2-yl)-3-methylpiperazine-1-carboxylate* (**pre-20b**): Yield: 61%; ^1^H-NMR (400MHz, CDCl_3_) *δ* 8.11 (d, *J* = 2.7 Hz, 1H), 7.15–7.12 (m, 2H), 7.11–7.06 (m, 2H), 4.50–4.30 (m, 1H), 4.03–3.80 (m, 3H), 3.04–2.97 (m, 2H), 2.97–2.81 (m, 1H), 1.45 (s, 9H), 1.03 (d, *J* = 6.6 Hz, 3H).

#### 3.3.4. General Procedure for Preparing Compounds 10a and 10f

To a solution of pyrimidine **17** (0.115 mmol) in toluene (0.250 mL) was added 1,4-diazepane (0.230 mmol) and triethylamine (0.230 mmol). The reaction mixture was allowed to stir at 90 °C for 12 h. After completion of the reaction (monitored by TLC), it was quenched with saturated aqueous NH_4_Cl, extracted with EtOAc, and washed with brine. The organic layers were dried over anhydrous MgSO_4_ and concentrated in vacuo. The resulting residue was purified by flash column chromatography on silica gel (DCM/MeOH = 10:1) to afford 2,4-disubstituted pyrimidine **10a** and **10f**.

*(R)-1-(5-fluoro-4-(1-(3-fluorophenyl)ethoxy)pyrimidin-2-yl)-1,4-diazepane* (**10a**): Yield: 68%; ^1^H-NMR (400 MHz, CD_3_OD) *δ* 8.06 (d, *J* = 3.2 Hz, 1H), 7.23 (d, *J* = 7.7 Hz, 1H), 7.15 (d, *J* = 9.8 Hz, 1H), 7.00 (td, *J* = 2.1, 8.5 Hz, 1H), 6.11 (q, *J* = 6.5 Hz, 1H), 3.90–3.77 (m, 4H), 3.26–3.03 (m, 4H), 2.07–1.94 (m, 2H), 1.66 (d, *J* = 6.6 Hz, 3H). ^13^C-NMR (100 MHz, CD_3_OD) *δ* 163.0 (d, ^1^*J* = 244 Hz), 157.7 (d, ^2^*J* = 11 Hz), 155.9, 145.5 (d, ^3^*J* = 7 Hz), 142.5 (d, ^2^*J* = 21 Hz), 140.3 (d, ^1^*J* = 246 Hz), 130.3 (d, ^3^*J* = 8 Hz), 121.1 (d, ^4^*J* = 3 Hz), 114.1 (d, ^2^*J* = 21 Hz), 112.0 (d, ^2^*J* = 22 Hz), 74.7, 45.5, 45.3, 45.0, 43.3, 25.3, 22.0. LRMS-EI (*m/z*): [M+H]^+^ calcd for C_17_H_21_F_2_N_4_O: 335.17, found: 335.10.

*(R)-1-(5-fluoro-4-(1-(4-fluorophenyl)ethoxy)pyrimidin-2-yl)-1,4-diazepane* (**10f**): Yield: 79%; ^1^H-NMR (400 MHz, CD_3_OD) *δ* 8.05 (d, *J* = 3.2 Hz, 1H), 7.45 (dd, *J* = 5.4, 8.4 Hz, 1H), 7.11–7.07 (m, 2H), 6.16 (q, *J* = 6.5 Hz, 1H), 4.10–3.72 (m, 4H), 3.42–3.15 (m, 4H), 2.21–1.98 (m, 2H), 1.66 (d, *J* = 6.5 Hz, 3H). ^13^C-NMR (100 MHz, CD_3_OD) *δ* 162.3 (d, ^1^*J* = 243 Hz), 158.0 (d, ^2^*J* = 12 Hz), 155.60, 141.7 (d, ^2^*J* = 20 Hz), 140.3 (d, ^1^*J* = 246 Hz), 138.3 (d, ^4^*J* = 3 Hz), 127.4 (d, ^3^*J* = 8 Hz), 115.0 (d, ^2^*J* = 22 Hz), 75.0, 45.6, 45.4, 45.0, 43.4, 25.3, 22.1. LRMS-EI (*m/z*): [M+H]^+^ calcd for C_17_H_21_F_2_N_4_O: 335.17, found: 335.10.

#### 3.3.5. General Procedure for Preparing Compounds 10b–e and 10g–j

To a solution of carboxylate **17** (0.144 mmol) in CH_3_CN (1.45 mL) was added 4M HCl in dioxane (1.44 mmol) at 0 °C. The reaction mixture was allowed to stir at the same temperature for 1 h. After completion of the reaction (monitored by TLC), the mixture was diluted with saturated aqueous NaHCO_3_ and extracted with EtOAc. The organic layers were dried over anhydrous MgSO4 and concentrated in vacuo. The resulting residue was purified by flash column chromatography on silica gel (DCM/MeOH = 10:1) to afford compound **10**.

*2-(5-fluoro-4-((R)-1-(3-fluorophenyl)ethoxy)pyrimidin-2-yl)-2,7-diazaspiro[4.4]nonane* (**10b**): Yield: 66%; ^1^H-NMR (400 MHz, CD_3_OD, a mixture of rotamers) *δ* 8.00 (d, *J* = 3.4 Hz, 1H), 7.35–7.30 (m, 1H), 7.21 (d, *J* = 7.7 Hz, 1H), 7.13 (d, *J* = 9.8 Hz, 1H), 6.97 (td, *J* = 1.7, 8.4 Hz, 1 H), 6.23–6.16 (m, 1H), 3.59–3.37 (m, 6H), 3.30–3.23 (m, 2H), 2.11–1.98 (m, 4H), 1.64 (d, *J* = 6.6 Hz, 3H). ^13^C-NMR (100 MHz, CD_3_OD, a mixture of rotamers) *δ* 162.9 (d, ^1^*J* = 243 Hz), 158.3 (d, ^2^*J* = 12 Hz), 154.39, 144.7 (d, ^3^*J* = 9 Hz), 140.0 (d, ^2^*J* = 21 Hz), 139.8 (d, ^1^*J* = 246 Hz), 130.1 (d, ^3^*J* = 8 Hz), 121.5 (d, ^4^*J* = 3 Hz), 114.3 (d, ^2^*J* = 21 Hz), 112.5 (d, ^2^*J* = 22 Hz), 75.0, 74.9, 55.3, 55.5, 46.0, 44.8, 44.7, 34.3, 34.2, 33.8, 33.7, 21.5, 21.5. LRMS-EI (*m/z*): [M+H]^+^ calcd for C_19_H_23_F_2_N_4_O: 361.18, found: 361.10.

*(3aR,6aS)-2-(5-fluoro-4-((R)-1-(3-fluorophenyl)ethoxy)pyrimidin-2-yl)octahydropyrrolo[3,4-c]pyrrole* (**10c**): Yield: 92%; ^1^H-NMR (400 MHz, CD_3_OD, a mixture of rotamers) *δ* 8.26 (d, *J* = 4.4 Hz, 1H), 7.44–7.38 (m, 1H), 7.32 (d, *J* = 7.7 Hz, 1H), 7.24 (dd, *J* = 1.9, 9.7 Hz, 1H), 7.06 (td, *J* = 2.3, 8.4 Hz, 1H), 3.91 (bs, 1H), 3.80 (bs, 2H), 3.68–3.58 (m, 3H), 3.37–3.28 (m, 4H), 1.75 (d, *J* = 6.5 Hz, 3H). ^13^C-NMR (100 MHz, CD_3_OD, a mixture of rotamers) *δ* 162.9 (d, ^1^*J* = 244 Hz), 157.8 (d, ^2^*J* = 12 Hz), 152.12, 149.9, 143.9 (d, ^3^*J* = 7 Hz), 139.6 (d, ^1^*J* = 248 Hz), 135.6 (d, ^2^*J* = 26 Hz), 130.3 (d, ^3^*J* = 8 Hz), 121.7 (d, ^4^*J* = 3 Hz), 114.6 (d, ^2^*J* = 21 Hz), 112.6 (d, ^2^*J* = 22 Hz), 79.6, 50.9, 50.8, 50.7, 49.6, 49.4, 41.6, 41.5, 21.3. LRMS-EI (*m/z*): [M+H]^+^ calcd for C_18_H_21_F_2_N_4_O: 347.17, found: 347.05.

*5-fluoro-4-((R)-1-(3-fluorophenyl)ethoxy)-N-((R)-pyrrolidin-3-yl)pyrimidin-2-amine* (**10d**): Yield: 71%; ^1^H-NMR (400 MHz, CD_3_OD, a mixture of rotamers) *δ* 7.97 (d, *J* = 3.1 Hz, 1H), 7.36–7.29 (m, 1H), 7.18 (d, *J* = 7.7 Hz, 1H), 7.10 (d, *J* = 9.8 Hz, 1H), 6.96 (td, *J* = 2.3, 8.5 Hz, 1H), 6.17 (q, *J* = 6.5 Hz, 1H), 4.38–4.33 (m, 1H), 3.49–3.30 (m, 1H), 3.26–3.25 (m, 3H), 2.30–2.14 (m, 1H), 2.09–1.93 (m, 1 H), 1.61 (d, *J* = 6.6 Hz, 3H). ^13^C-NMR (100 MHz, CD_3_OD, a mixture of rotamers) *δ* 163.0 (d, ^1^*J* = 243 Hz), 158.0 (d, ^2^*J* = 12 Hz), 156.9, 144.9 (d, ^3^*J* = 7 Hz), 142.2 (d, ^2^*J* = 17 Hz), 140.6 (d, ^1^*J* = 247 Hz), 130.2 (d, ^3^*J* = 8 Hz), 121.2 (d, ^4^*J* = 3 Hz), 114.1 (d, ^2^*J* = 21 Hz), 112.1 (d, ^2^*J* = 22 Hz), 74.1, 51.0, 50.3, 50.1, 44.1, 29.7, 29.5, 21.74, 21.66. LRMS-EI (*m/z*): [M+H]^+^ calcd for C_16_H_19_F_2_N_4_O: 321.15, found: 321.05.

*5-fluoro-4-((R)-1-(3-fluorophenyl)ethoxy)-N-((S)-pyrrolidin-3-yl)pyrimidin-2-amine* (**10e**): Yield: 42%; ^1^H-NMR (400 MHz, CD_3_OD, a mixture of rotamers) *δ* 7.93–7.92 (m, 1H), 7.34–7.28 (m, 1H), 7.17 (d, *J* = 7.7 Hz, 1H), 7.09 (d, *J* = 9.9 Hz, 1H), 6.97–6.92 (m, 1H), 6.15 (q, *J* = 6.5 Hz, 1H), 4.34–4.29 (m, 1H), 3.49–3.38 (m, 2H), 3.35–3.25 (m, 2H), 2.98–2.11 (m, 1H), 2.10–1.89 (m, 1 H), 1.59 (d, *J* = 6.6 Hz, 3H). ^13^C-NMR (100 MHz, CD_3_OD, a mixture of rotamers) *δ* 162.9 (d, ^1^*J* = 243 Hz), 157.7 (d, ^2^*J* = 11 Hz), 157.3 (d, ^4^*J* = 3 Hz), 145.0 (d, ^3^*J* = 7 Hz), 143.0 (d, ^2^*J* = 20 Hz), 140.7 (d, ^1^*J* = 246 Hz), 130.1 (d, ^3^*J* = 8 Hz), 121.3 (d, ^4^*J* = 3 Hz), 114.1 (d, ^2^*J* = 21 Hz), 112.1 (d, ^2^*J* = 22 Hz), 73.8, 51.0, 50.2, 44.1, 29.7, 29.6, 21.8, 21.7. LRMS-EI (*m/z*): [M+H]^+^ calcd for C_16_H_19_F_2_N_4_O: 321.15, found: 321.05.

*2-(5-fluoro-4-((R)-1-(4-fluorophenyl)ethoxy)pyrimidin-2-yl)-2,7-diazaspiro[4.4]nonane* (**10g**): Yield: 58%; ^1^H-NMR (400 MHz, CD_3_OD, a mixture of rotamers) *δ* 7.90 (d, *J* = 3.3 Hz, 1H), 7.44–7.40 (m, 2H), 7.03 (t, *J* = 8.8 Hz, 2H), 6.22–6.15 (m, 1H), 3.59–3.35 (m, 6H), 3.30–3.19 (m, 2H), 2.09–1.96 (m, 4H), 1.61 (d, *J* = 6.6 Hz, 3H). ^13^C-NMR (100 MHz, CD_3_OD, a mixture of rotamers) *δ* 162.3 (d, ^1^*J* = 243 Hz), 157.5 (d, ^2^*J* = 11 Hz), 155.9, 142.7 (d, ^2^*J* = 20 Hz), 140.0 (d, ^1^*J* = 245 Hz), 138.3 (d, ^4^*J* = 7 Hz), 127.8 (d, ^3^*J* = 8 Hz), 114.8 (d, ^2^*J* = 21 Hz), 74.03, 73.97, 55.2, 52.7, 45.7, 44.8, 34.4, 34.3, 33.9, 33.8, 21.7, 21.6. LRMS-EI (*m/z*): [M+H]^+^ calcd for C_19_H_23_F_2_N_4_O: 361.18, found: 361.10.

*(3aR,6aS)-2-(5-fluoro-4-((R)-1-(4-fluorophenyl)ethoxy)pyrimidin-2-yl)octahydropyrrolo[3,4-c]pyrrole* (**10h**): Yield: 53%; ^1^H-NMR (400 MHz, CD_3_OD, a mixture of rotamers) *δ* 7.96 (d, *J* = 2.8 Hz, 1H), 7.27 (bs, 2H), 6.86 (t, *J* = 8.0 Hz, 1H), 6.11 (bs, 1H), 3.66–3.54 (m, 3H), 3.43–3.36 (m, 3H), 3.07–3.03 (m, 4H), 1.48 (d, *J* = 3.0 Hz, 3H). ^13^C-NMR (100 MHz, CD_3_OD, a mixture of rotamers) *δ* 162.8 (d, ^1^*J* = 244 Hz), 161.1 (d, ^2^*J* = 12 Hz), 150.0, 139.3 (d, ^1^*J* = 250 Hz), 136.4 (d, ^4^*J* = 3 Hz), 131.6 (d, ^2^*J* = 20 Hz), 128.4 (d, ^3^*J* = 8 Hz), 115.2 (d, ^2^*J* = 21 Hz), 78.2, 51.4, 51.2, 49.5, 41.7, 41.6, 21.2. LRMS-EI (*m/z*): [M+H]^+^ calcd for C_18_H_21_F_2_N_4_O: 347.17, found: 347.05.

*5-fluoro-4-((R)-1-(4-fluorophenyl)ethoxy)-N-((R)-pyrrolidin-3-yl)pyrimidin-2-amine* (**10i**): Yield: 51%; 1H-NMR (400 MHz, CD_3_OD, a mixture of rotamers) *δ* 7.93 (d, *J* = 3.3 Hz, 1H), 7.49–7.45 (m, 2H), 7.10 (t, *J* = 8.7 Hz, 2H), 6.25–6.22 (m, 1H), 4.31–4.30 (m, 1H), 3.56–3.40 (m, 3H), 3.15–3.06 (m, 1H), 2.23–2.16 (m, 1H), 1.94–1.90 (m, 1 H), 1.66 (d, *J* = 6.6 Hz, 3H). LRMS-EI (*m/z*): [M+H]^+^ calcd for C_16_H_19_F_2_N_4_O: 321.15, found: 321.00.

*5-Fluoro-4-((R)-1-(4-fluorophenyl)ethoxy)-N-((S)-pyrrolidin-3-yl)pyrimidin-2-amine* (**10j**): Yield: 27%; ^1^H-NMR (400 MHz, CD_3_OD, a mixture of rotamers) *δ* 7.92–7.91 (m, 1H), 7.42–7.39 (m, 2H), 7.06–7.01 (m, 2H), 6.18 (td, *J* = 4.8, 6.5 Hz, 1H), 4.39–4.30 (m, 1H), 3.50–3.39 (m, 2H), 3.36–3.28 (m, 2H), 2.39–2.14 (m, 1H), 2.10–1.90 (m, 1 H), 1.59 (d, *J* = 6.6 Hz, 3H). ^13^C-NMR (100 MHz, CD_3_OD, a mixture of rotamers) *δ* 162.3 (d, ^1^*J* = 243 Hz), 157.8 (d, ^2^*J* = 11 Hz), 157.3 (d, ^4^*J* = 3 Hz), 143.0 (d, ^2^*J* = 20 Hz), 140.8 (d, ^1^*J* = 233 Hz), 138.1, 127.7 (d, ^3^*J* = 8 Hz), 114.9 (d, ^2^*J* = 22 Hz), 73.9, 51.0, 50.4, 50.3, 44.2, 29.7, 29.6, 21.8, 21.7. LRMS-EI (*m/z*): [M+H]^+^ calcd for C_16_H_19_F_2_N_4_O: 321.15, found: 321.05.

*(R)-4-(5-Fluoro-4-(3-fluorophenoxy)pyrimidin-2-yl)-3-methylpiperazin-1-ium·hydrochloride* (**20a**): Yield: 58%; ^1^H-NMR (400MHz, CDCl_3_) *δ* 8.13 (s, 1H), 7.37–7.31 (m, 1H), 7.00–6.93 (m, 3H), 4.39–4.38 (m, 1H), 4.07–4.03 (m, 1H), 2.98–2.86 (m, 3H), 2.80–2.77 (m, 1H), 2.70–2.62 (m, 1H), 1.12 (d, *J* = 6.8 Hz, 3H). ^13^C-NMR (100 MHz, CD_3_OD) *δ* 164.4 (d, ^1^*J* = 245 Hz), 158.7 (d, ^2^*J* = 11 Hz), 157.2 (d, ^3^*J* = 8 Hz), 154.4 (d, ^3^*J* = 11 Hz), 146.7 (d, ^3^*J* = 20 Hz), 141.4 (d, ^1^*J* = 248 Hz), 131.7 (d, ^3^*J* = 9 Hz), 118.8 (d, ^4^*J* = 3 Hz), 113.7 (d, ^2^*J* = 21 Hz), 110.6 (d, ^2^*J* = 24 Hz), 45.7, 44.3, 36.6, 13.5. LRMS-EI (*m/z*): [M-Cl]^+^ calcd for C_15_H_17_F_2_N_4_O: 306.32, found: 306.32.

*(R)-4-(5-Fluoro-4-(4-fluorophenoxy)pyrimidin-2-yl)-3-methylpiperazin-1-ium·hydrochloride* (**20b**): Yield: 65%; ^1^H-NMR (400MHz, CDCl_3_): *δ* 8.08 (d, *J* = 2.6 Hz, 1H), 7.13 (dd, *J* = 9.0, 4.5 Hz, 2H), 7.09–7.03 (m, 2H), 4.50–4.30 (s, 1H), 4.03–4.00 (m, 1H), 2.96–2.83 (m, 3H), 2.77–2.74 (m, 1H), 2.68–2.60 (m, 1H), 1.09 (d, *J* = 6.6 Hz, 3H). ^13^C-NMR (100 MHz, CD_3_OD): *δ* 161.6 (d, ^1^*J* = 241 Hz), 159.2 (d, ^2^*J* = 11 Hz), 149.3 (d, ^4^*J* = 22 Hz), 146.4 (d, ^2^*J* = 20 Hz), 141.5 (d, ^1^*J* = 248 Hz), 124.5 (d, ^3^*J* = 8 Hz), 117.1 (d, ^2^*J* = 23 Hz), 48.0, 45.6, 44.2, 36.5, 13.5. LRMS-EI (*m/z*): [M-Cl]^+^ calcd for C_15_H_17_F_2_N_4_O: 306.32, found: 306.32

### 3.4. Molecular Docking Study

Discovery studio 2019 software were used for modeling study [34]. Crystal structures of 5-HT_2B_ (PDB ID = 4IB4) and 5-HT_2C_ (PDB ID= 6BQH) proteins were downloaded from protein data bank. Ligand **10a** and **8** were prepared at 7.4 pH. Proteins and ligands were prepared and minimized using CHARm forcefield. Binding sites were created over co-crystal ligands binding position. Ligands were docked using CDOCKER scoring function. Twenty binding poses were generated for each ligands. Obtained docked poses were analyzed based on docking scores and binding site interactions. Images were rendered using PyMOL software (www.pymol.org).

### 3.5. In Vitro Assay

#### 3.5.1. Serotonin Receptor Cell-Based Functional Assays

The synthesized compounds were tested in agonist mode with the 5-HT_2C_ receptor assay using recombinant human HEK-293 cells serviced by either Eurofins Cerep or DGMIF (Daegu-Gyeongbuk Medical Innovation Foundation). All the experiments were duplicated. The detailed assay protocols refer to the literature procedure [35].

#### 3.5.2. Serotonin Receptor Binding Affinity Assays

Eleven dilutions (5 x assay concentration) of the test and reference compounds (Table S1) were prepared in standard binding buffer (50 mM tris(hydroxymethyl)-aminomethane–HCl (Tris-HCl), 10 mM MgCl_2_, 1 mM ethylenediaminetetraacetate (EDTA), pH 7.4) by serial dilution: 0.05 nM, 0.5 nM, 1.5 nM, 5 nM, 15 nM, 50 nM, 150 nM, 500 nM, 1.5 μM, 5 μM, and 50 μM. The radioligand (Table S1) was diluted to five times the assay concentration in standard binding buffer. Aliquots (50 μL) of the radioligand were dispensed into the wells of a 96-well plate containing 100 μL of standard binding buffer. Triplicate aliquots (50 μL) of the test and reference compound dilutions were then added. Finally, crude membrane fractions (50 μL) of cells (HEK293 or CHO) expressing human recombinant receptors were dispensed into each well. A total 250 μL of the reaction mixtures was incubated at room temperature and shielded from light for 1.5 h, then harvested by rapid filtration onto Whatman GF/B glass fiber filters presoaked with 0.3% polyethyleneimine, by using a 96-well Brandel harvester.

Four rapid washes were performed with chilled standard binding buffer (500 μL) to decrease nonspecific binding. Filters were placed in 6 mL scintillation tubes and allowed to dry overnight. The next day, 4 ml of EcoScint scintillation cocktail (National Diagnostics) was added to each tube. The tubes were capped, labeled, and counted by liquid scintillation counting. The filter mats were dried, and the scintillant was melted onto the filters, then the radioactivity retained on the filters was counted in a Microbeta scintillation counter. The IC_50_ values were obtained by using the Prism 4.0 program (GraphPad Software, San Diego, CA) and converted into *K*i values. Each compound was tested in triplicate at least.

### 3.6. Drug-Like Properties

#### 3.6.1. Plasma Stability

Human plasma in each culture tube, treated with test compound (10 μM), was incubated 37 °C for 0, 30, and 120 min, respectively. In a determined period of time, an internal standard solution of chlorpropamide in acetonitrile was added into each culture tube, which was shaken with a vortex mixer for 5 min and centrifuged for additional 5 min (14,000 rpm, 4 °C). The supernatant was then injected into thr LC-MS/MS system to analyze the plasma stability of compound **10a**.

#### 3.6.2. Microsomal Stability

To human liver microsomes (0.5 mg/mL) were added 0.1 M phosphate buffer (pH 7.4) and tested compounds (1 μM). After incubation at 37 °C for 5 min, NADPH generation system solution was also added and incubated at 37 °C for 30 min again. To terminate reaction, acetonitrile including internal standard (chlorpropamide) was added, and the solution was centrifuged for 5 min (14,000 rpm, 4 °C). The supernatant was then injected into the LC-MS/MS system to analyze the microsomal stability of compound **10a**.

#### 3.6.3. CYP Inhibition

To human liver microsomes (0.25 mg/mL), 0.1 M phosphate buffer (pH 7.4), a cocktail of five probe substrates (Phenacetin 50 μM, Diclofenac 10 μM, S-mephenytoin 100 μM, Dextromethorphan 5 μM, and Midazolam 2.5 μM), and tested compounds were added at concentrations of 0 μM (as a control) and 10 μM. After incubation at 37 °C for 5 min, NADPH generation system solution was also added and incubated at 37 °C for 15 min again. To terminate the reaction, acetonitrile including internal standard (Terfenadine) was added, and the solution was centrifuged for 5 min (14,000 rpm, 4 °C). The supernatant was then injected into the LC-MS/MS system to simultaneously analyze the metabolites of the probe substrates and evaluate the % CYP inhibition of the tested compound **10a**.

## 4. Conclusions

Thus, we synthesized two series of disubstituted pyrimidine derivatives as potential 5-HT_2C_ selective agonists. Initially, a cell-based assay of 2,5-disubstituted pyrimidines revealed that most compounds did not have good agonistic effects towards the 5-HT_2C_ receptor, although 2-piperazinyl-5-(3-fluorobenzyloxy)pyrimidine **9b** showed effective biological activity, with 61% activation. In vitro evaluation of the second series of pyrimidines, possessing different cyclic amines, demonstrated that four compounds showed greater than 50% activation against 5-HT_2C_ in the primary cell-based assay. In the secondary binding affinity assay, compounds **10a** and **10f**, with 1,4-diazepane at the 2-position of pyrimidine, were identified as the most potent 5-HT_2C_ ligands, with excellent *K*i values of 7.9 nM and 19.0 nM, respectively. Compound **10a** showed high selectivity profiles for other 5-HT receptor subtypes, with a greater than 10-fold difference in potency. Additional in vitro stability experiments indicated that compound **10a** has excellent plasma and microsomal stability, along with low CYP inhibition. Based on these results, 2,4-disubstituted pyrimidine **10a** could be considered a potential lead compound as a 5-HT_2C_ selective agonist for further application in chemical probes such as a 5-HT_2C_ PET radiotracer.

## Figures and Tables

**Table 1:** In vitro agonistic activity of 2,5-disubstituted pyrimidine **9a–r** against 5-HT_2C_. 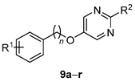

Entry	Compds	n	R^1^	R^2^	%activation (10 μM)
1	**9a**	0	3-F	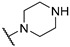	7
2	**9b**	1	3-F		61
3	**9c**	2	3-F		15
4	**9d**	0	4-F		5
5	**9e**	1	4-F		18
6	**9f**	2	4-F		4
7	**9g**	0	3-F	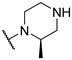	35
8	**9h**	1	3-F		48
9	**9i**	2	3-F		40
10	**9j**	0	4-F		26
11	**9k**	1	4-F		48
12	**9l**	2	4-F		32
13	**9m**	0	3-F	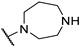	16
14	**9n**	1	3-F		−1
15	**9o**	2	3-F		3
16	**9p**	0	4-F		14
17	**9q**	1	4-F		16
18	**9r**	2	4-F		−1
19	**Lorcaserin (1)**				94^a^

^a^ %activation value at 1 μM, EC_50_ = 14 nM.

**Table 2:** In vitro activity of 2,4-disubsitutued pyrimidine against 5-HT_2C._ 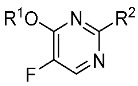

Entry	Compds	R^1^	R^2^	%activation^a^	%binding	*K*i (nM)
1	**10a**	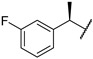	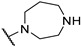	81.0	81.3	7.9
2	**10b**		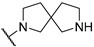	78.2	47.6	ND
3	**10c**		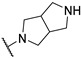	29.5	73.6	295.0
4	**10d**		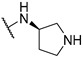	16.4	88.6	119.0
5	**10e**		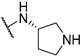	7.7	61.4	1255.0
6	**10f**	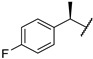	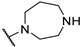	52.4	93.9	19.0
7	**10g**		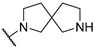	ND	78.1	232.0
8	**10h**		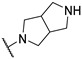	10.1	50.8	466.0
9	**10i**		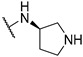	ND	85.9	120.0
10	**10j**		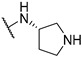	0.1	26.2	ND
11	**20a**	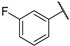	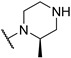	56.0	64.8	660.0
12	**20b**	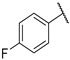	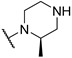	34.0	54.8	1107.0
13	**8**			N/A^b^	89.0	5.9

^a^ Serotonin was used as a reference compound (EC_50_ = 2.6 nM). ^b^ Not available.

**Table 3:** Evaluation of binding affinity of **10a** and **10f** to 5-HT receptor subtypes ^a^.

	5-HT Subtypes (%Binding at 10 μM/*K*i (nM))										
	1A	1B	1D	1E	2A	2B	2C	3	5A	6	7
**10a**	74.1 /578.0	56.1 /3237.0	71.4 /679.0	43.1 /ND^b^	75.5 /1284.0	98.0 /83.0	81.3 /7.9	89.0 /323.0	22.5 /ND	50.7 /348.0	61.3 /1016.0
**SI**	73.2	409.7	85.9	-	162.5	10.5	1.0	40.9	23.4	44.1	128.6
**10f**	67.6 /1066.0	19.2 /ND^b^	81.3 /937.0	31.3 /ND^b^	52.9 /2863.0	95.0 /118.0	98.9 /19.0	88.0 /415.0	15.2 /ND	56.5 /467.0	55.4 /616.0
**SI**	56.1	-	49.3	-	150.7	6.2	1.0	21.8	10.3	24.6	32.4
**8^c^**	89.1 /271.0	4.9 /ND^b^	83.4 /1052.0	48.0 /ND^b^	85.3 /511.0	98.1 /38.0	89.0 /5.9	83.4 /543.0	18.5 /ND^b^	56.7 /30.0	63.4 /528.0
**SI**	45.9	-	178.3	-	86.6	6.4	1.0	92.0	-	5.1	89.5

^a^ 5-HT receptor binding was determined by competitive binding assay using radioligands and reference compounds in Table S1. ^b^ Not determined due to low %binding. ^c^ Compound **8** was used as a reference compound. SI, selectivity index.

**Table 4:** The results of plasma stability, microsomal stability, and CYP inhibition.

Compd	Plasma Stability %Remaining @ 10 μM after 0.5 and 2 h	HLM %Remaining @ 1 μM after 0.5 h	CYP Isozymes (%Remaining @ 10 μM)				
			1A2	2D6	2C9	3A4	2C19
**10a**	99.9/98.0	96.6	86.5	63.6	>100	86.2	86.3

## Supplementary Materials

The following are available online at https://www.mdpi.com/1420-3049/24/18/3234/s1, (NMR spectral data of compounds **9a**–**9r**, **10a**–**10j**, and **20a/b**; Table S1: A list of 5-HT receptor radioligands and reference compounds for binding assay).
