# Supplementary material for: Synthesis and Biological Evaluation of Disubstituted Pyrimidines as Selective 5-HT2C Agonists
Source: Molecules. 2019 Sep 5;24(18):3234. doi: 10.3390/molecules24183234 (PMC6767204; doi:10.3390/molecules24183234)
Supplement: Supplementary file 1 [file molecules-24-03234-s001.pdf]

Supplementary Information

Synthesis and Biological Evaluation of Disubstituted Pyrimidines as Selective 5-HT$_{2C}$ agonists

Juhyeon Kim[1,2,#], Yoon Jung Kim[3,#], Ashwini M. Londhe[4,5], Ae Nim Pae[4,5], Hyunah Choo[1,4], Hak Joong Kim[2] and Sun-Joon Min[3,6,*]

[1] Center for Neuro-Medicine, Korea Institute of Science and Technology (KIST), Seoul, 02792, Republic of Korea

[2] Department of Chemistry, Korea University, Seoul, 02841, Republic of Korea

[3] Department of Applied Chemistry, Hanyang University, Ansan, Gyeonggi-do, 15588, Republic of Korea

[4] Division of Bio-Medical Science & Technology, KIST School, Korea University of Science and Technology, Seoul 02792, Republic of Korea

[5] Convergence Research Center for Diagnosis, Treatment and Care System of Dementia, Korea Institute of Science and Technology, Seoul, 02792, Republic of Korea

[6] Department of Chemical & Molecular Engineering, Hanyang University, Ansan, Gyeonggi-do, 15588, Republic of Korea

# Both authors contributed equally to this work.

Email: sjmin@hanyang.ac.kr

Table of Contents

1. NMR spectral data of 2,5-disubstituted pyrimidine **9a-9i** S2

2. NMR spectral data of 2,4-disubstituted pyrimidine **10a-10j,** and **20a/b** S20

3. Radioligands and reference compounds for binding assay S32

1. NMR spectral data of 2,5-disubstituted pyrimidine **9a**-**9i**

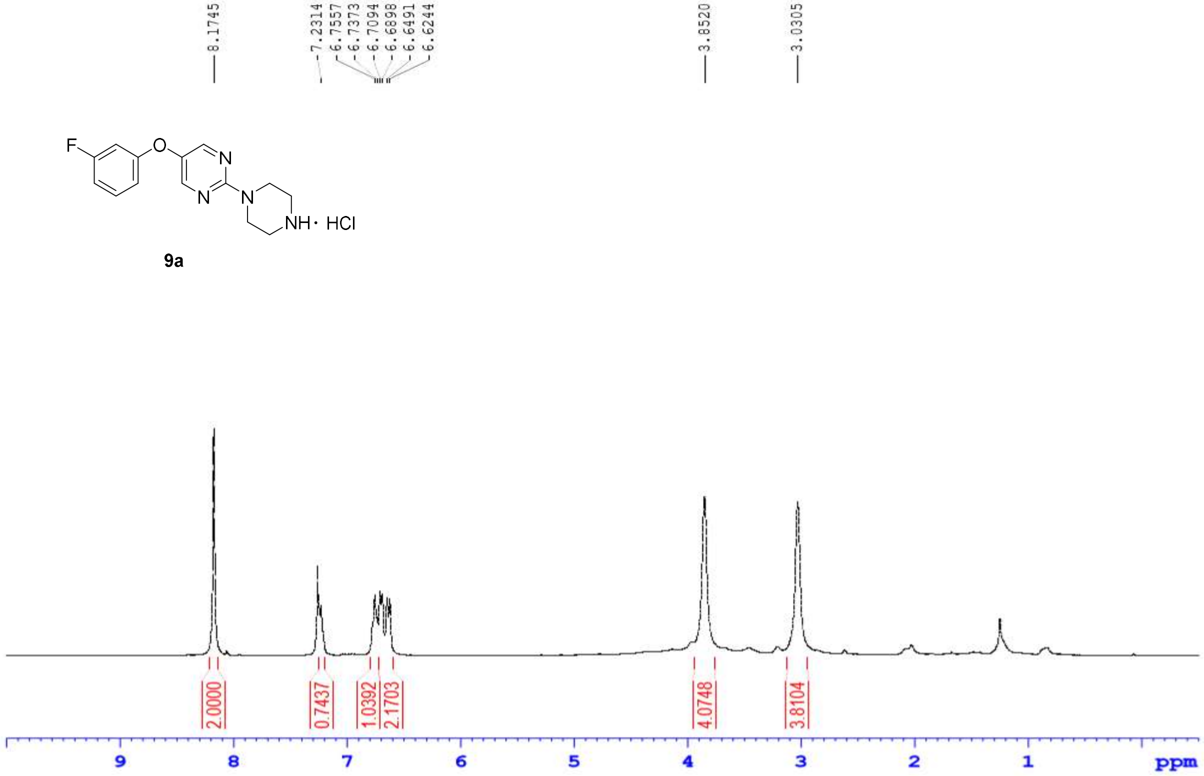

^{1}H NMR Spectrum of Compound **9a** (400 MHz)

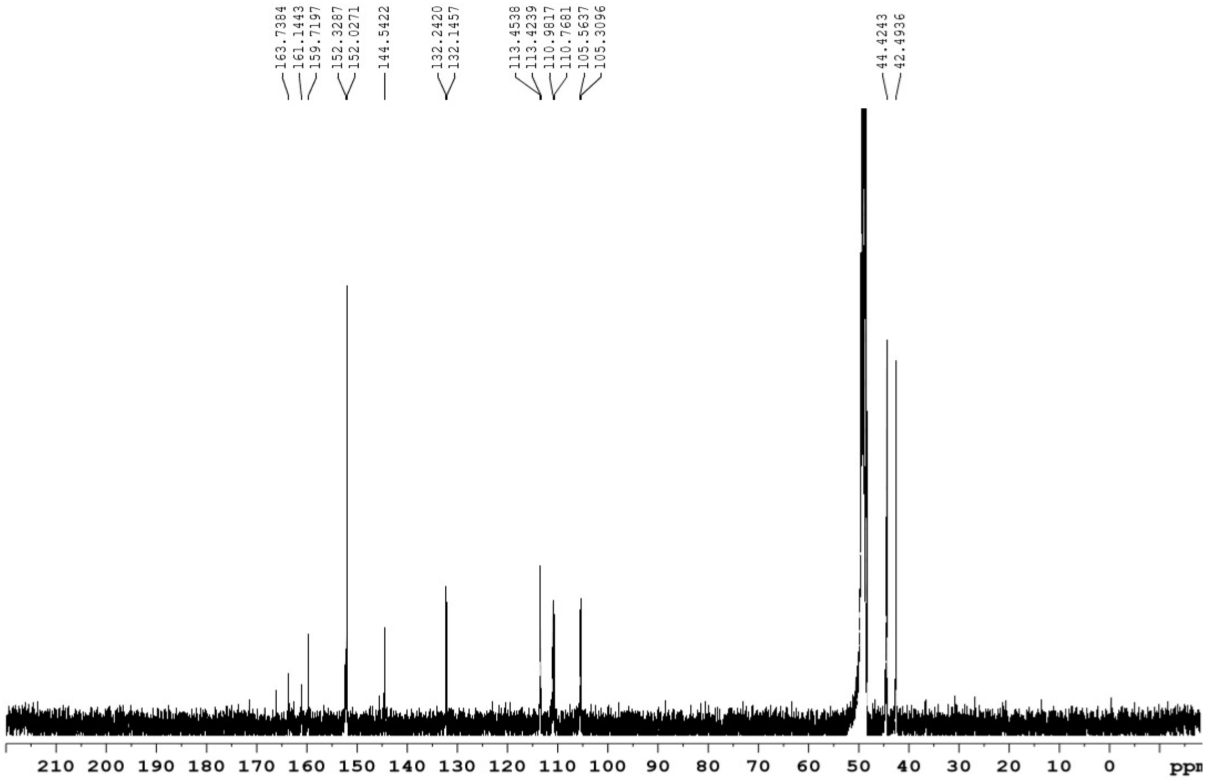

^{13}C NMR Spectrum of Compound **9a** (100 MHz)

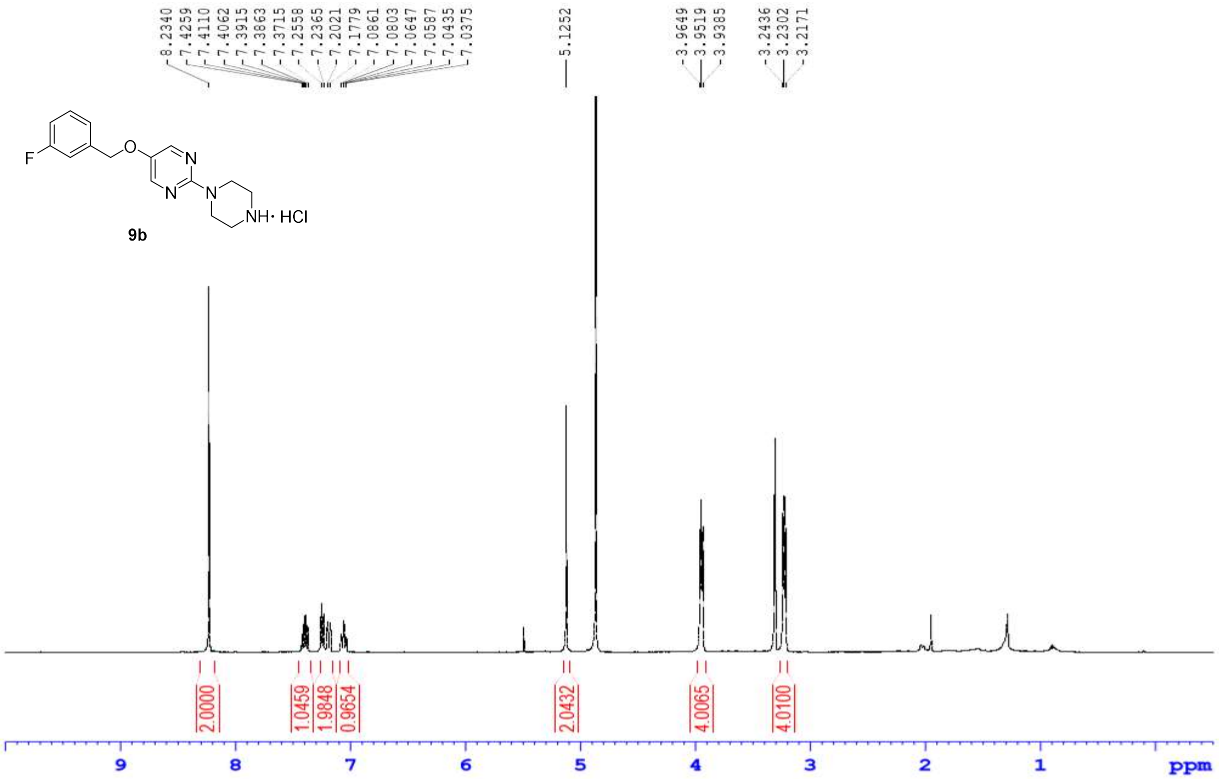

^{1}H NMR Spectrum of Compound **9b** (400 MHz)

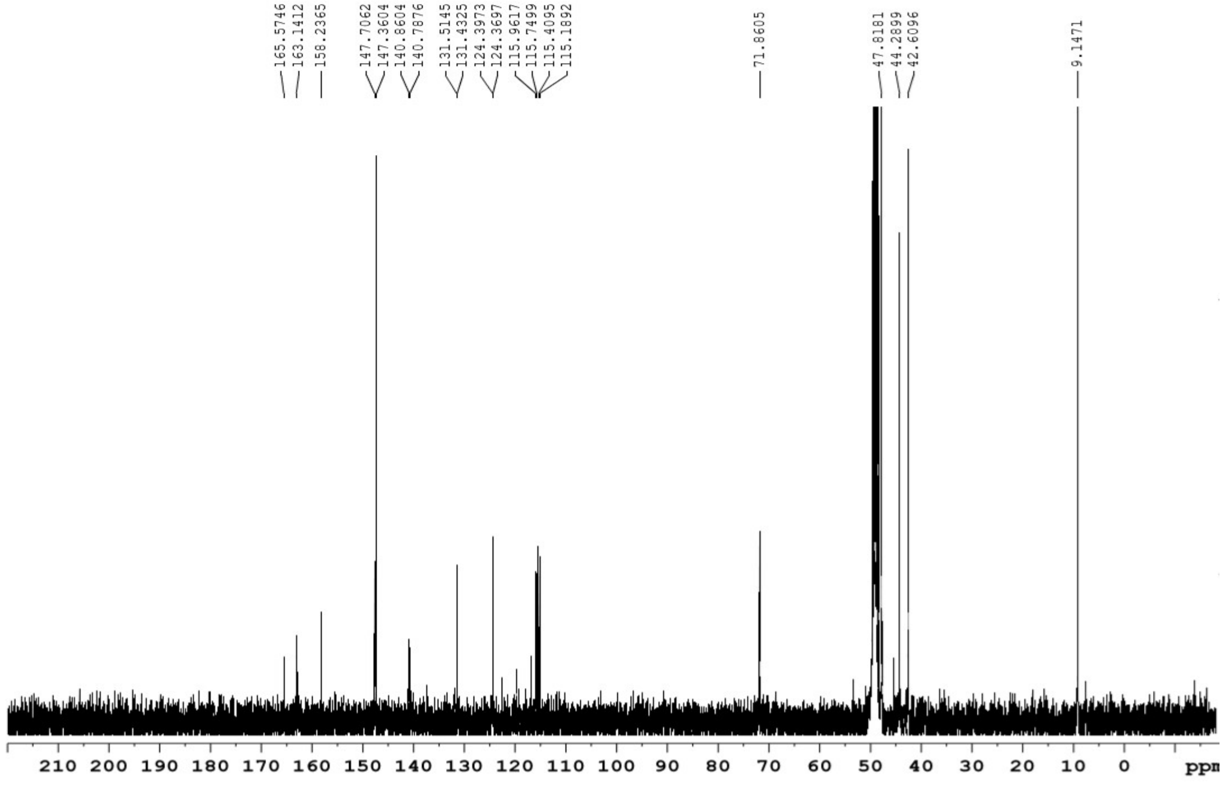

^{13}C NMR Spectrum of Compound **9b** (100 MHz)

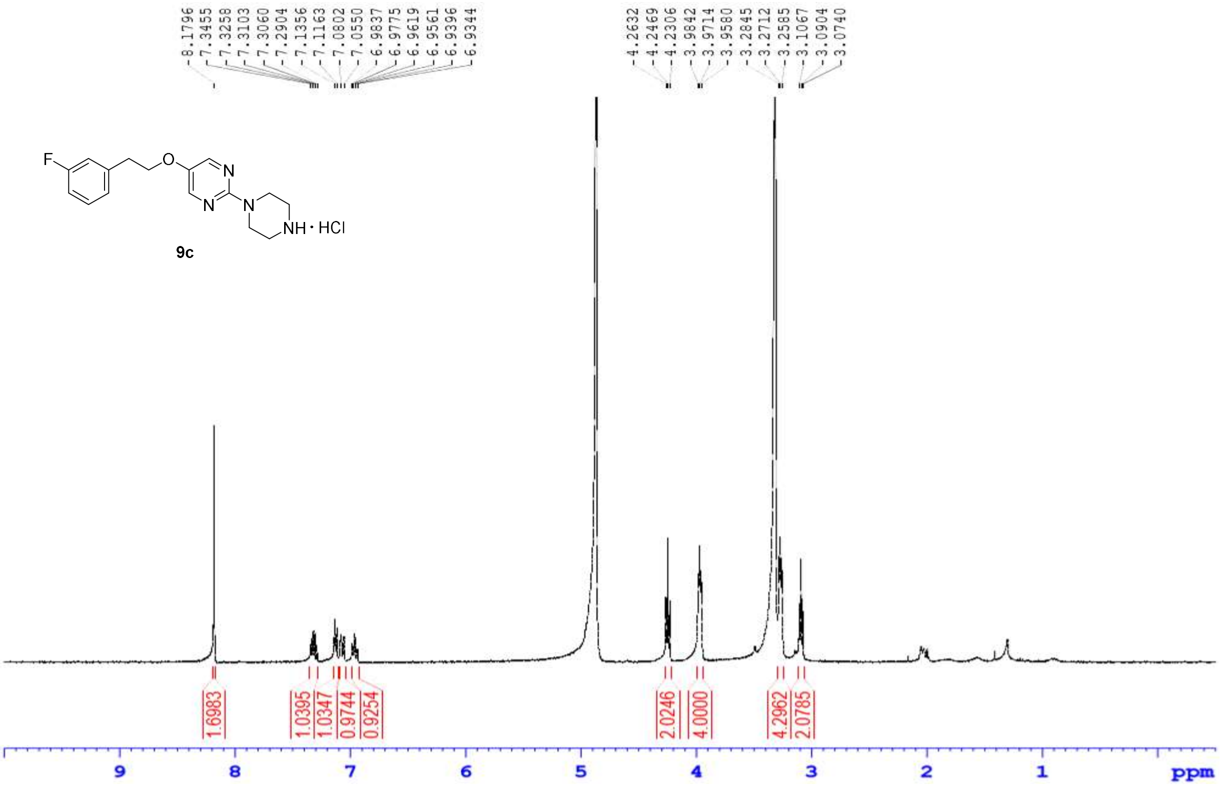

^{1}H NMR Spectrum of Compound **9c** (400 MHz)

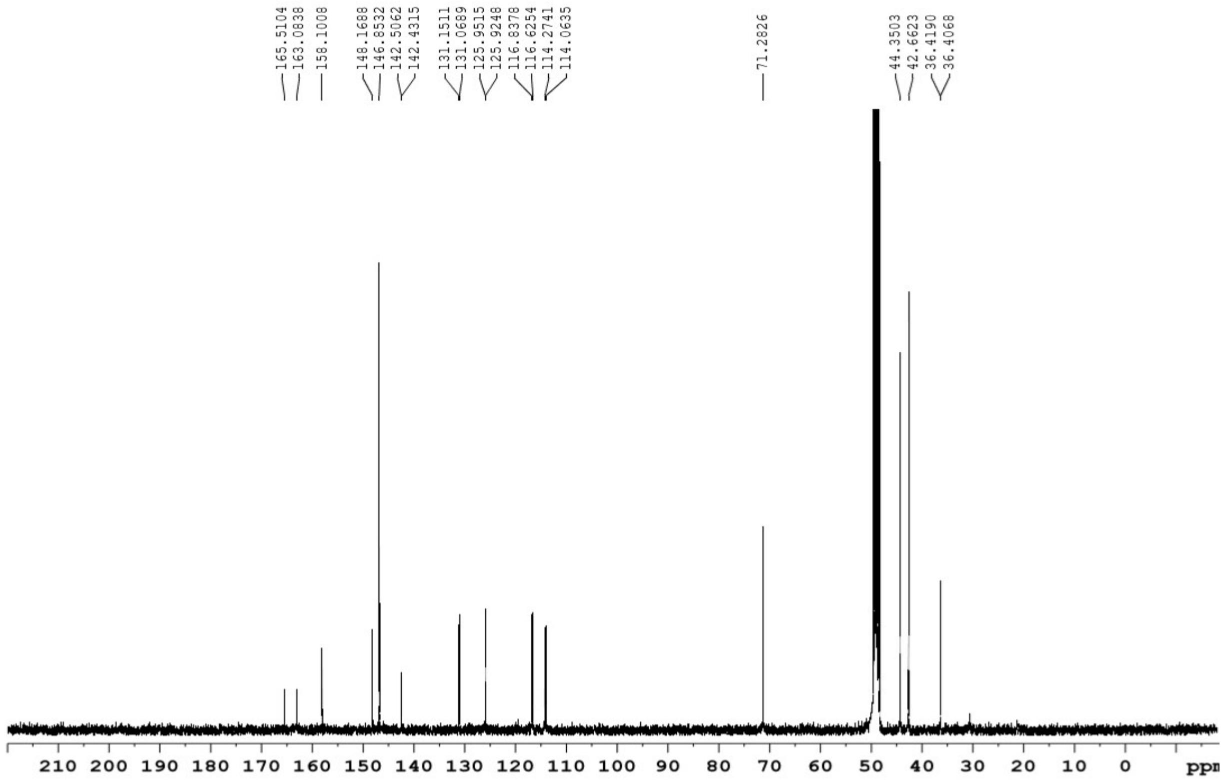

^{13}C NMR Spectrum of Compound **9c** (100 MHz)

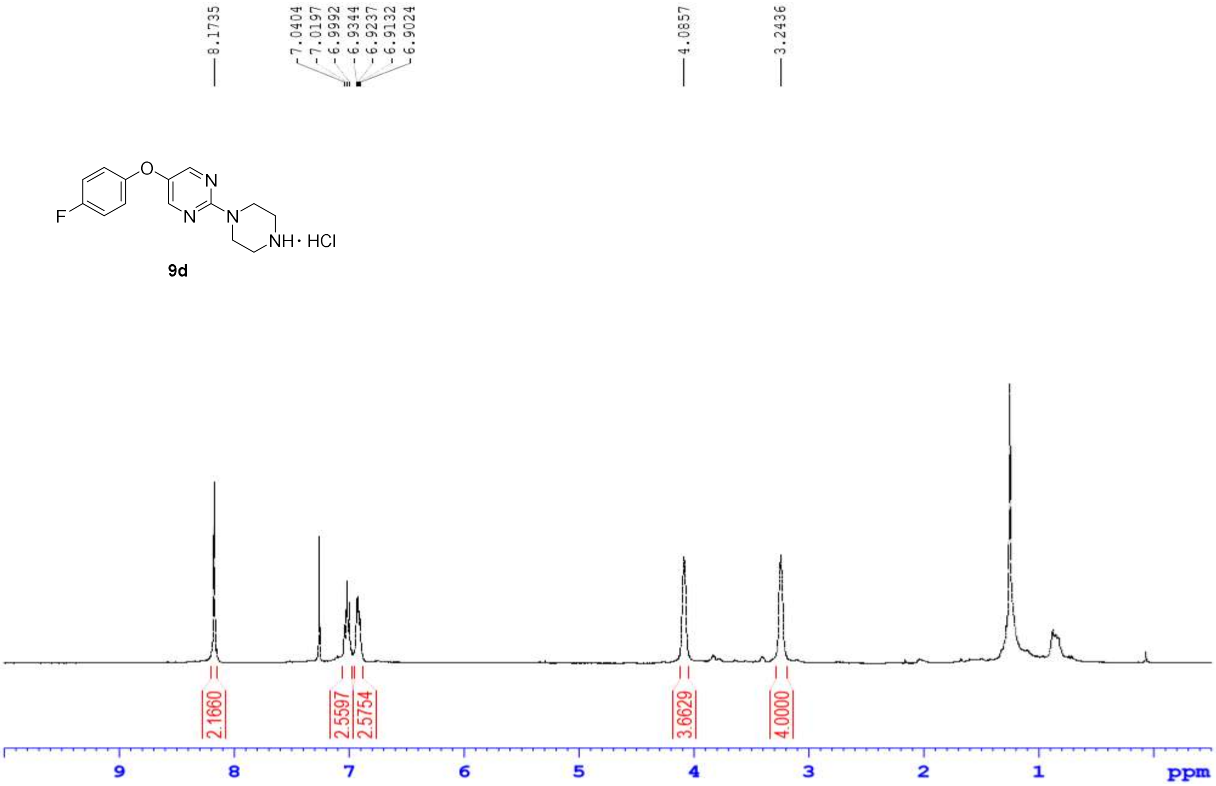

^{1}H NMR Spectrum of Compound **9d** (400 MHz)

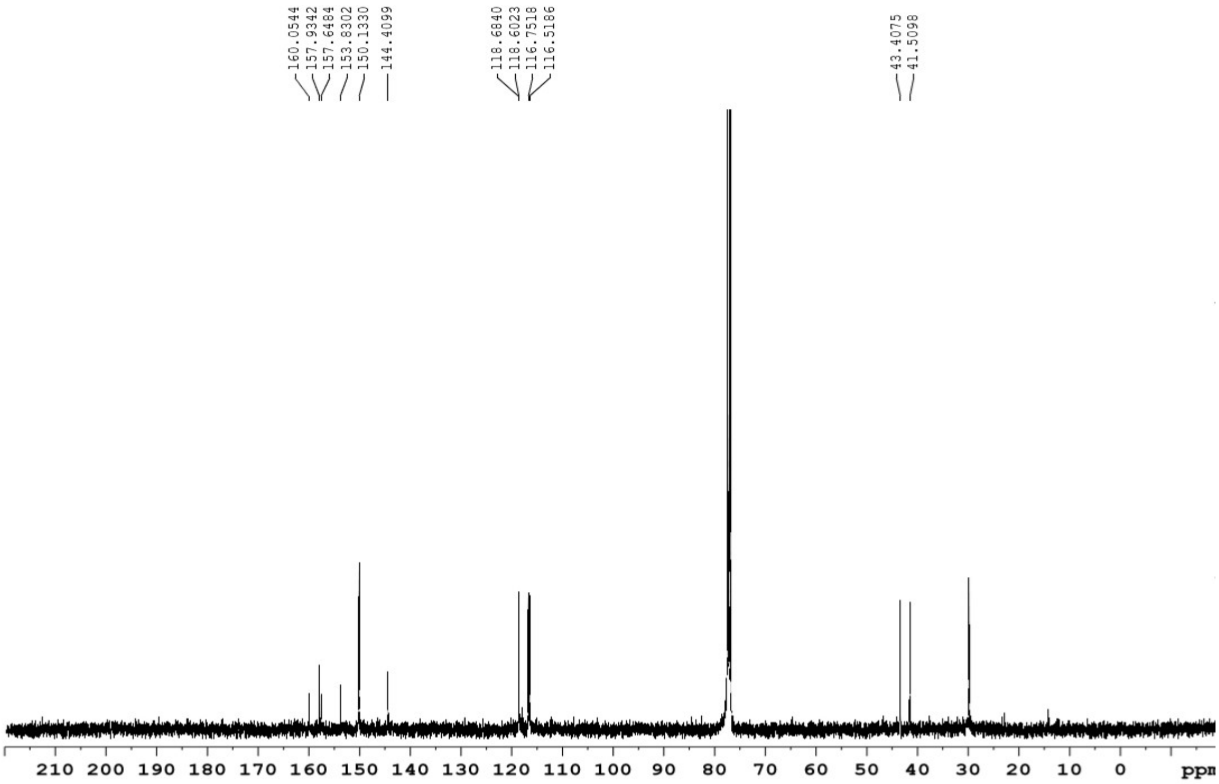

^{13}C NMR Spectrum of Compound **9d** (100 MHz)

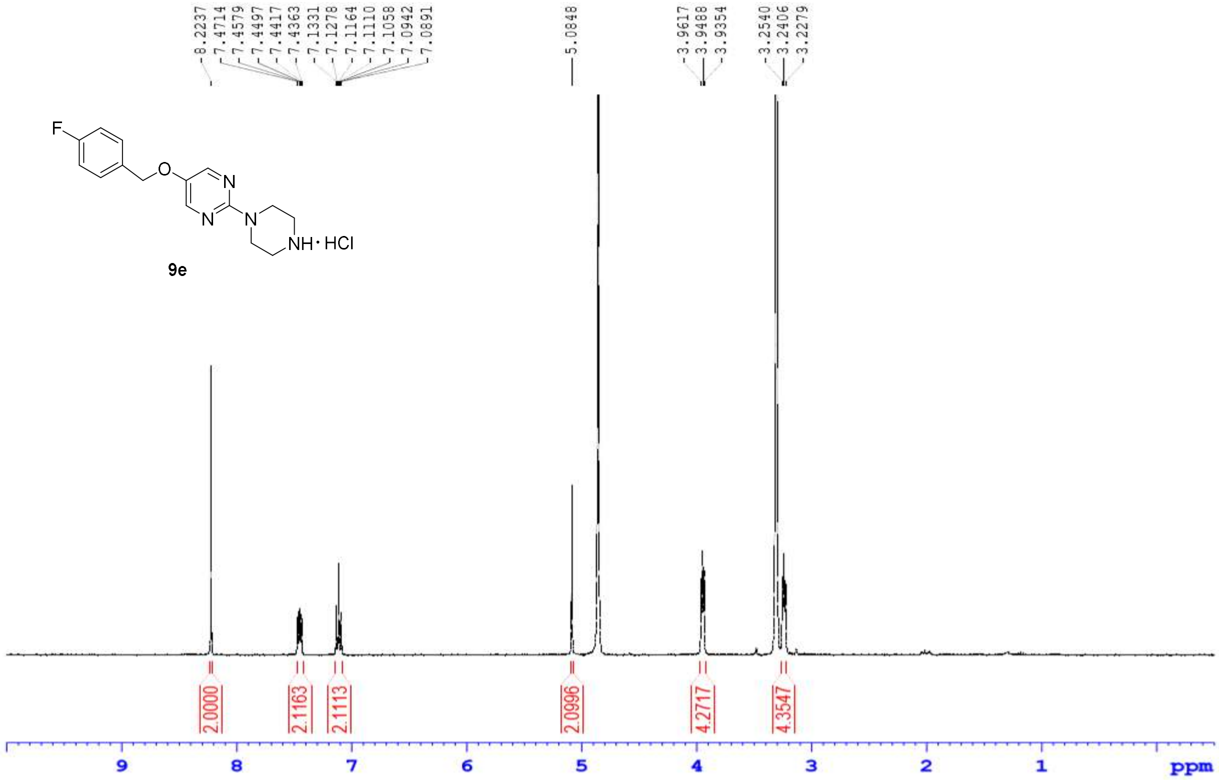

^{1}H NMR Spectrum of Compound **9e** (400 MHz)

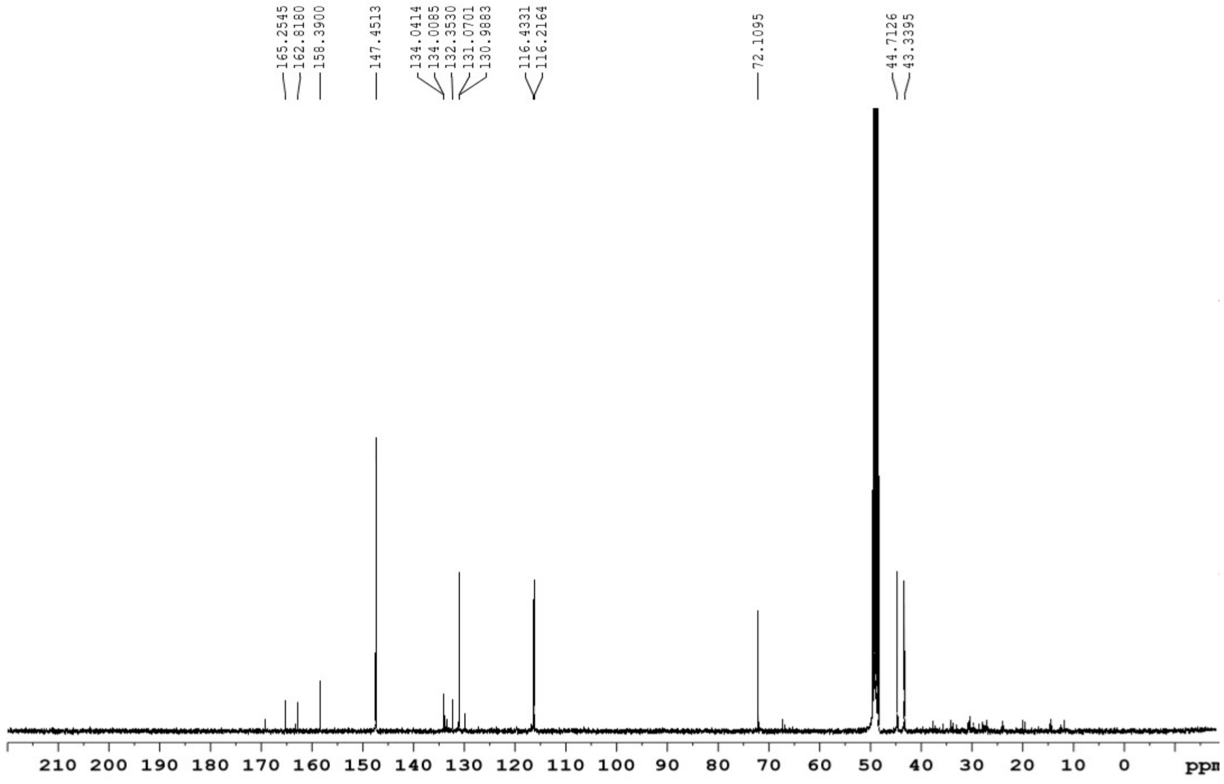

^{13}C NMR Spectrum of Compound **9e** (100 MHz)

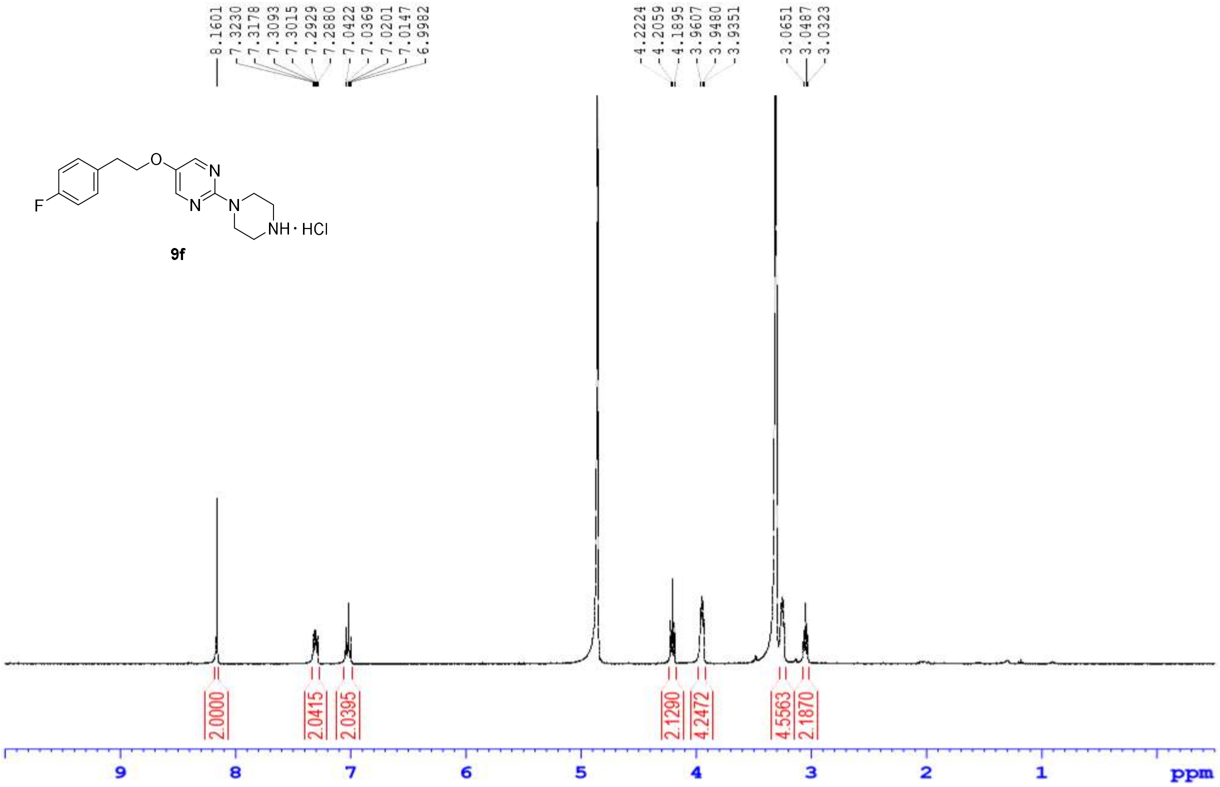

^{1}H NMR Spectrum of Compound **9f** (400 MHz)

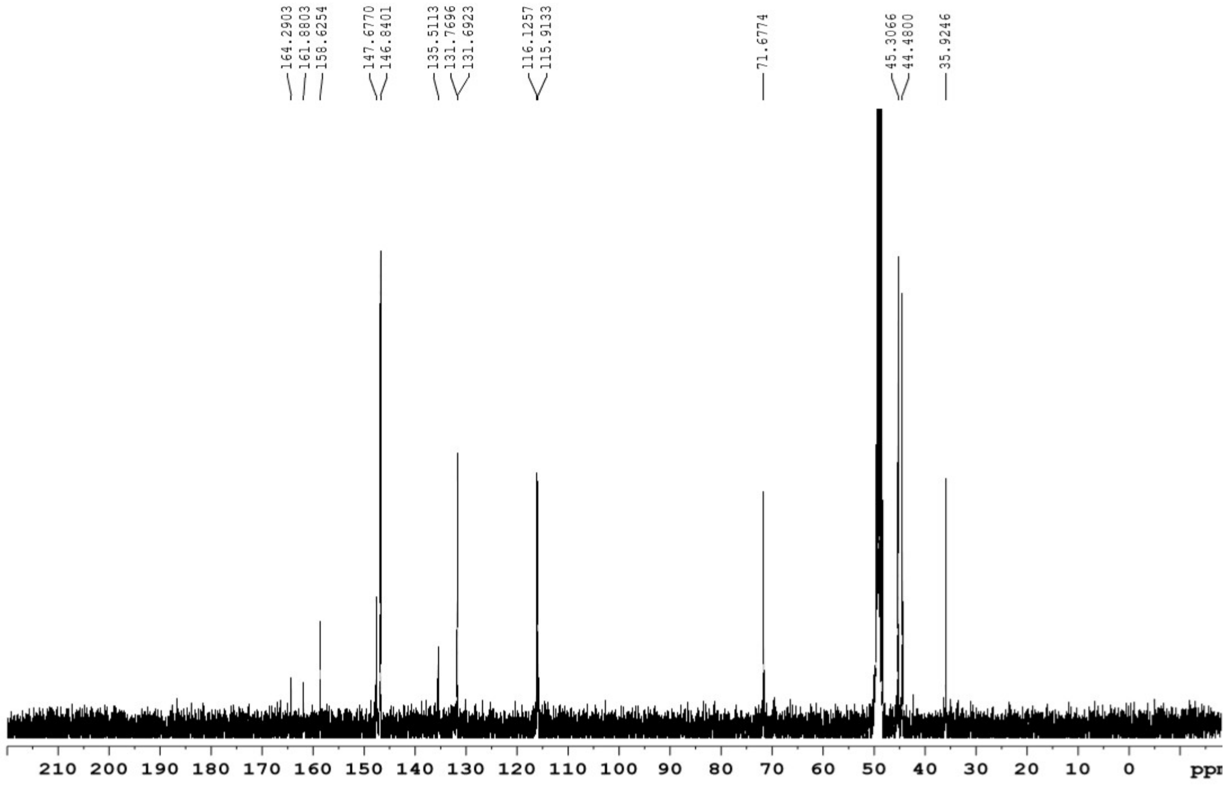

^{13}C NMR Spectrum of Compound **9f** (100 MHz)

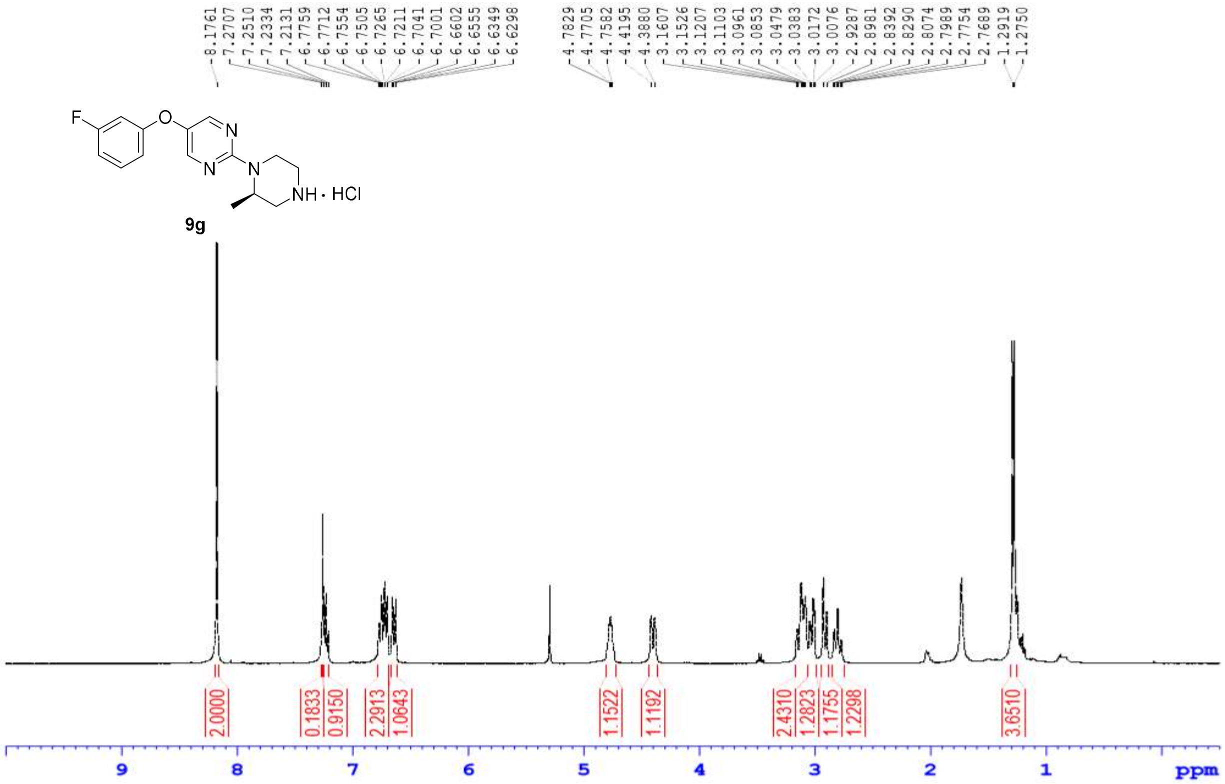

^{1}H NMR Spectrum of Compound **9g** (400 MHz)

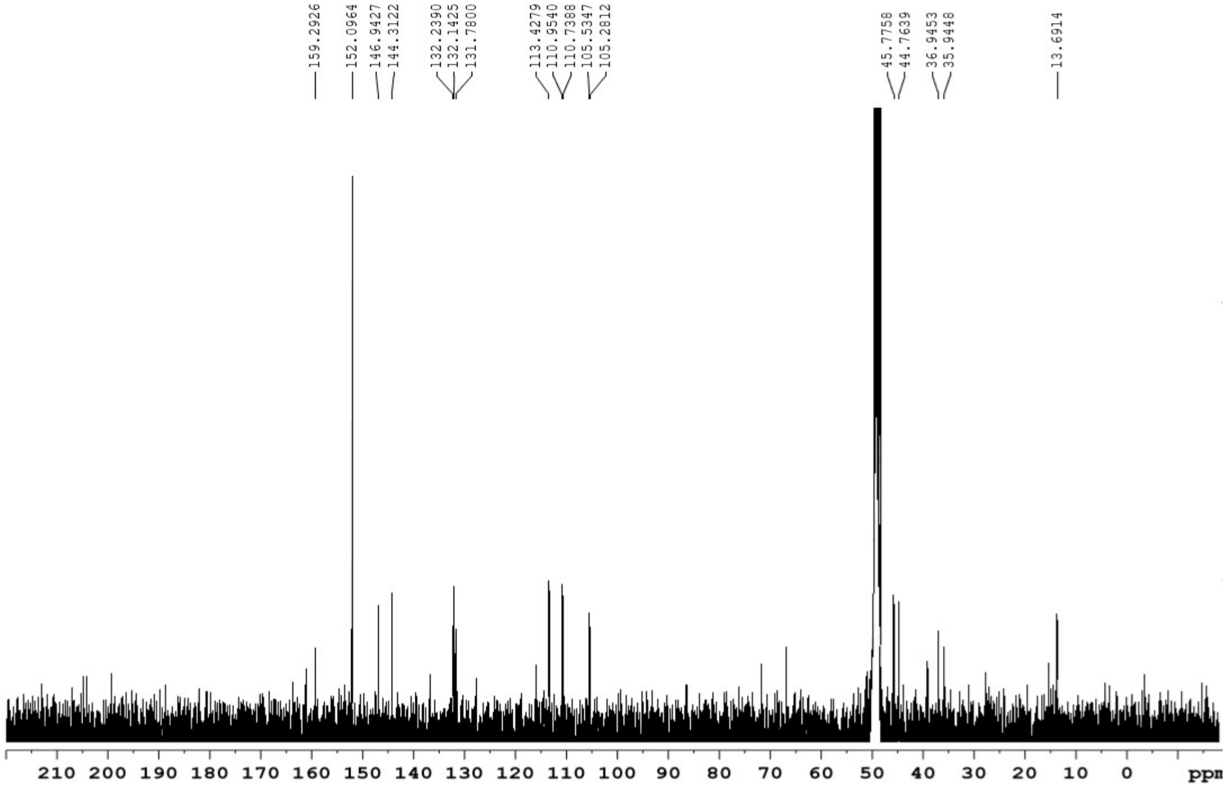

^{13}C NMR Spectrum of Compound **9g** (100 MHz)

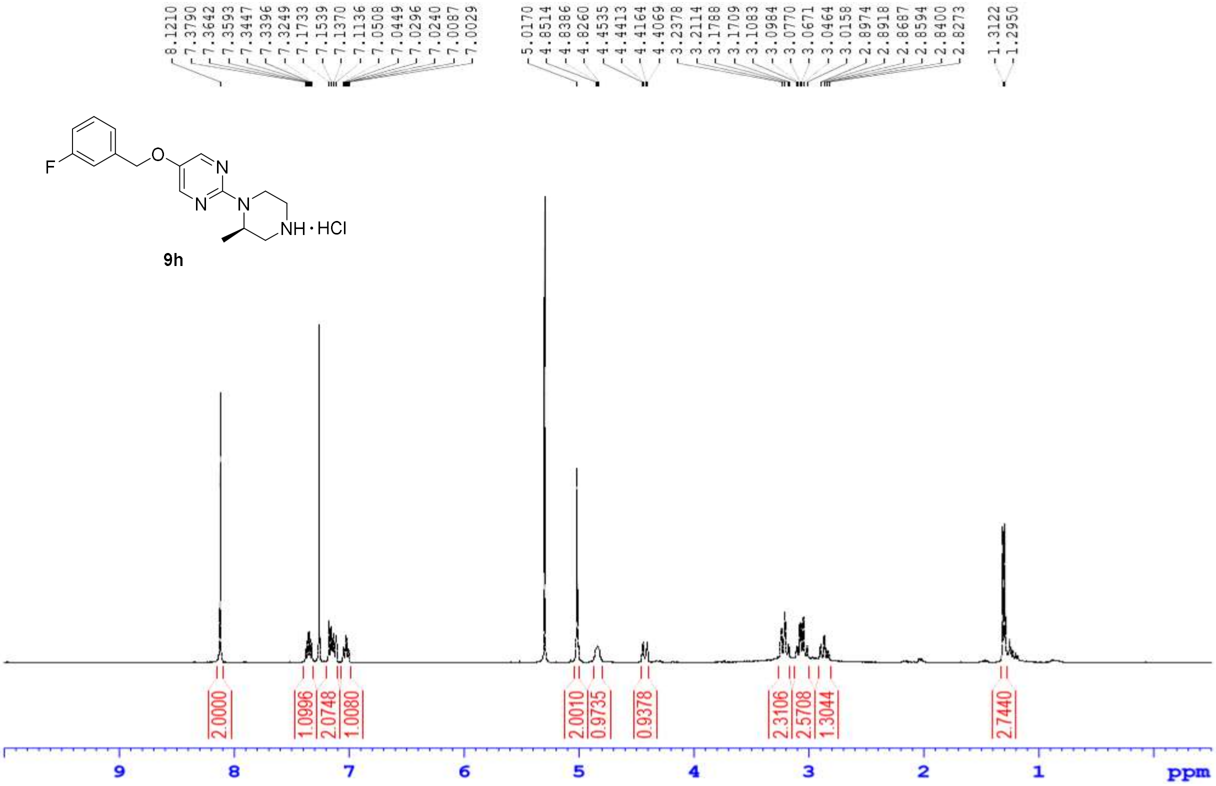

^{1}H NMR Spectrum of Compound **9h** (400 MHz)

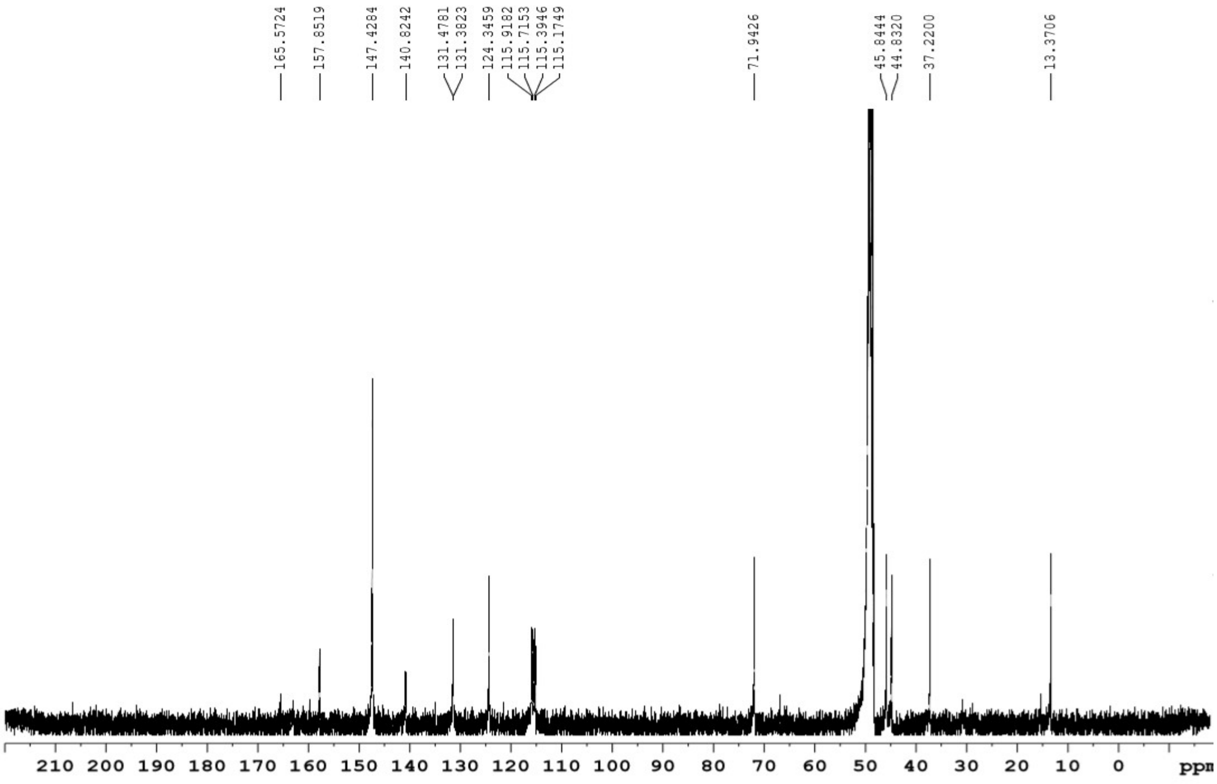

^{13}C NMR Spectrum of Compound **9h** (100 MHz)

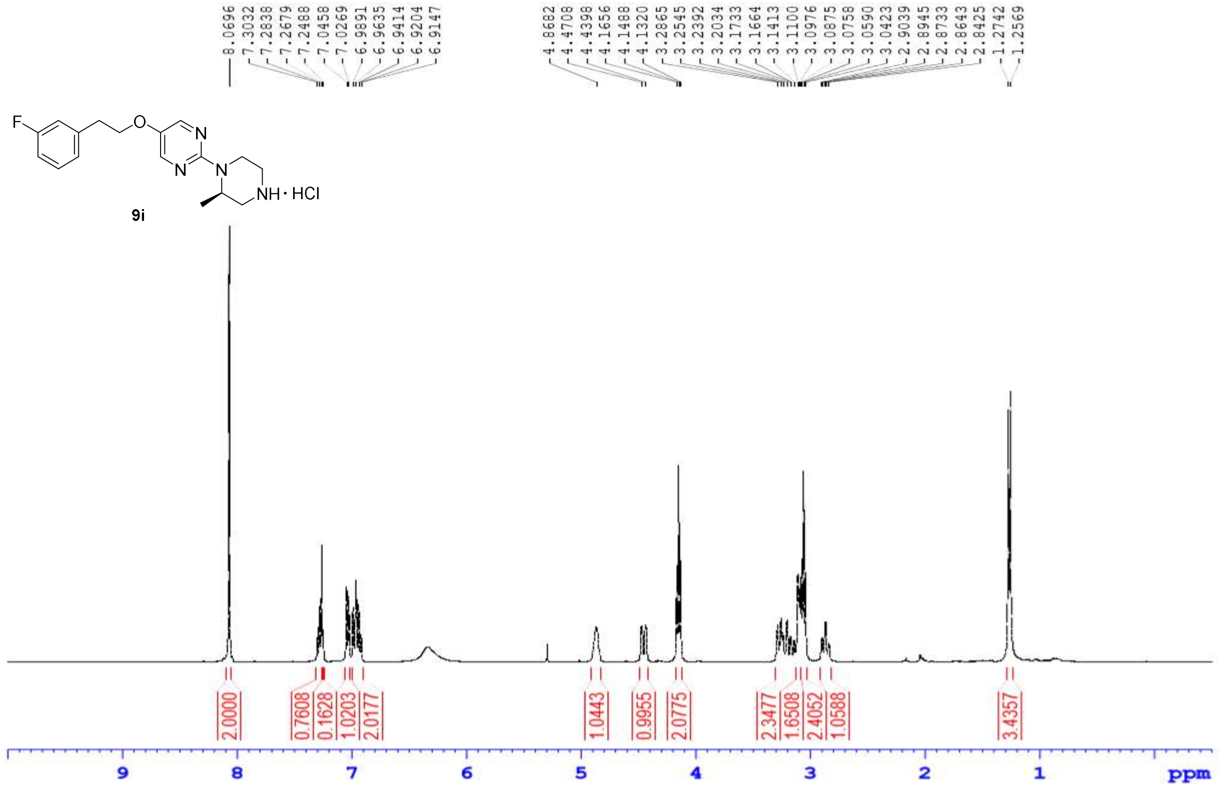

^{1}H NMR Spectrum of Compound **9i** (400 MHz)

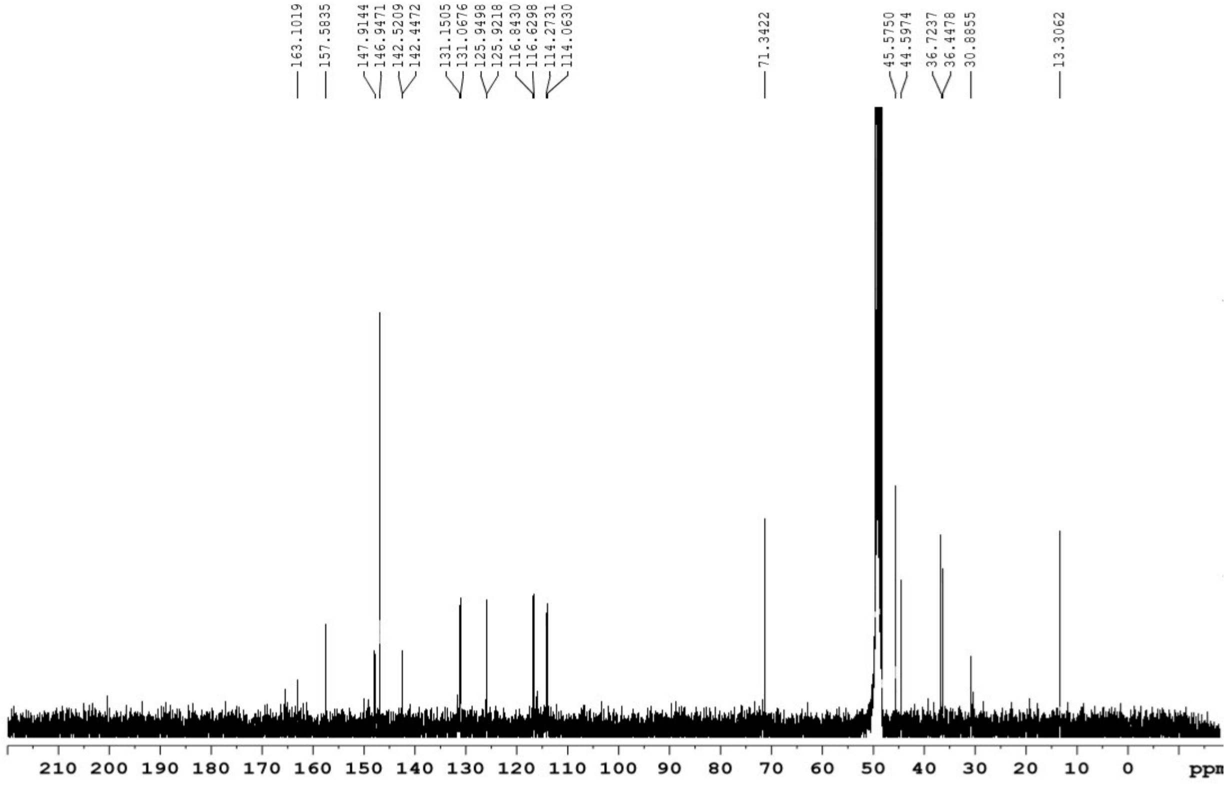

^{13}C NMR Spectrum of Compound **9i** (100 MHz)

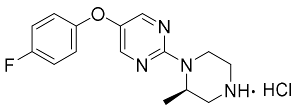

9j

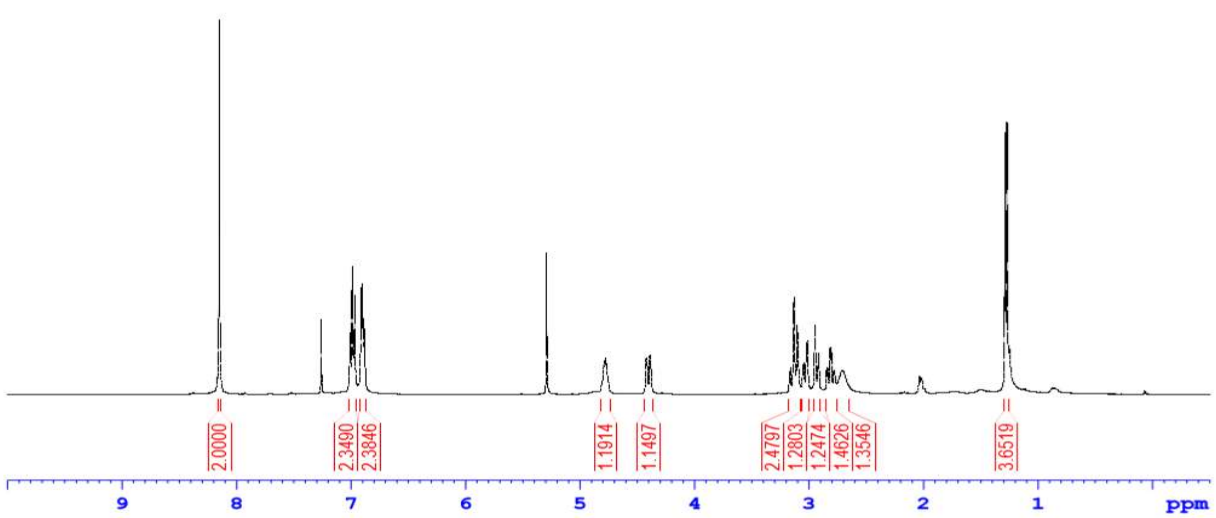

^{1}H NMR Spectrum of Compound **9j** (400 MHz)

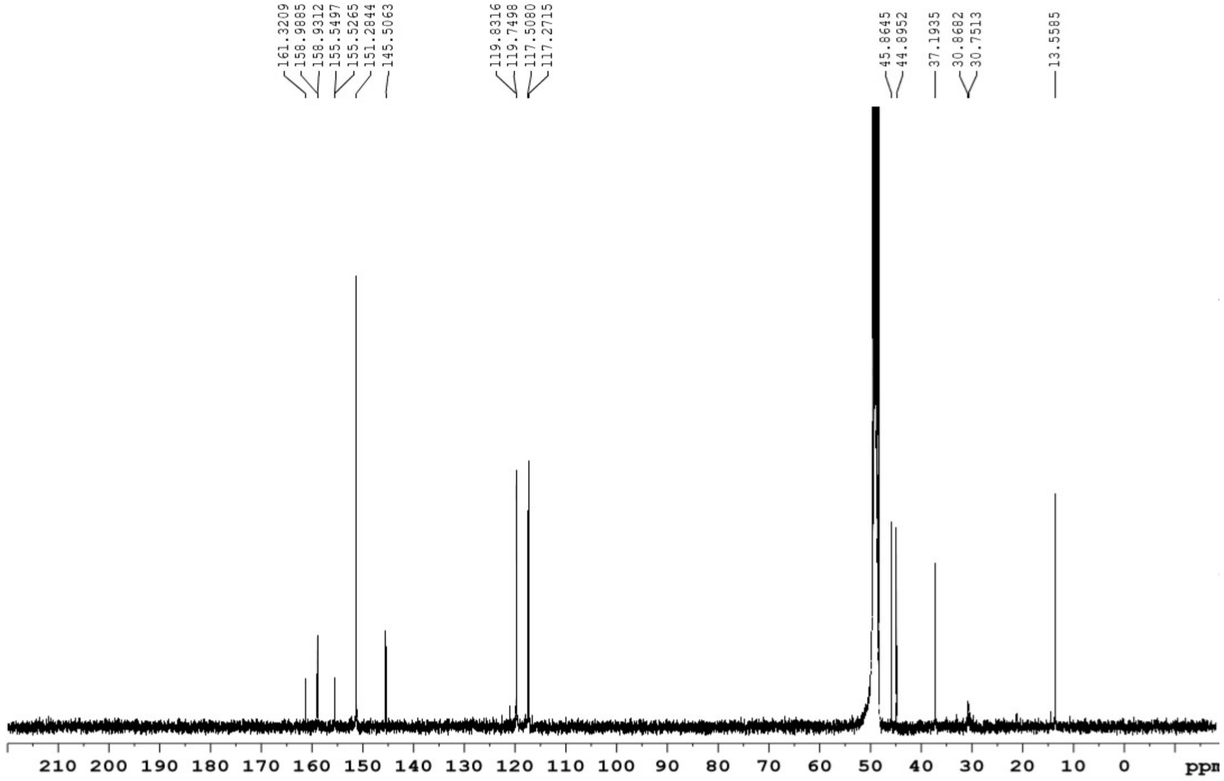

^{13}C NMR Spectrum of Compound **9j** (100 MHz)

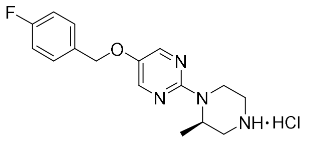

9k

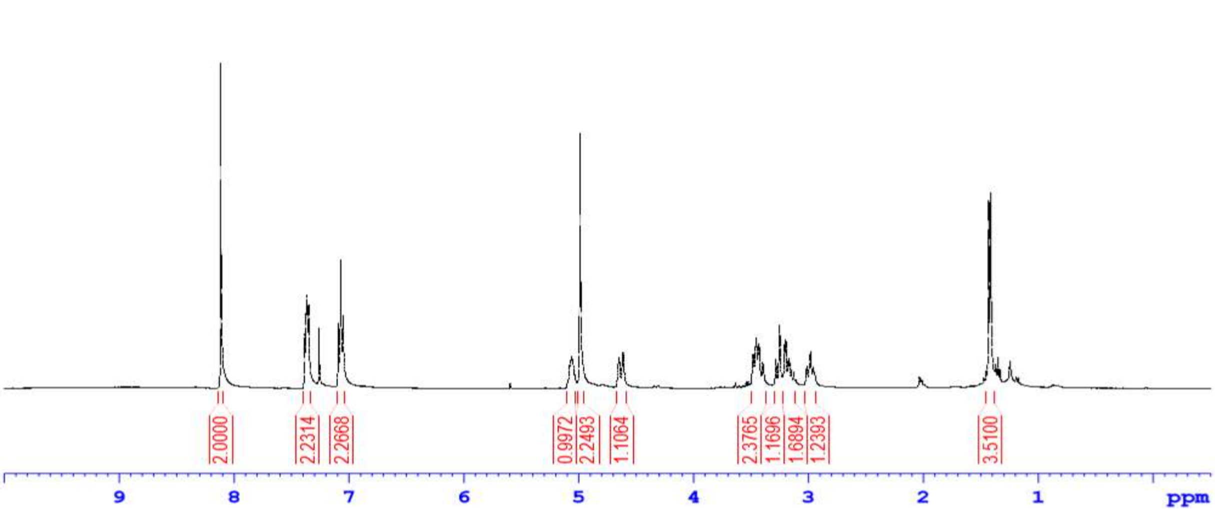

^{1}H NMR Spectrum of Compound **9k** (400 MHz)

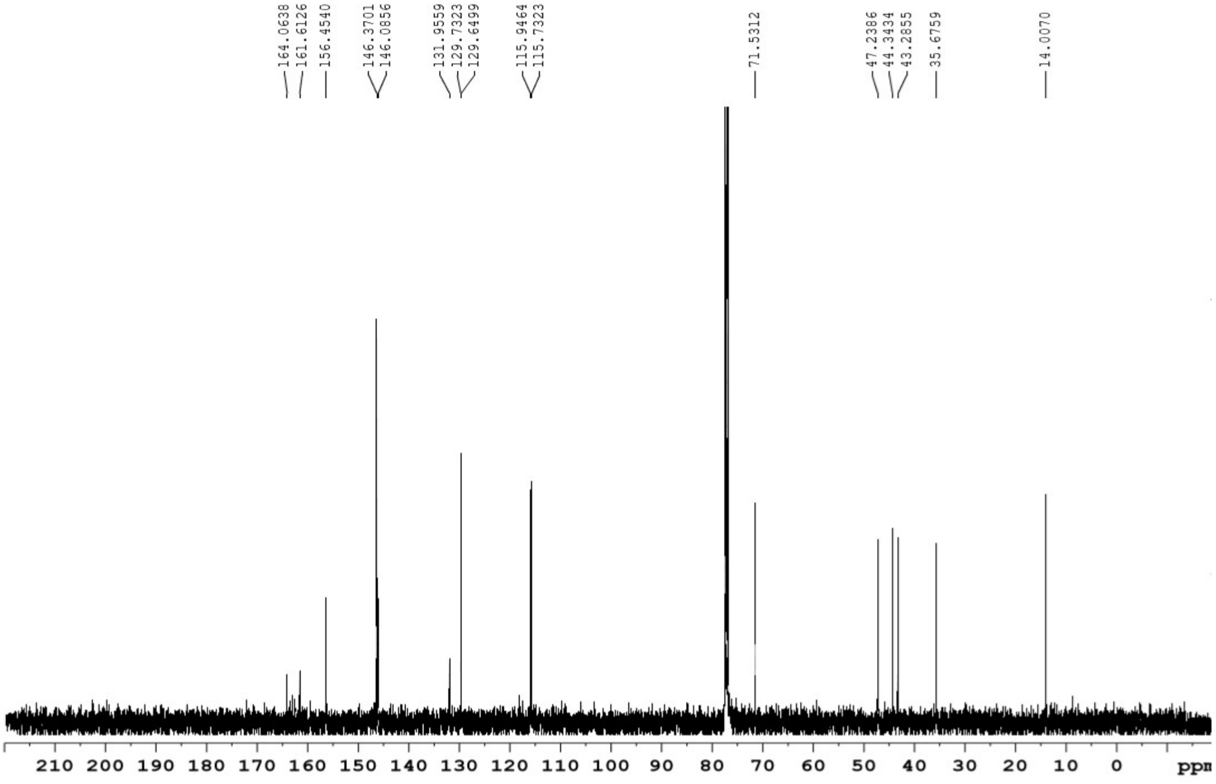

^{13}C NMR Spectrum of Compound **9k** (100 MHz)

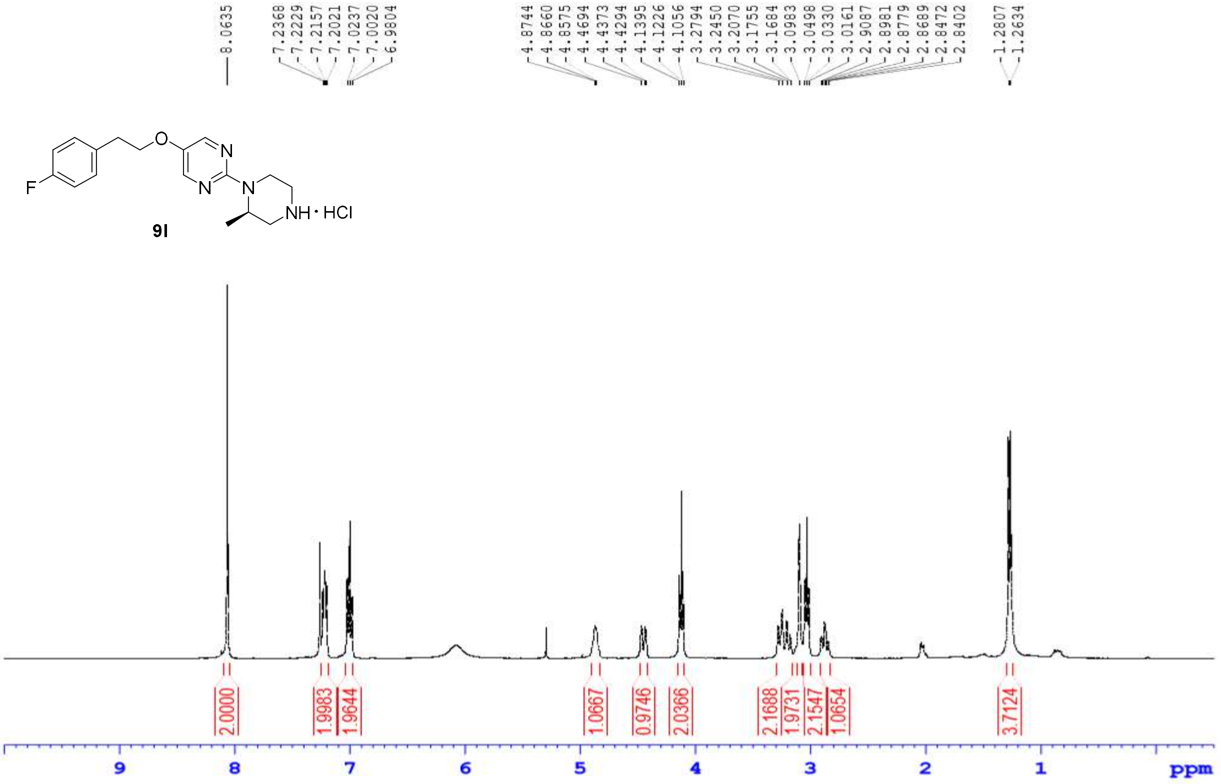

^{1}H NMR Spectrum of Compound **9l** (400 MHz)

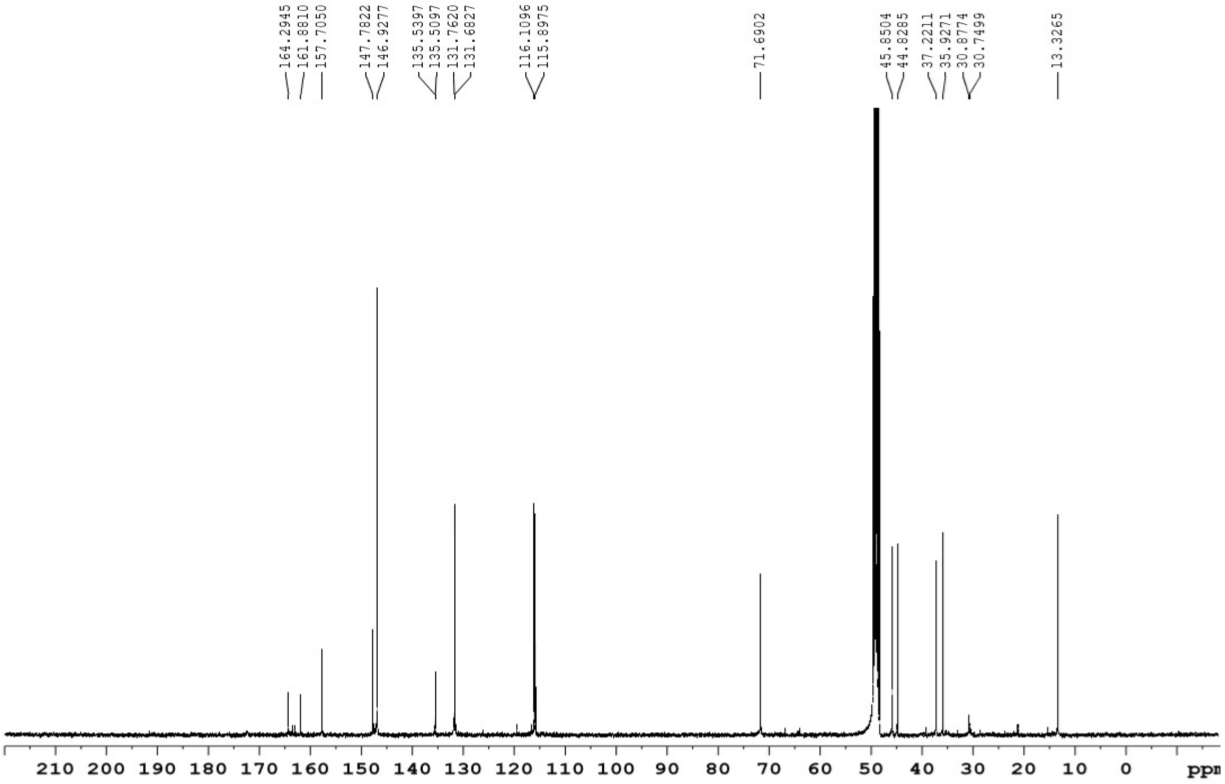

^{13}C NMR Spectrum of Compound **9k** (100 MHz)

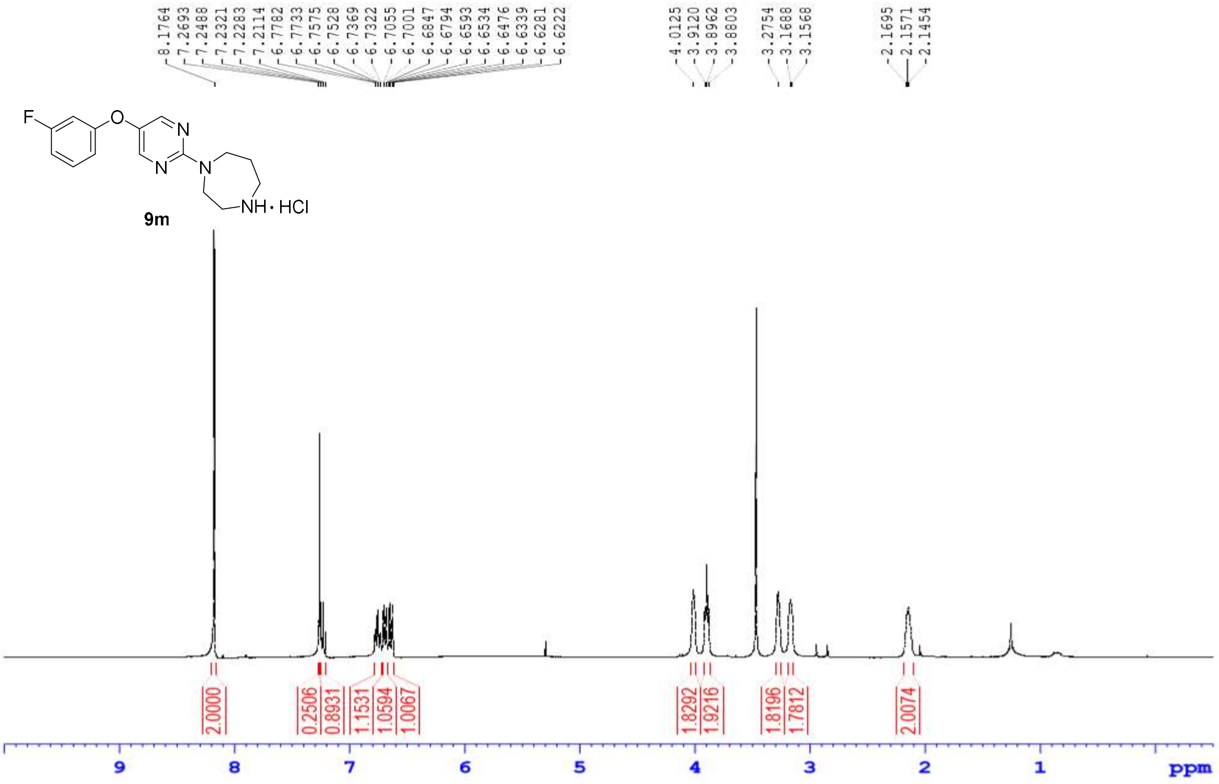

^{1}H NMR Spectrum of Compound **9m** (400 MHz)

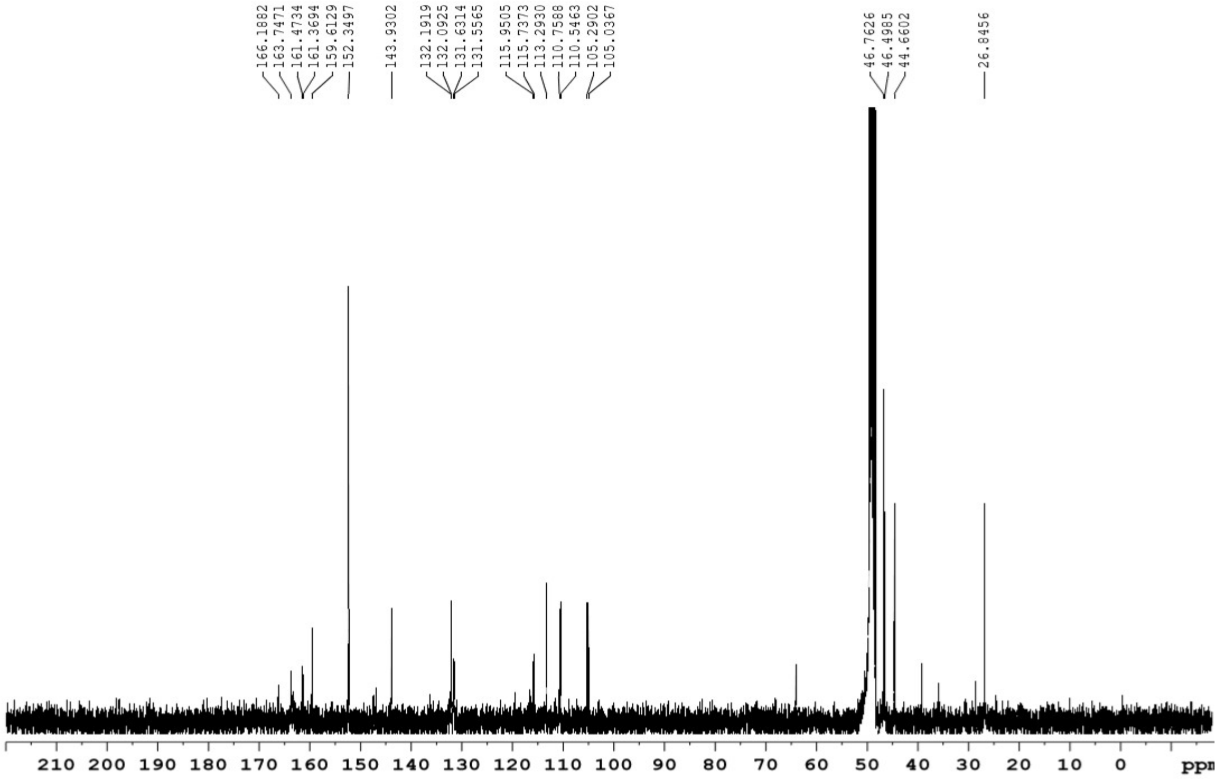

^{13}C NMR Spectrum of Compound **9m** (100 MHz)

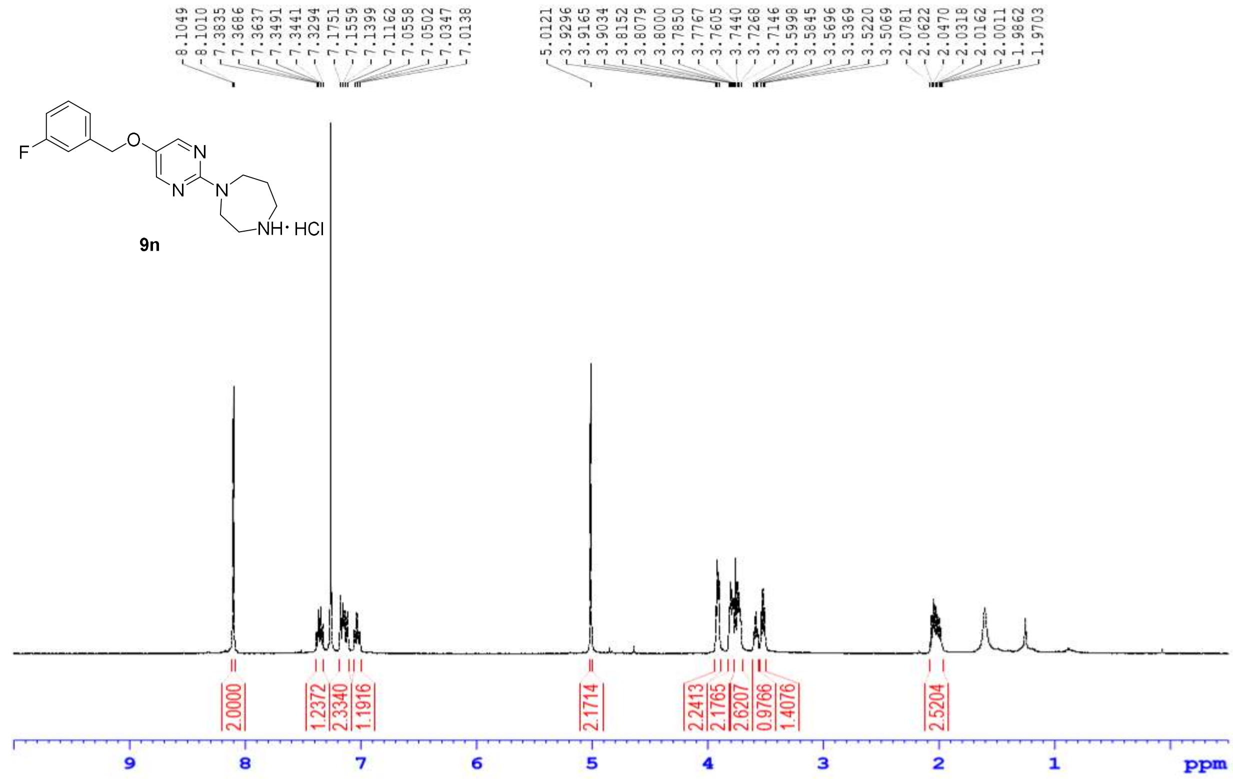

^{1}H NMR Spectrum of Compound **9n** (400 MHz)

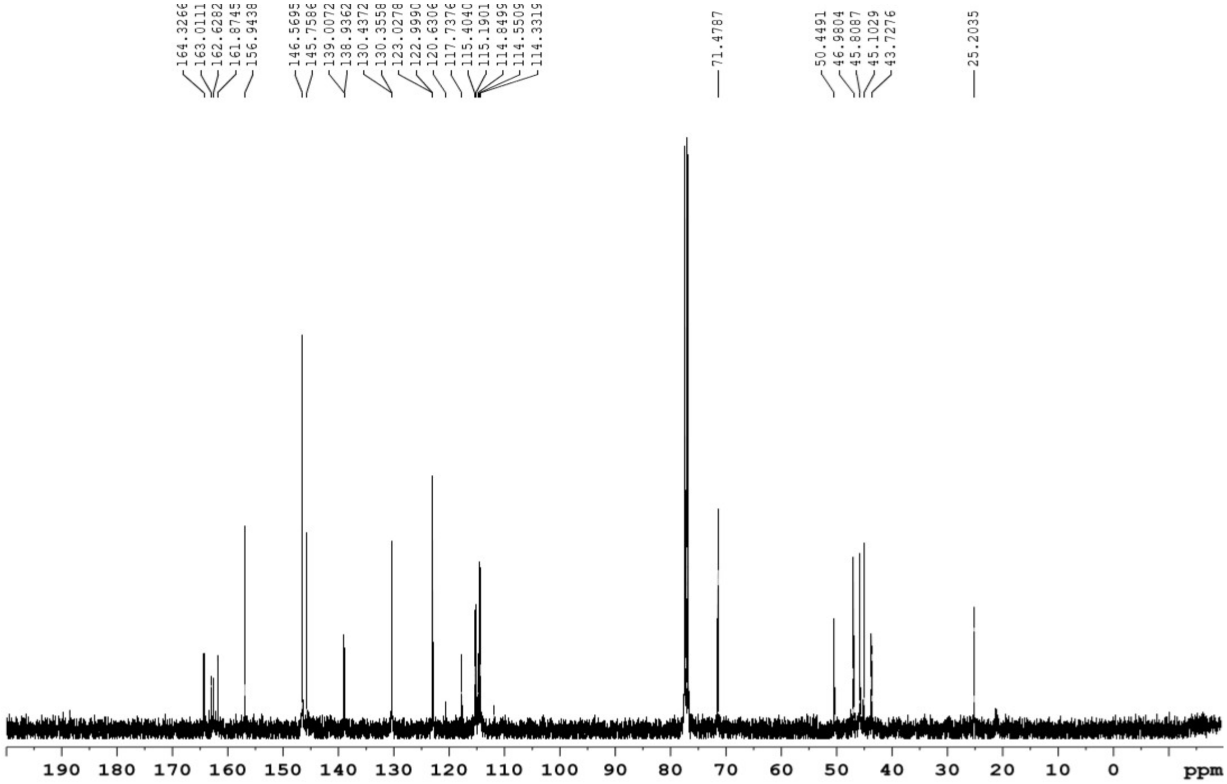

^{13}C NMR Spectrum of Compound **9n** (100 MHz)

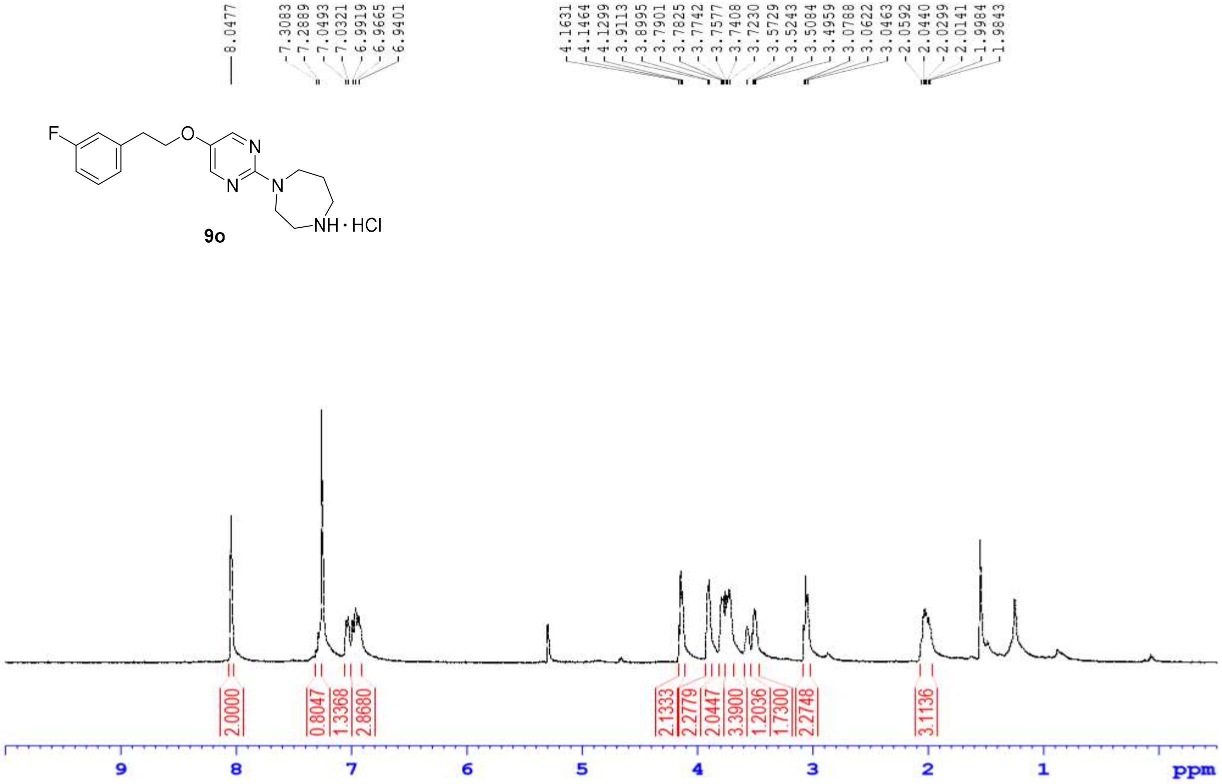

^{1}H NMR Spectrum of Compound **9o** (400 MHz)

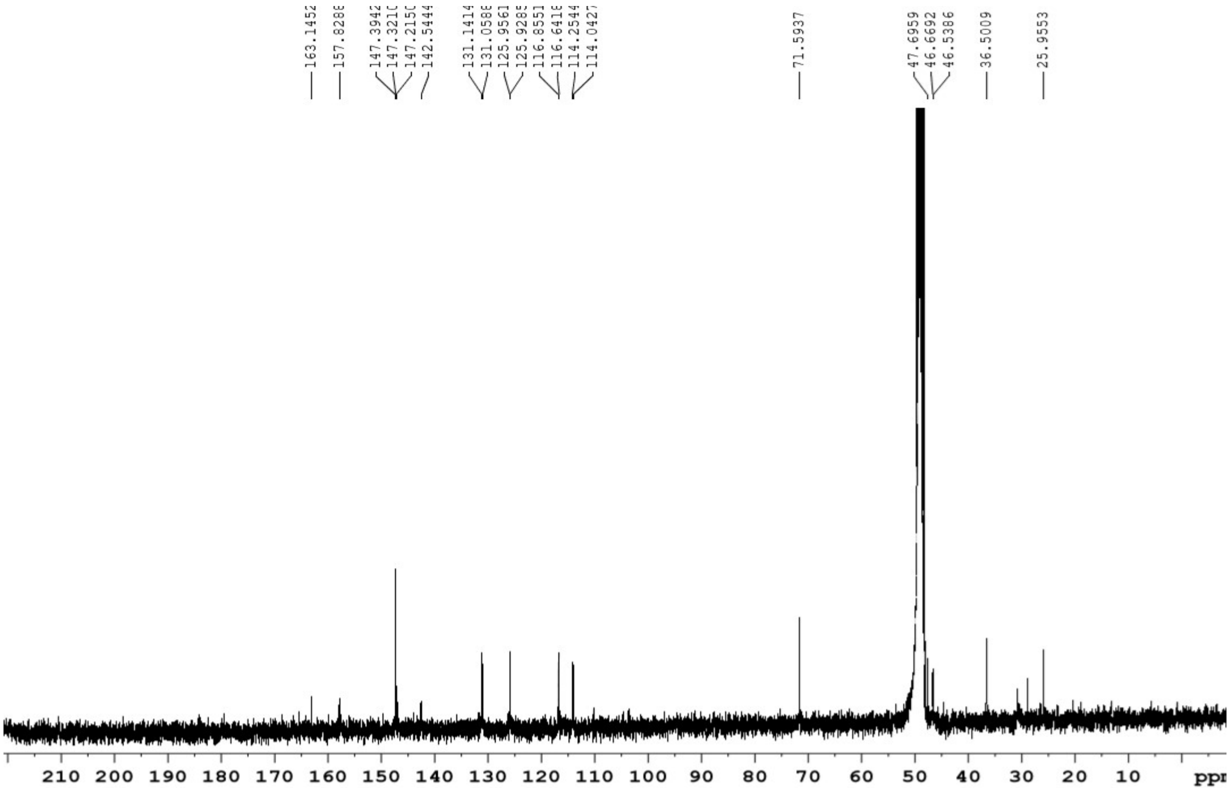

^{13}C NMR Spectrum of Compound **9o** (100 MHz)

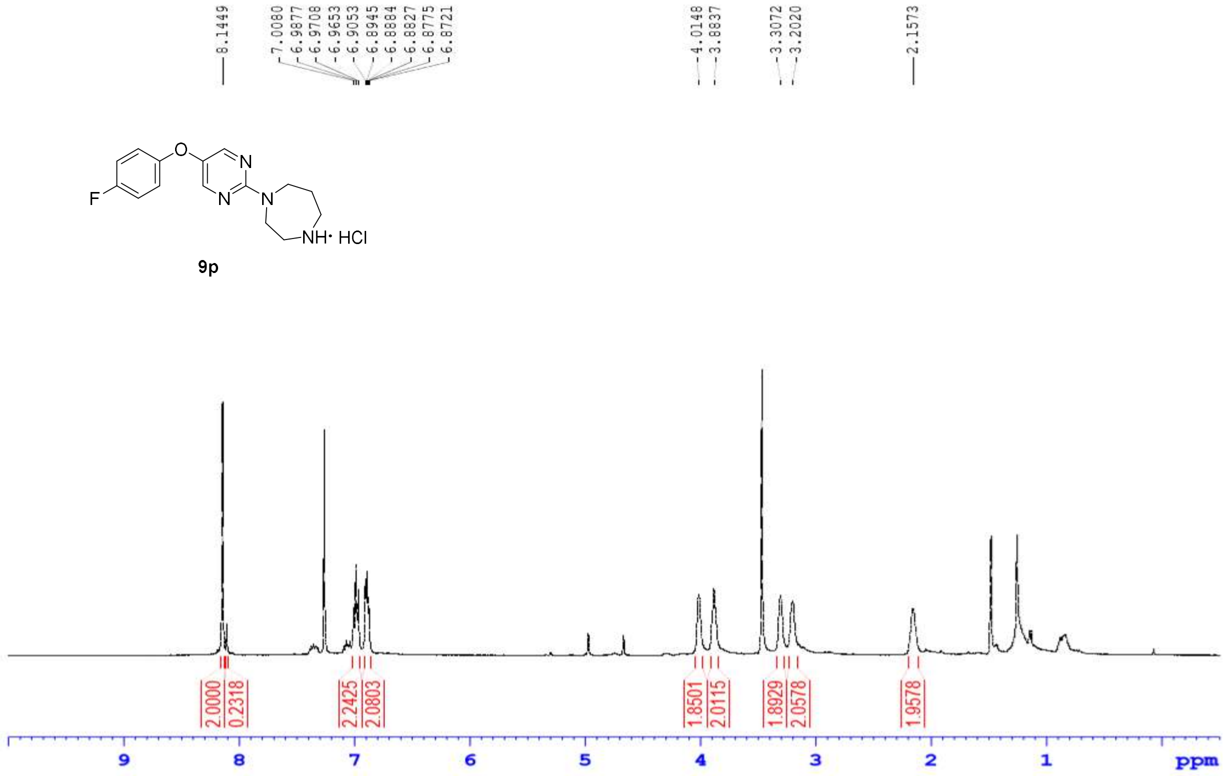

^{1}H NMR Spectrum of Compound **9p** (400 MHz)

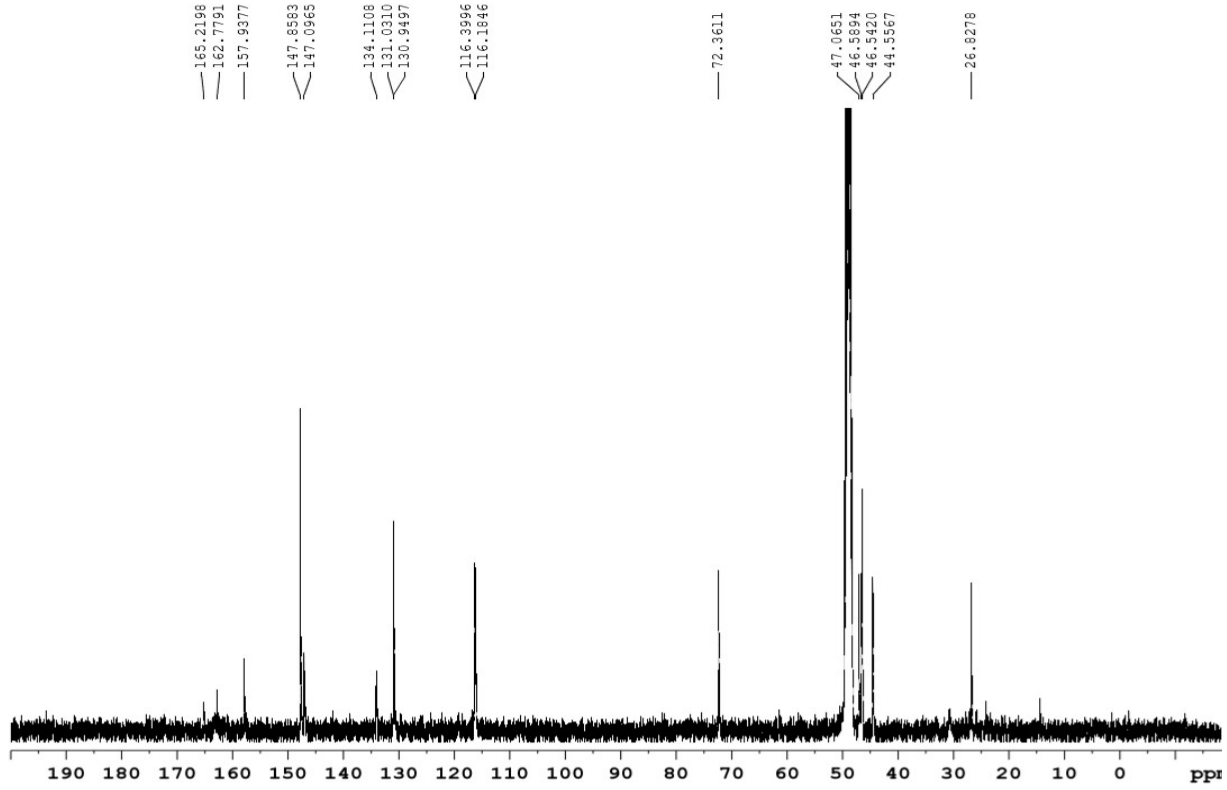

^{13}C NMR Spectrum of Compound **9p** (100 MHz)

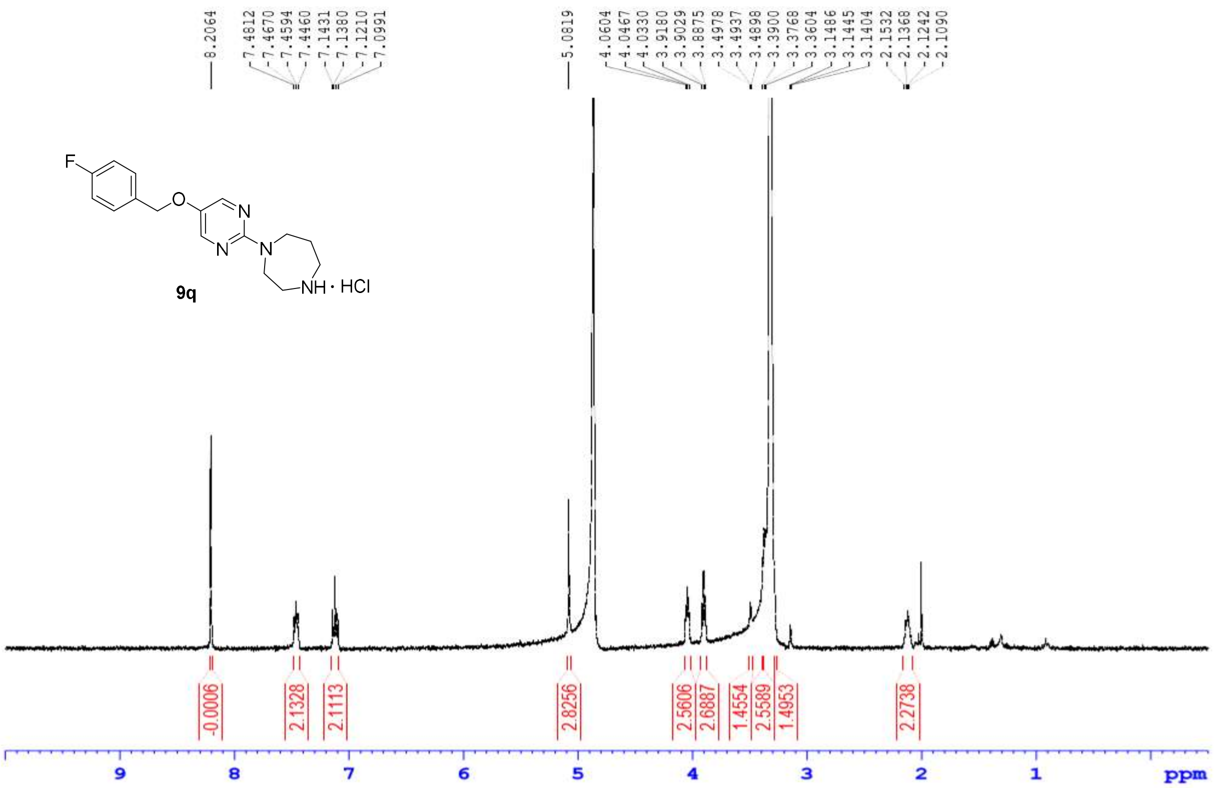

^{1}H NMR Spectrum of Compound **9q** (400 MHz)

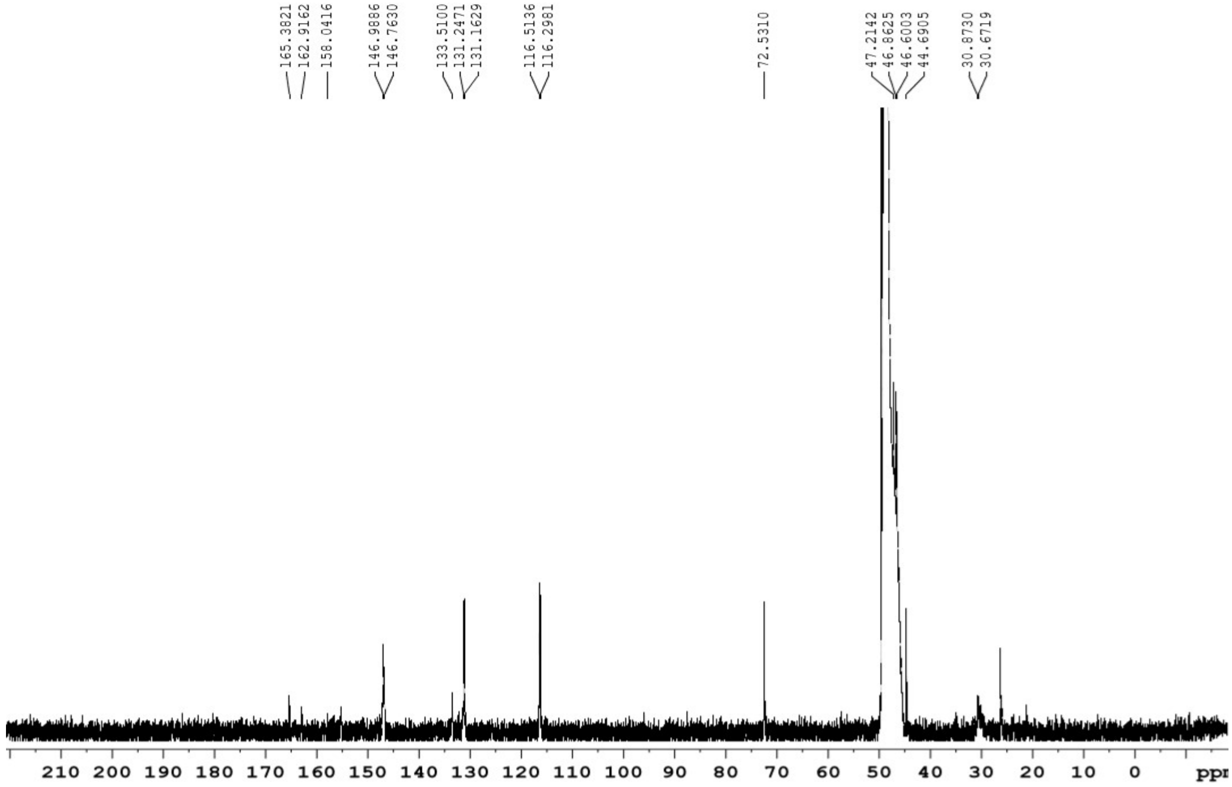

^{13}C NMR Spectrum of Compound **9q** (100 MHz)

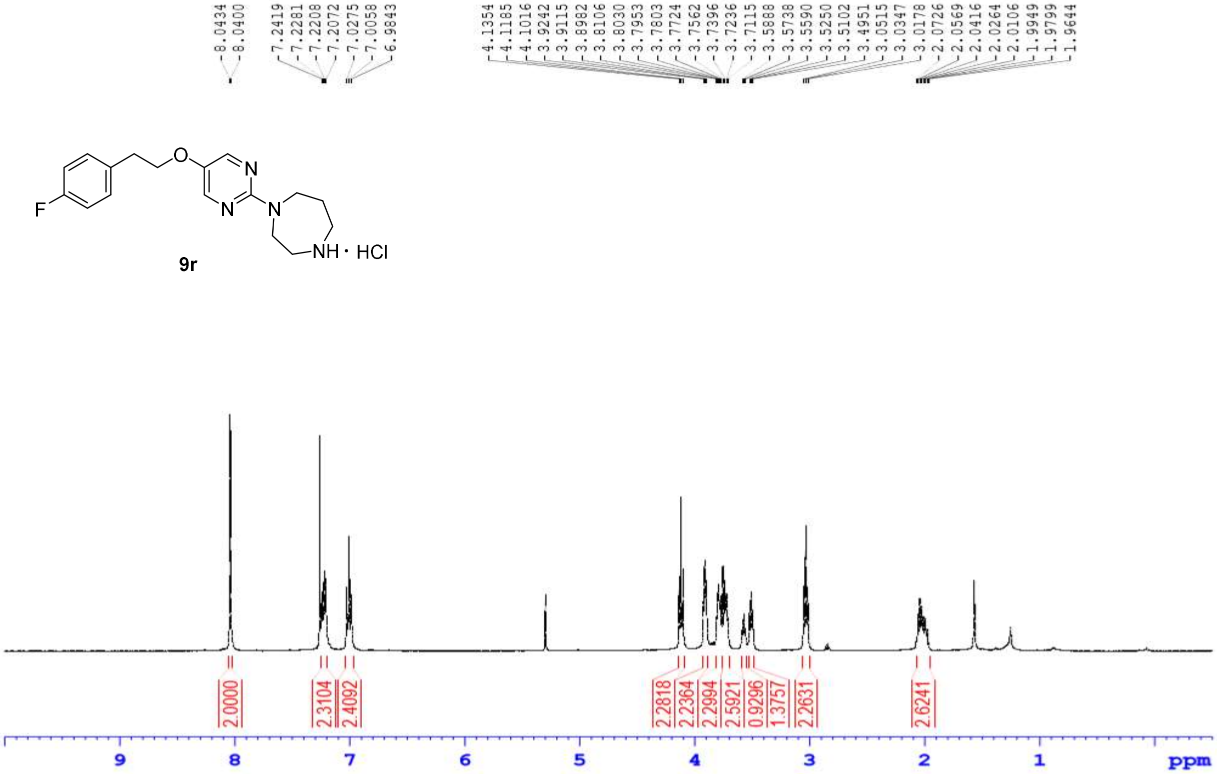

^{1}H NMR Spectrum of Compound **9r** (400 MHz)

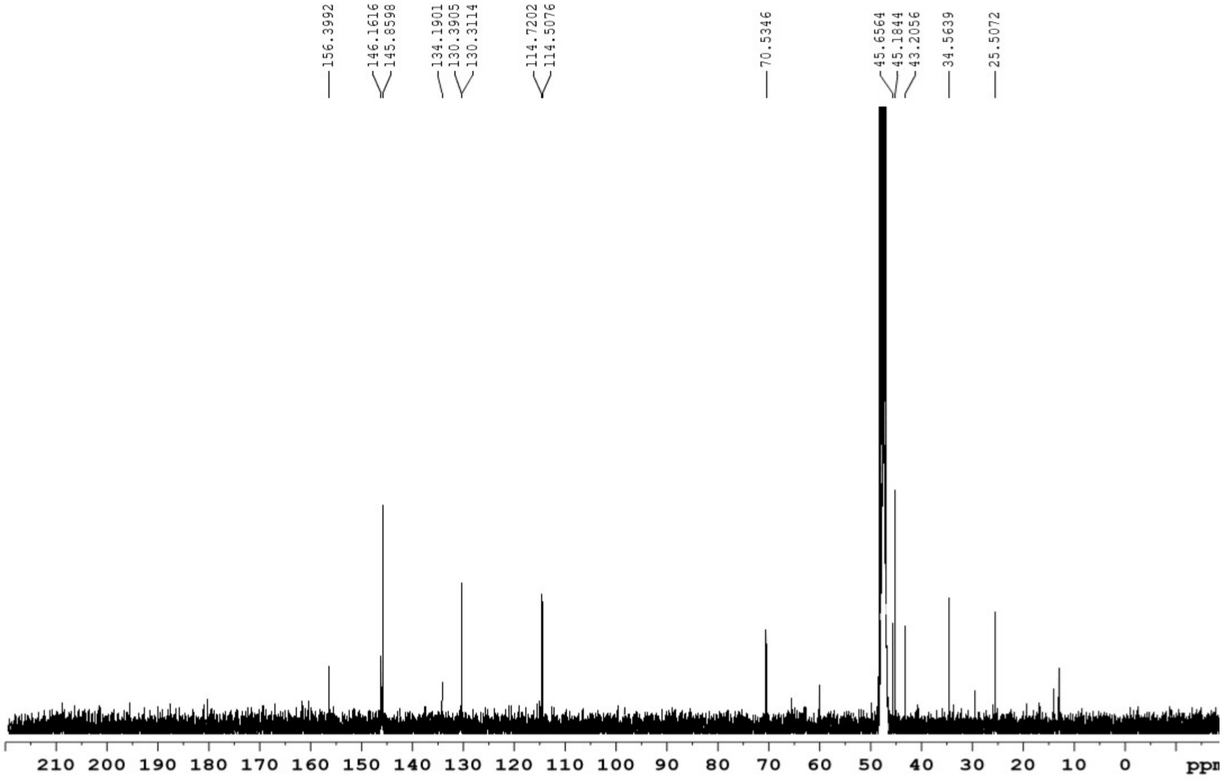

^{13}C NMR Spectrum of Compound **9r** (100 MHz)

2. NMR spectral data of 2,4-disubstituted pyrimidine **10a-10j,** and **20a/b**

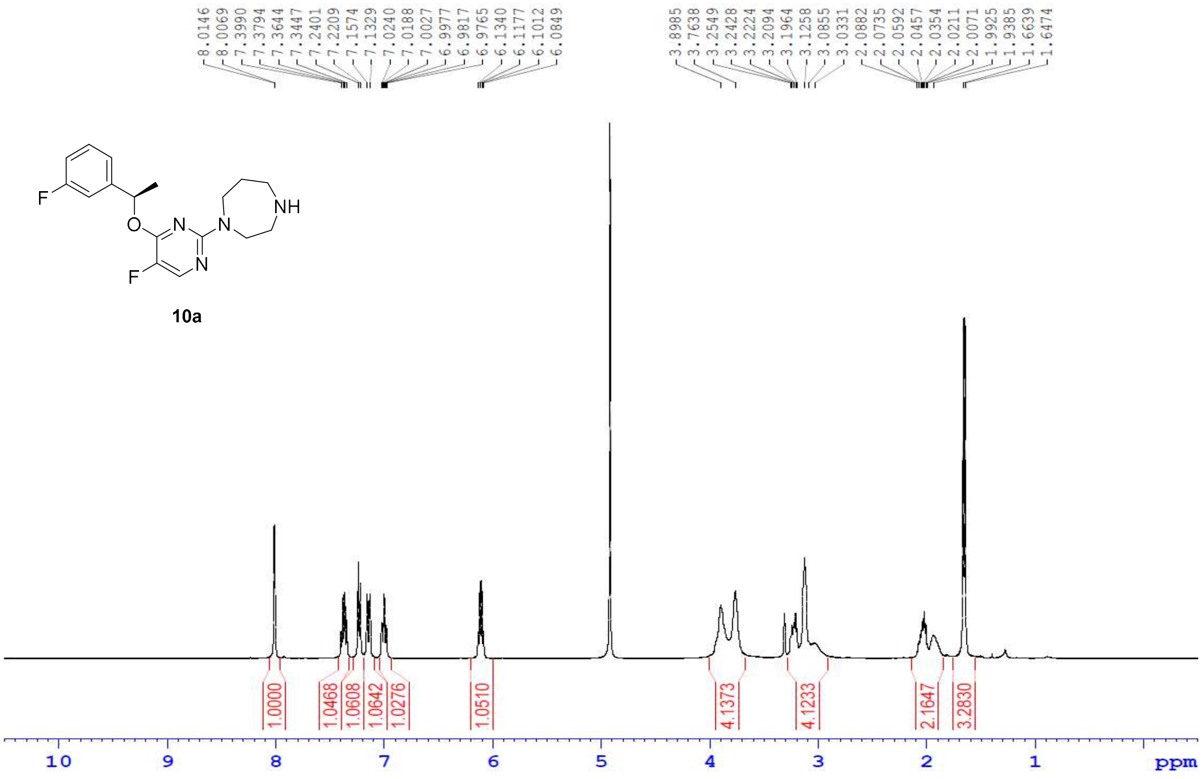

^{1}H NMR Spectrum of Compound **10a** (400 MHz)

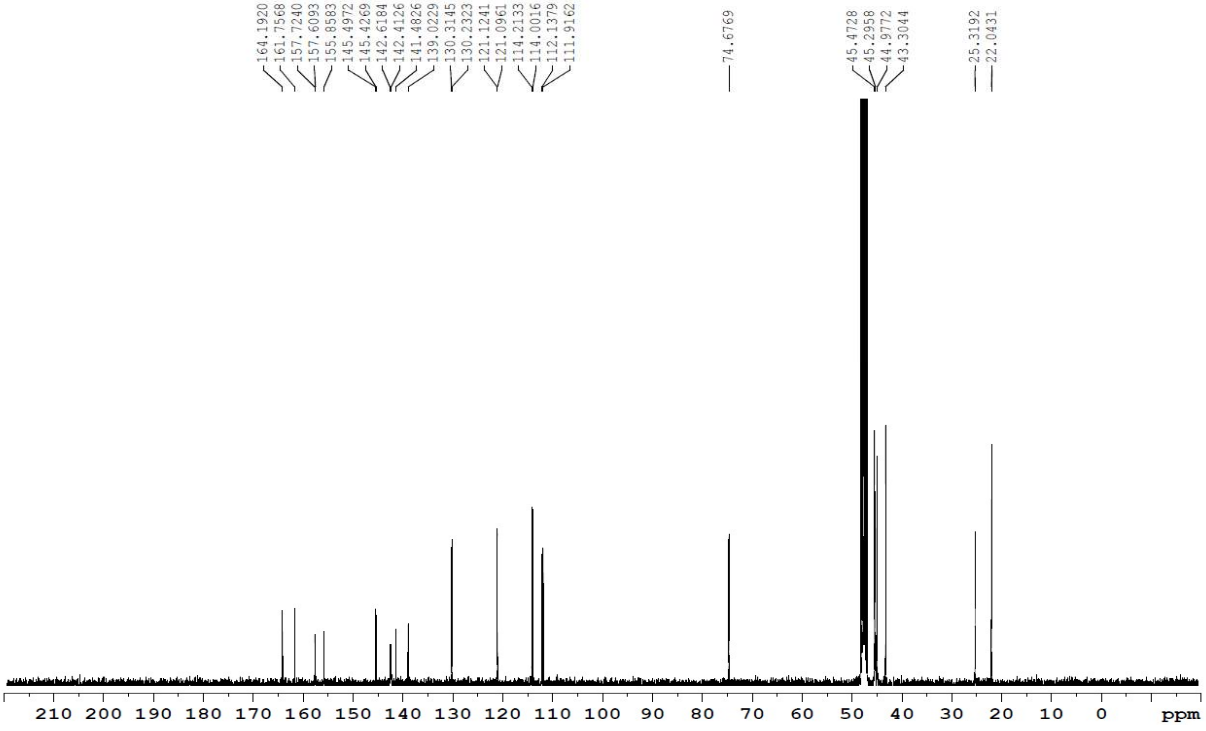

^{13}C NMR Spectrum of Compound **10a** (100 MHz)

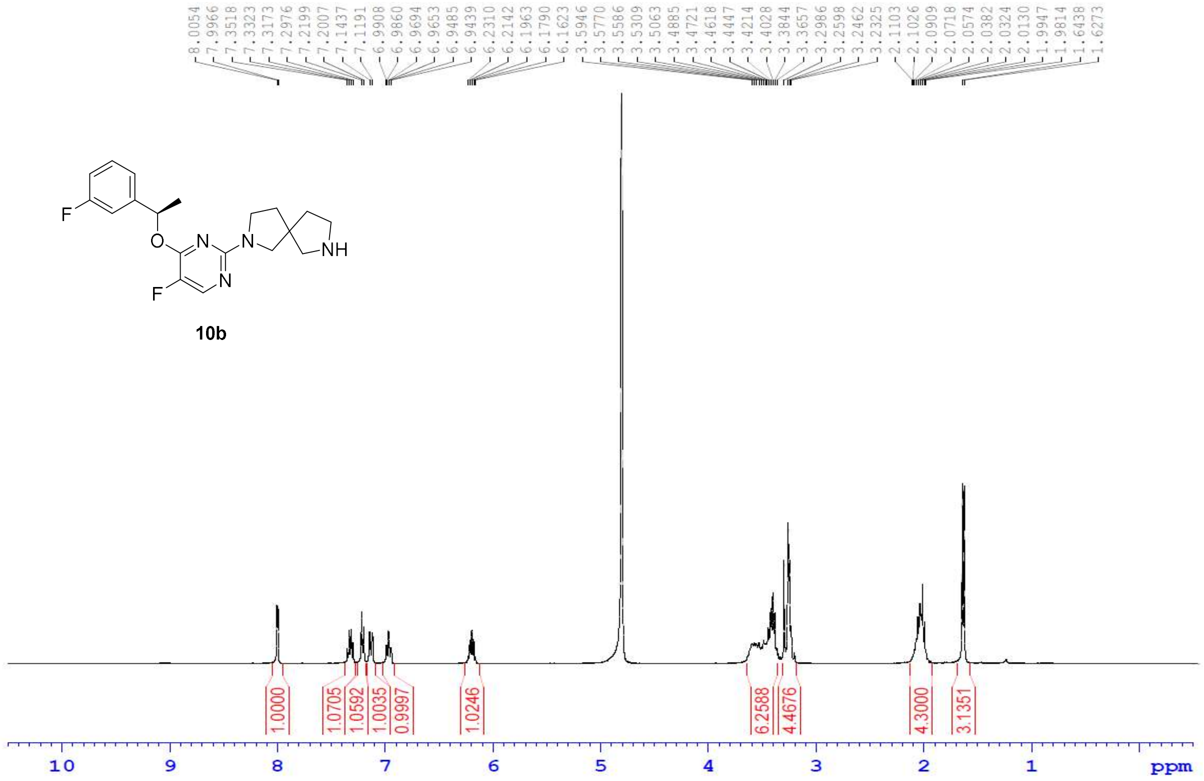

^{1}H NMR Spectrum of Compound **10b** (400 MHz)

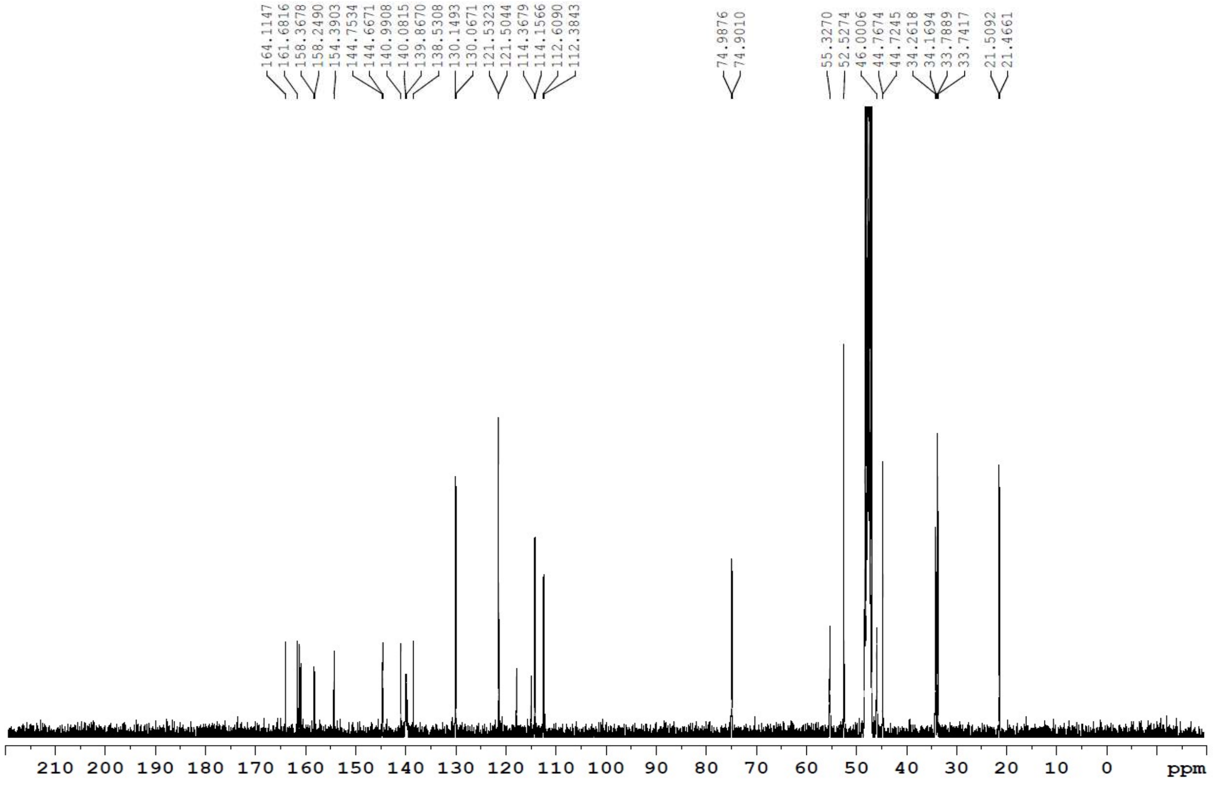

^{13}C NMR Spectrum of Compound **10b** (100 MHz)

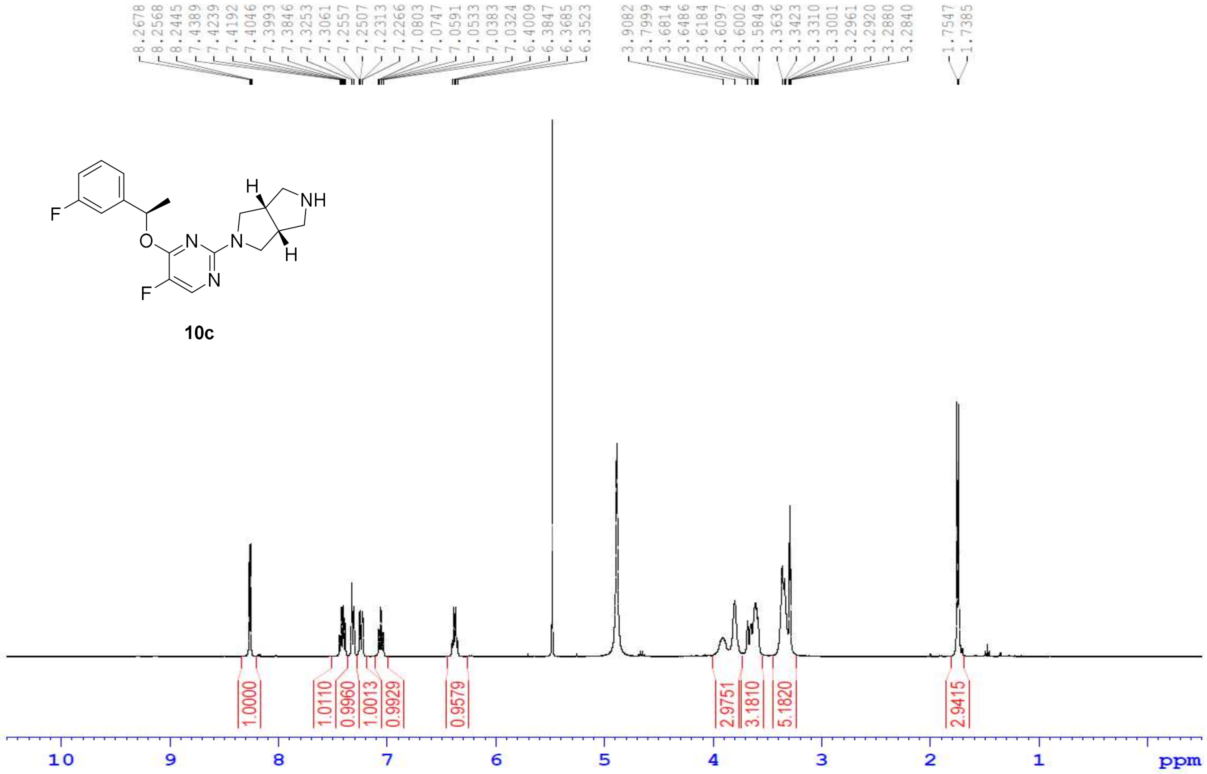

^{1}H NMR Spectrum of Compound **10c** (400 MHz)

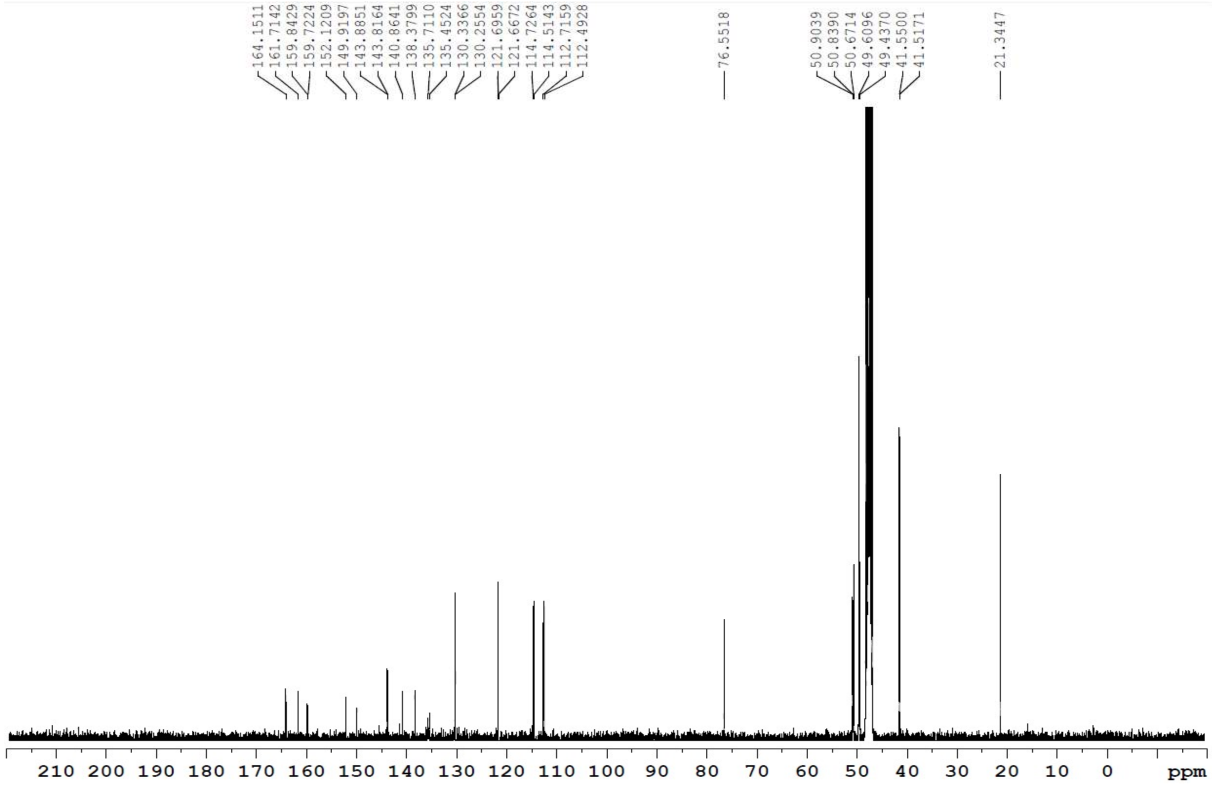

^{13}C NMR Spectrum of Compound **10c** (100 MHz)

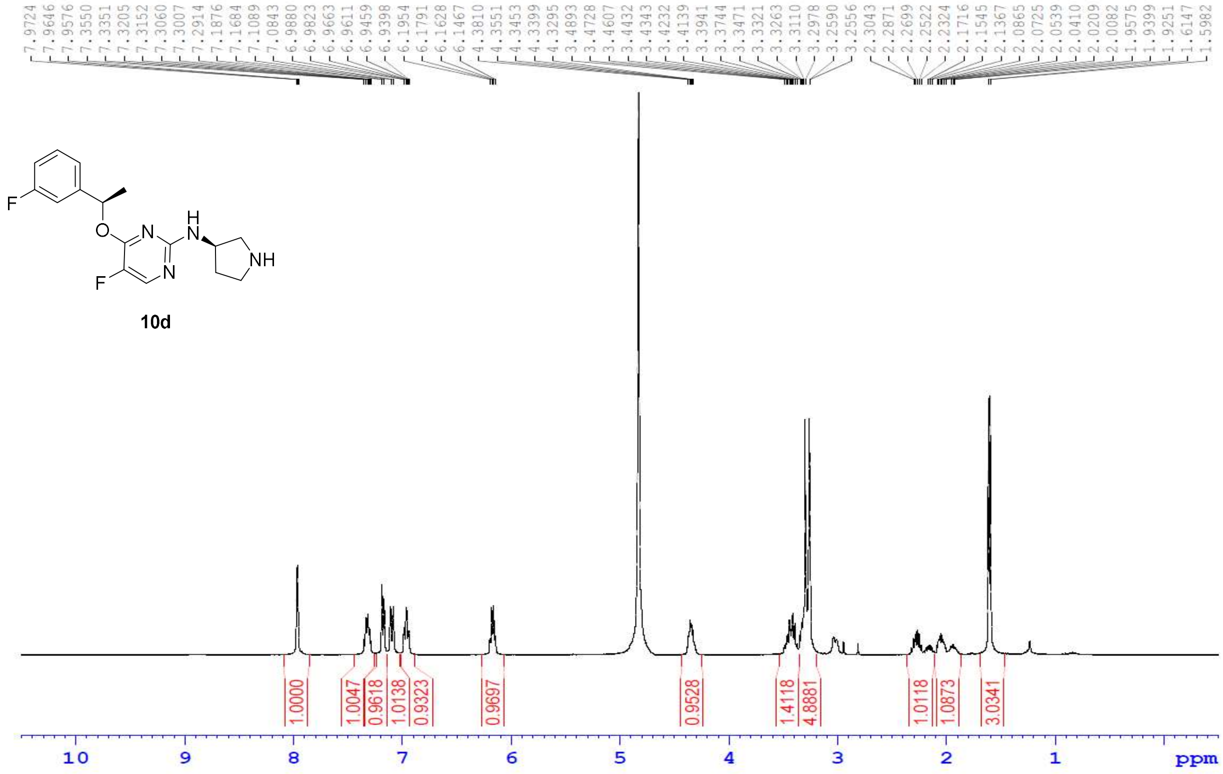

^{1}H NMR Spectrum of Compound **10d** (400 MHz)

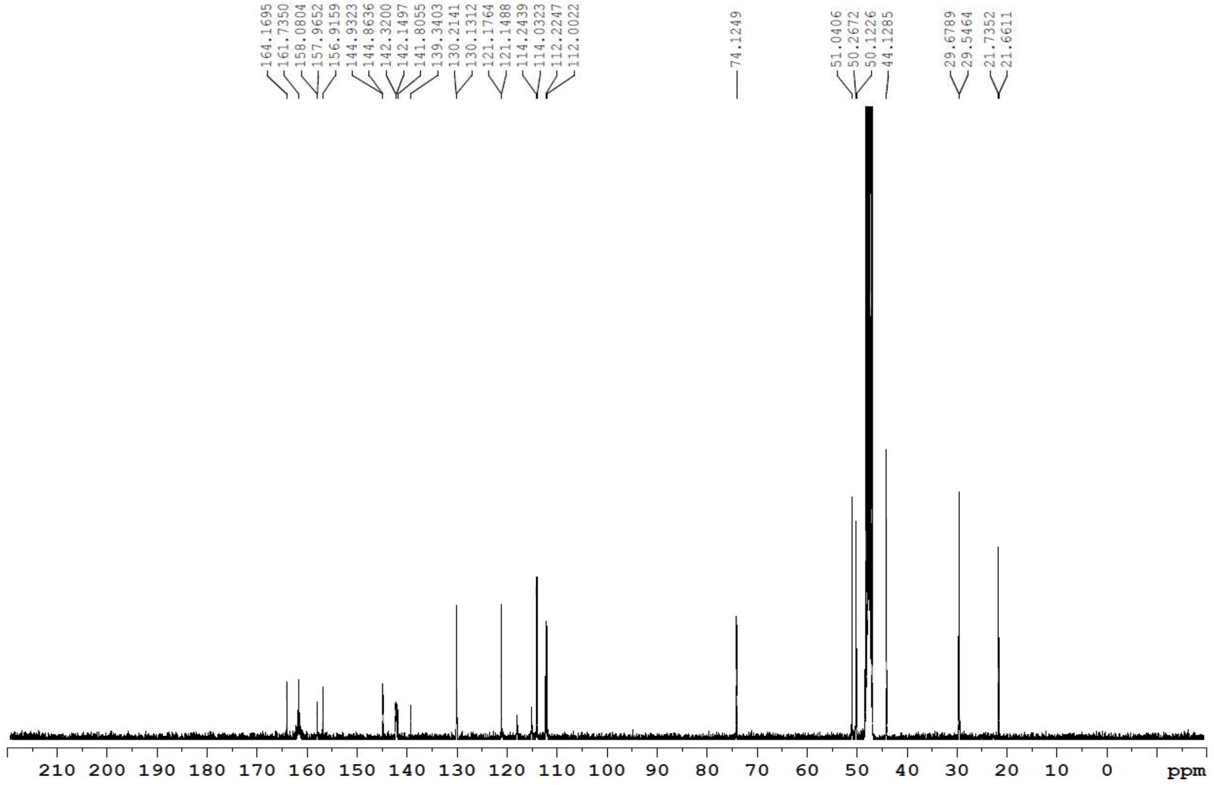

^{13}C NMR Spectrum of Compound **10d** (100 MHz)

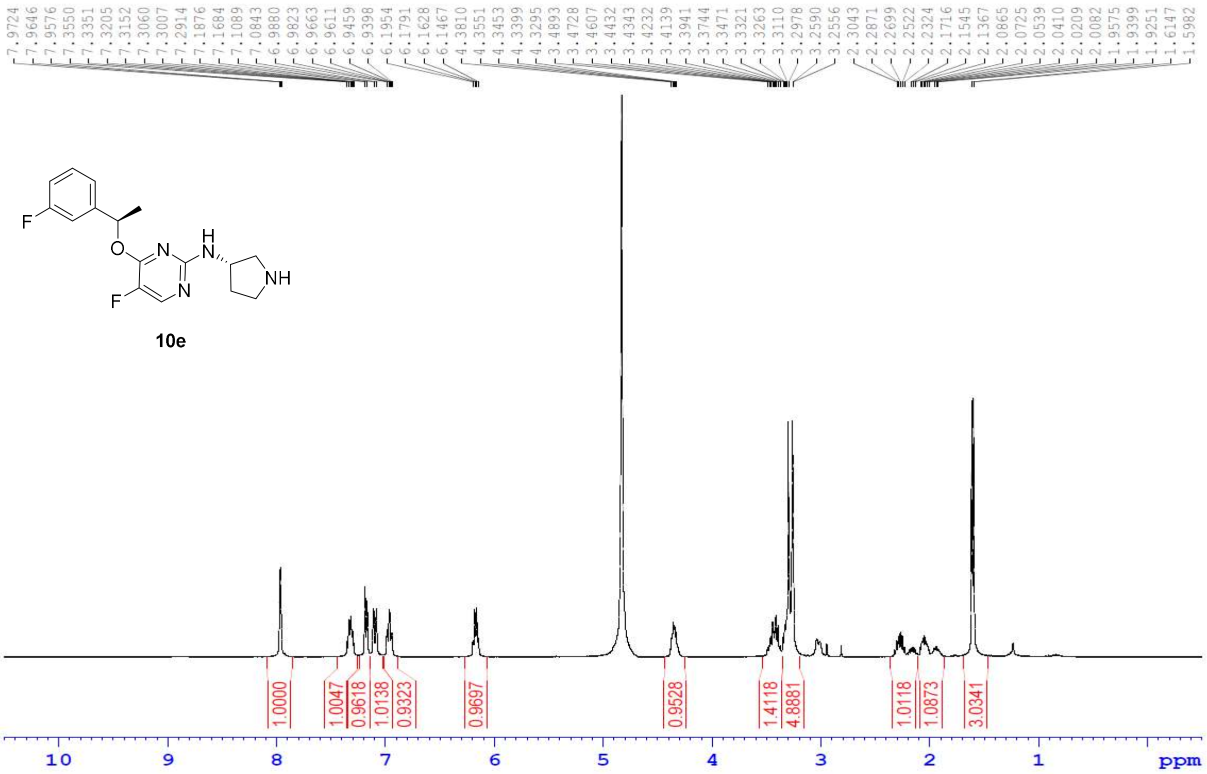

^{1}H NMR Spectrum of Compound **10e** (400 MHz)

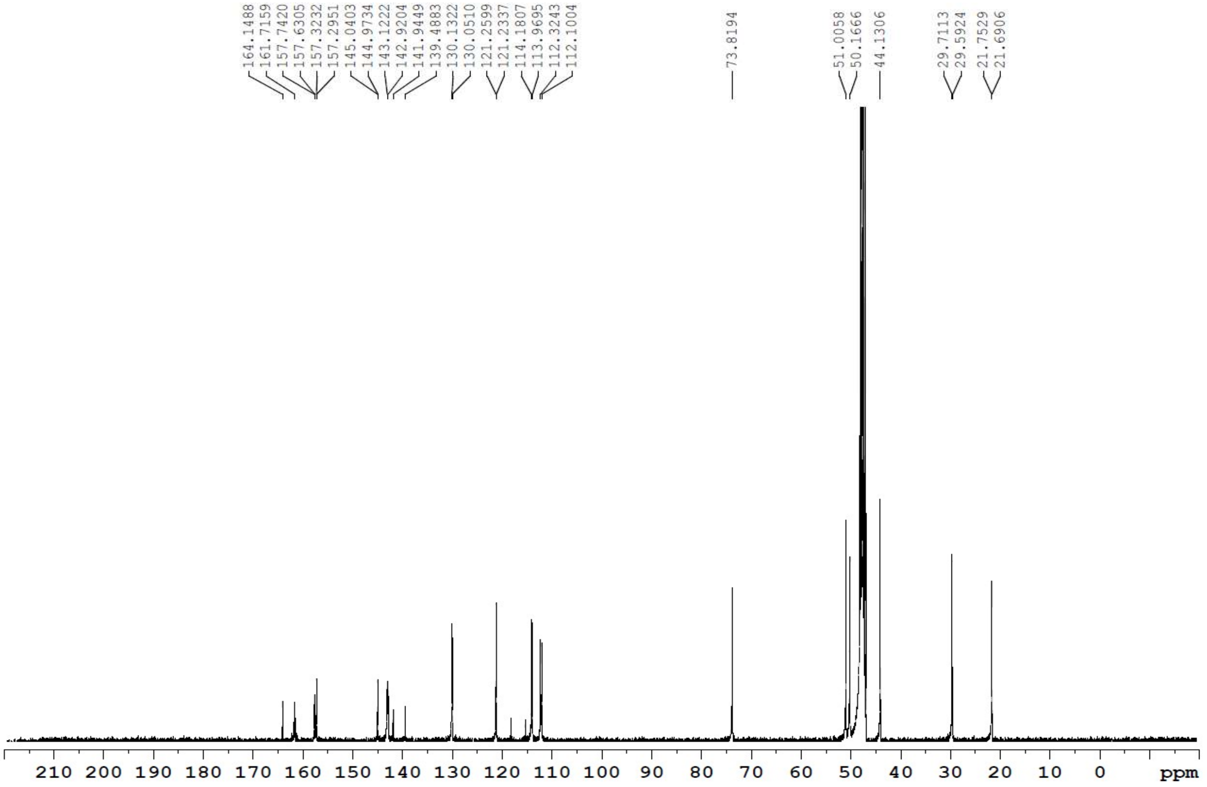

^{13}C NMR Spectrum of Compound **10e** (100 MHz)

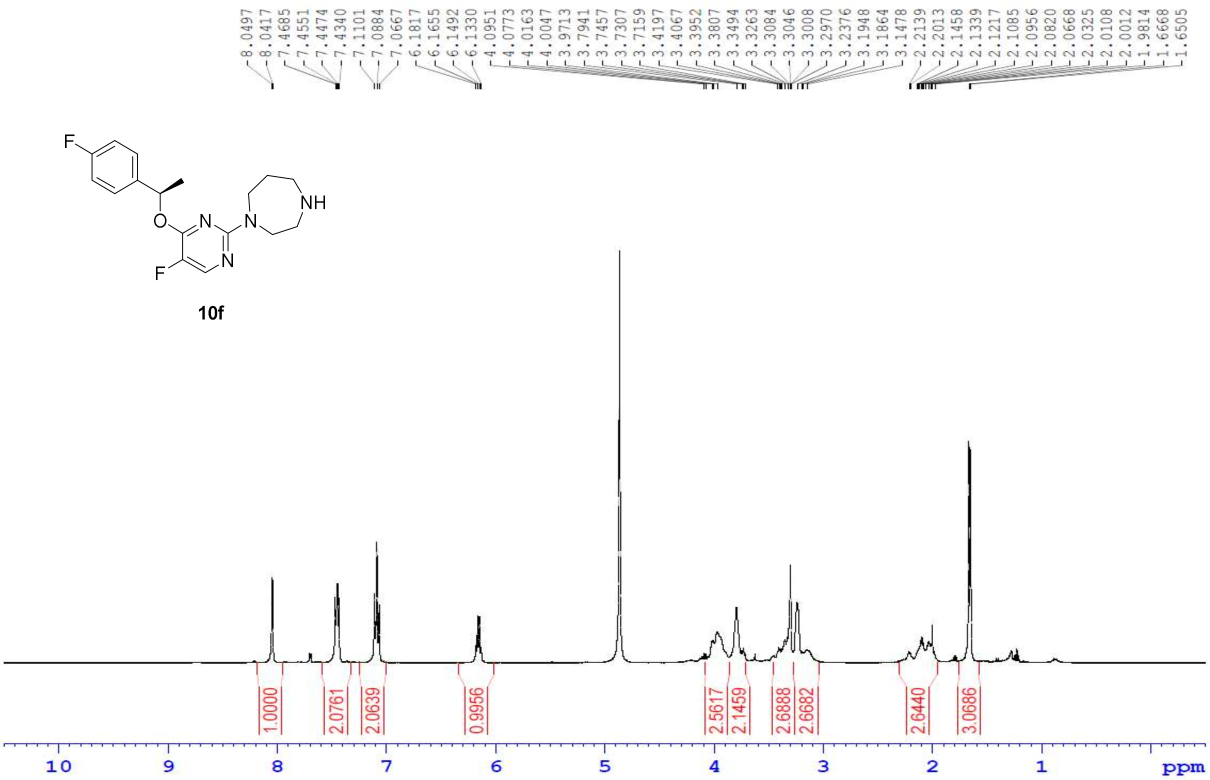

^{1}H NMR Spectrum of Compound **10f** (400 MHz)

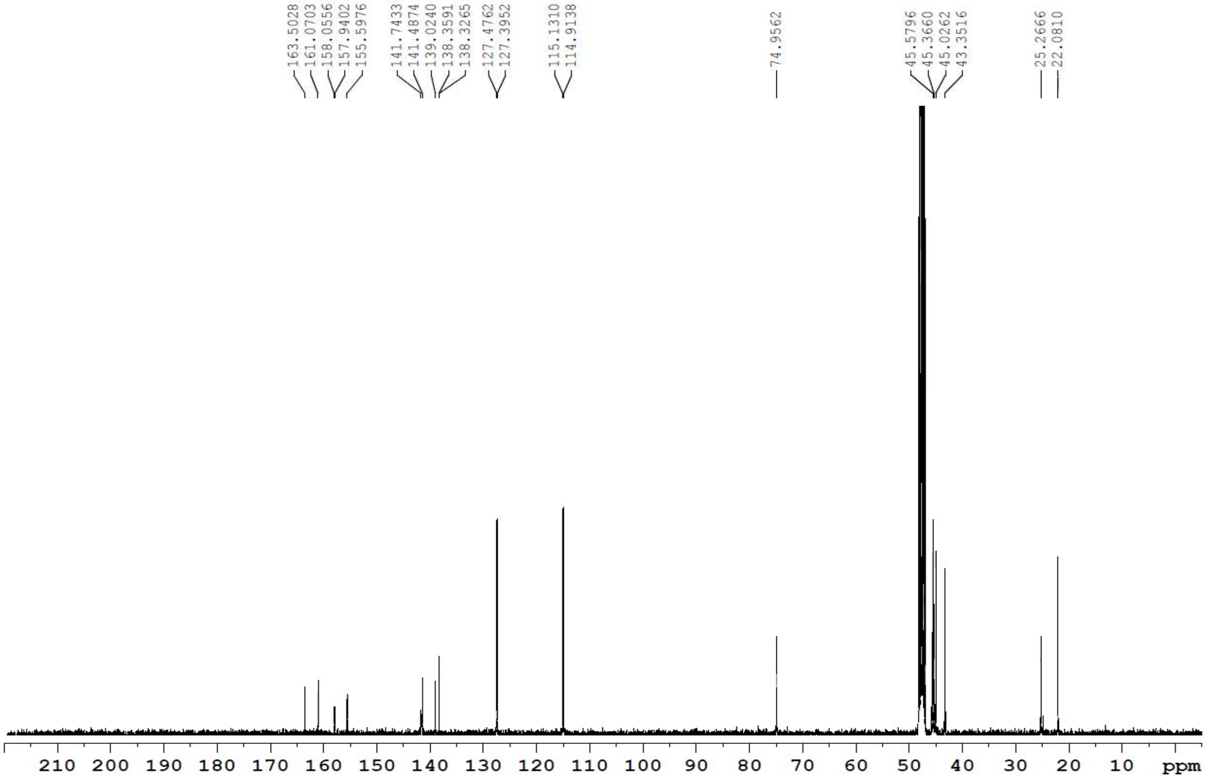

^{13}C NMR Spectrum of Compound **10f** (100 MHz)

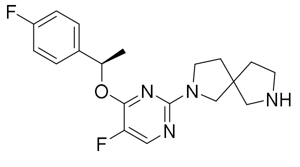

10g

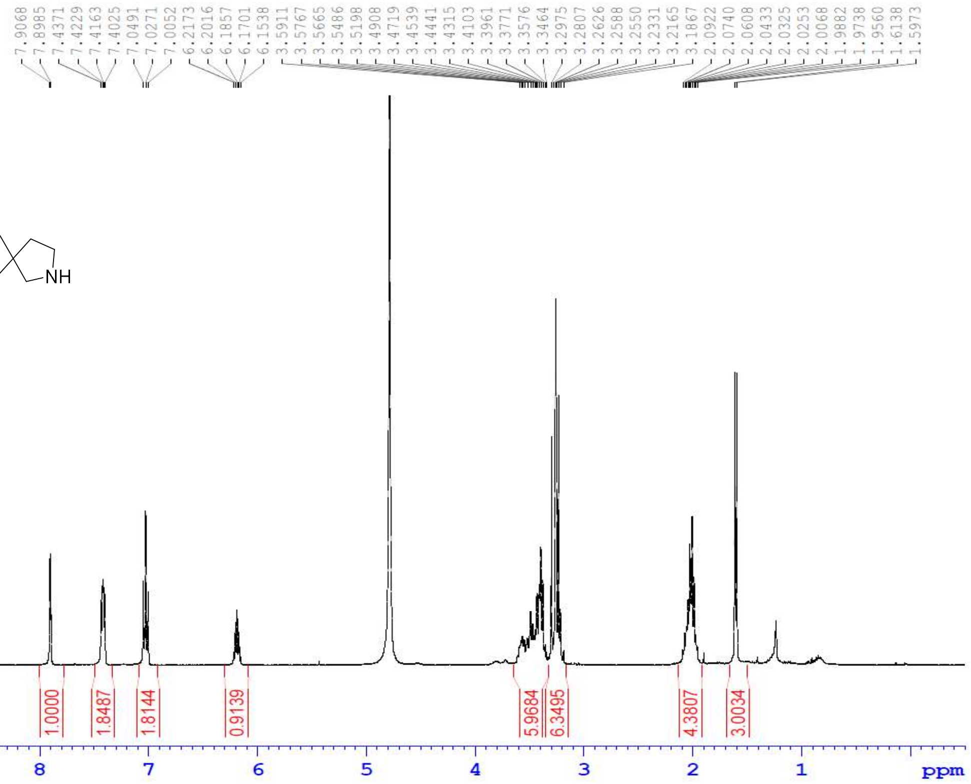

^{1}H NMR Spectrum of Compound **10g** (400 MHz)

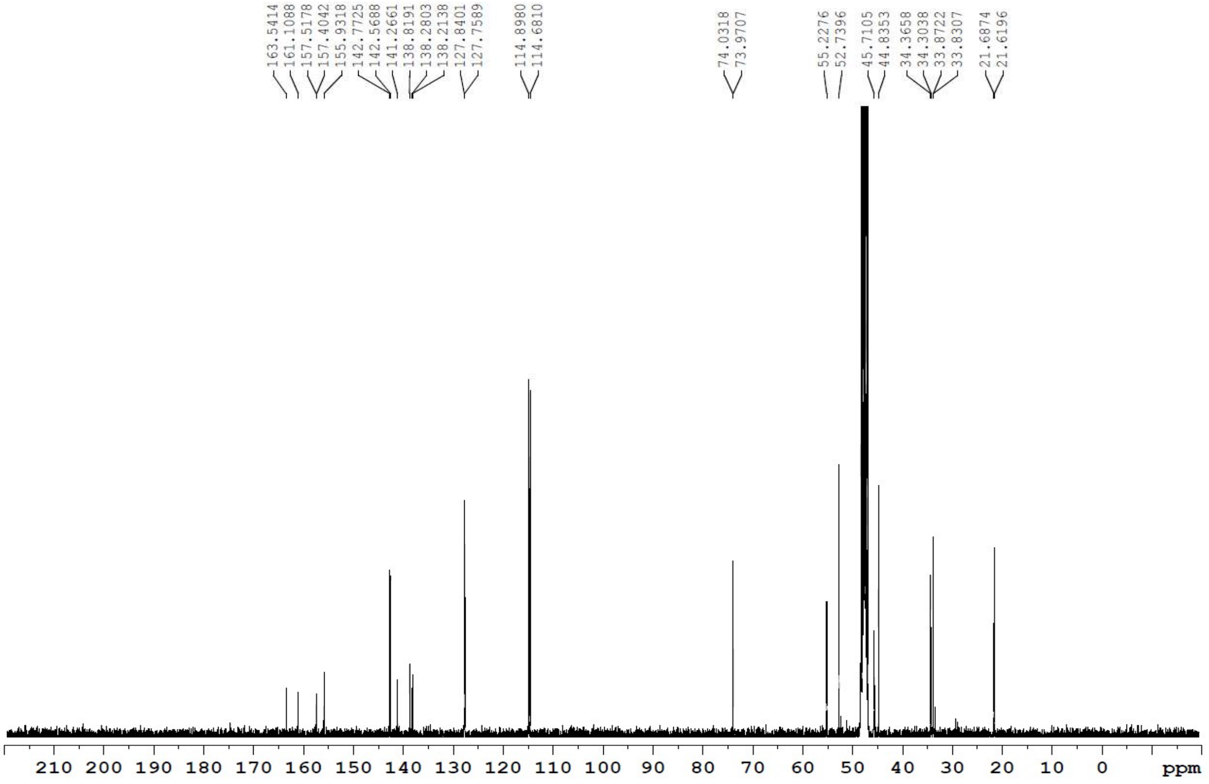

^{13}C NMR Spectrum of Compound **10g** (100 MHz)

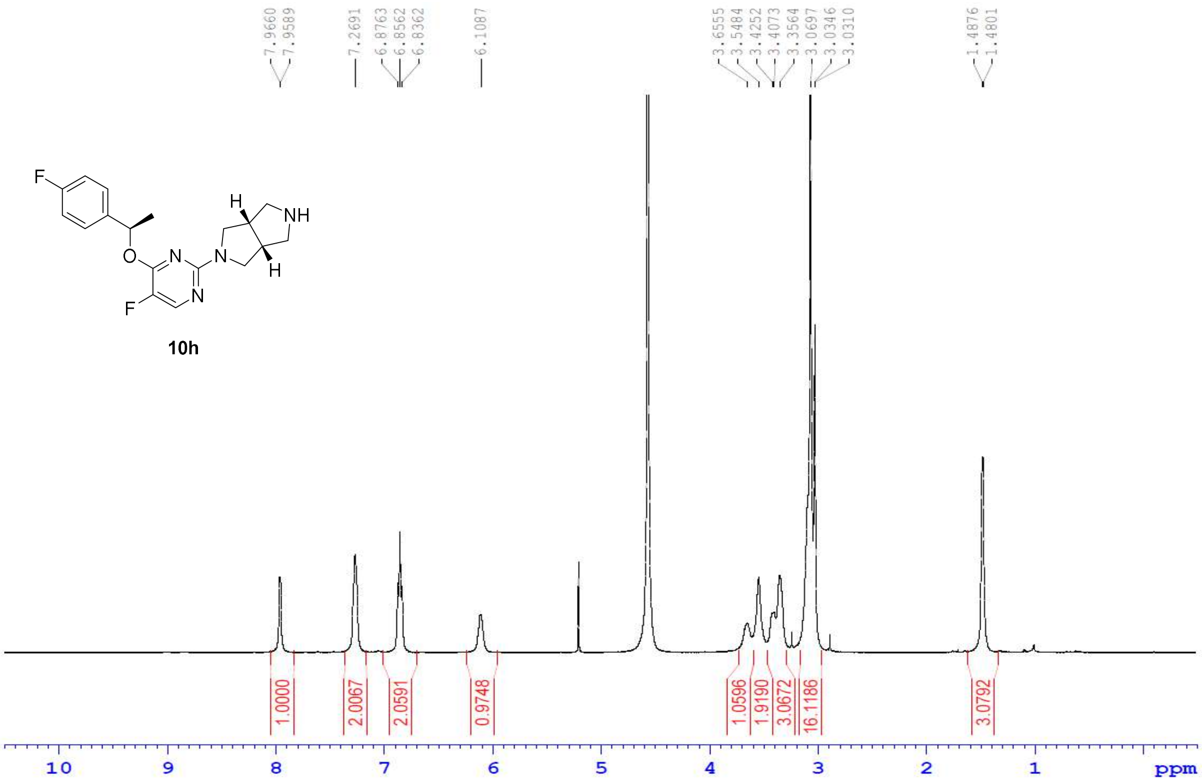

^{1}H NMR Spectrum of Compound **10h** (400 MHz)

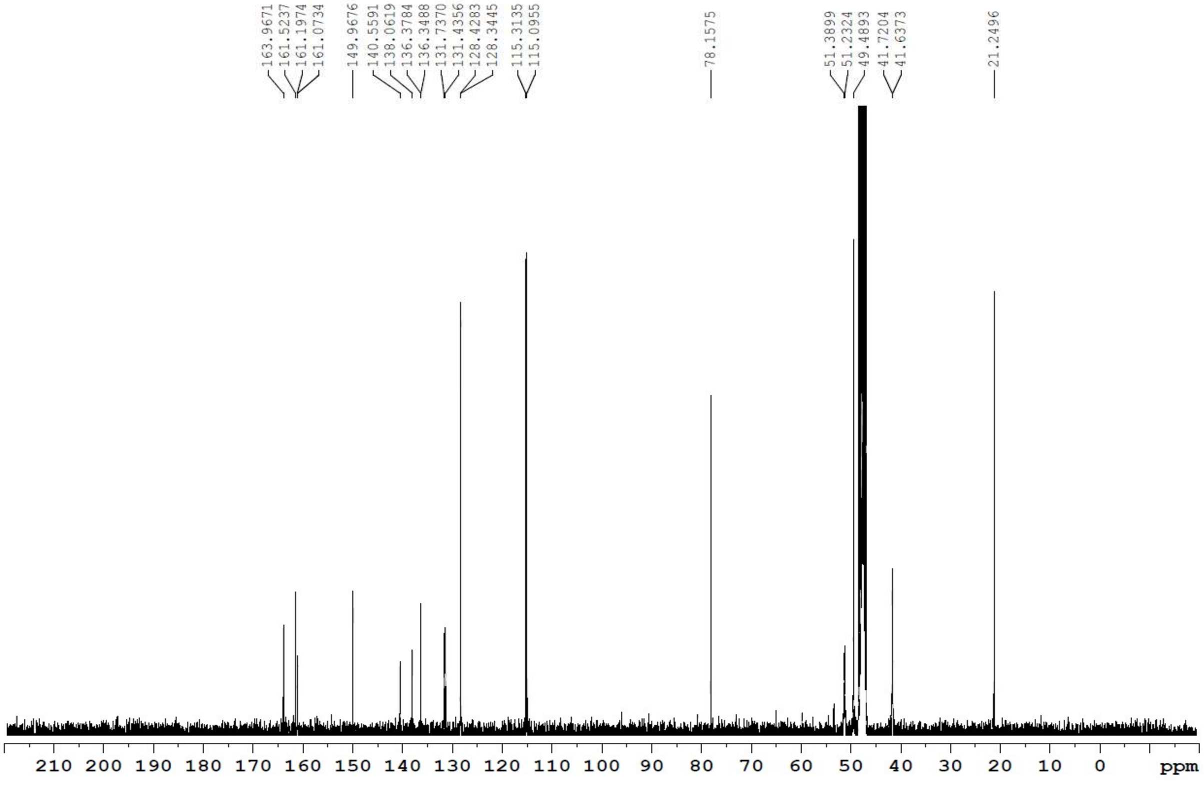

^{13}C NMR Spectrum of Compound **10h** (100 MHz)

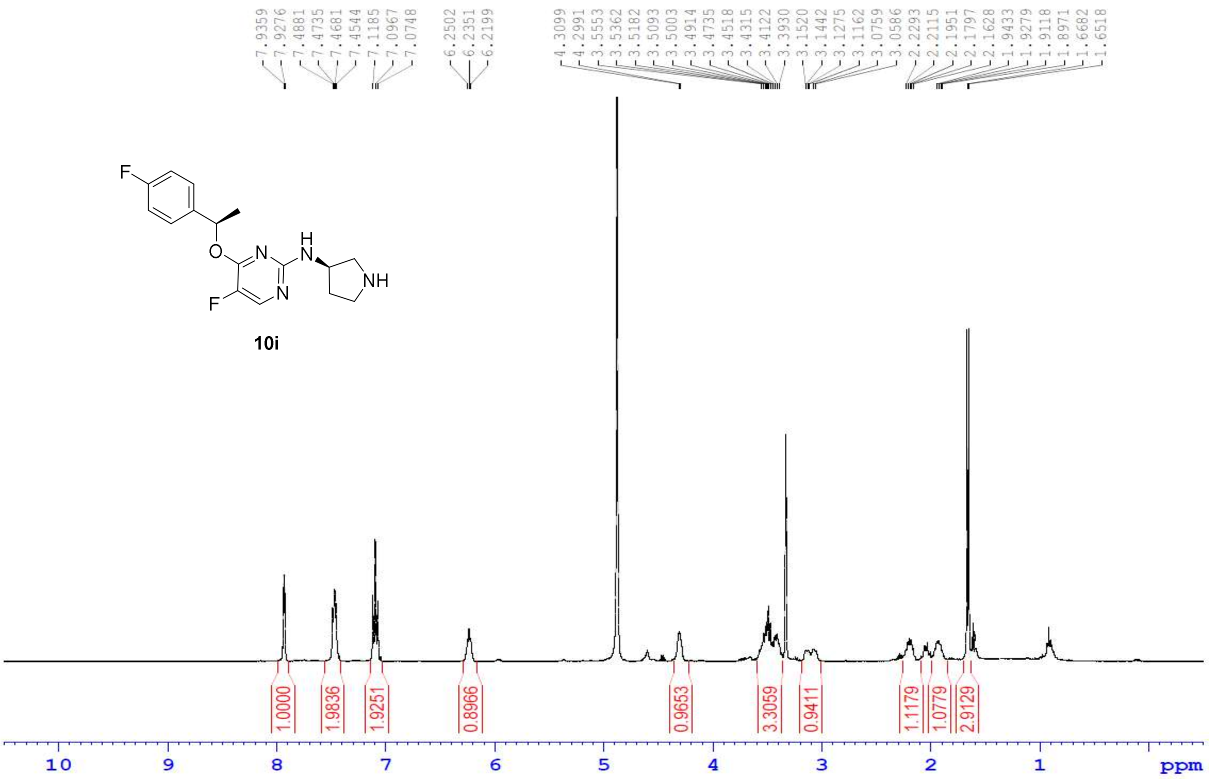

^{1}H NMR Spectrum of Compound **10i** (400 MHz)

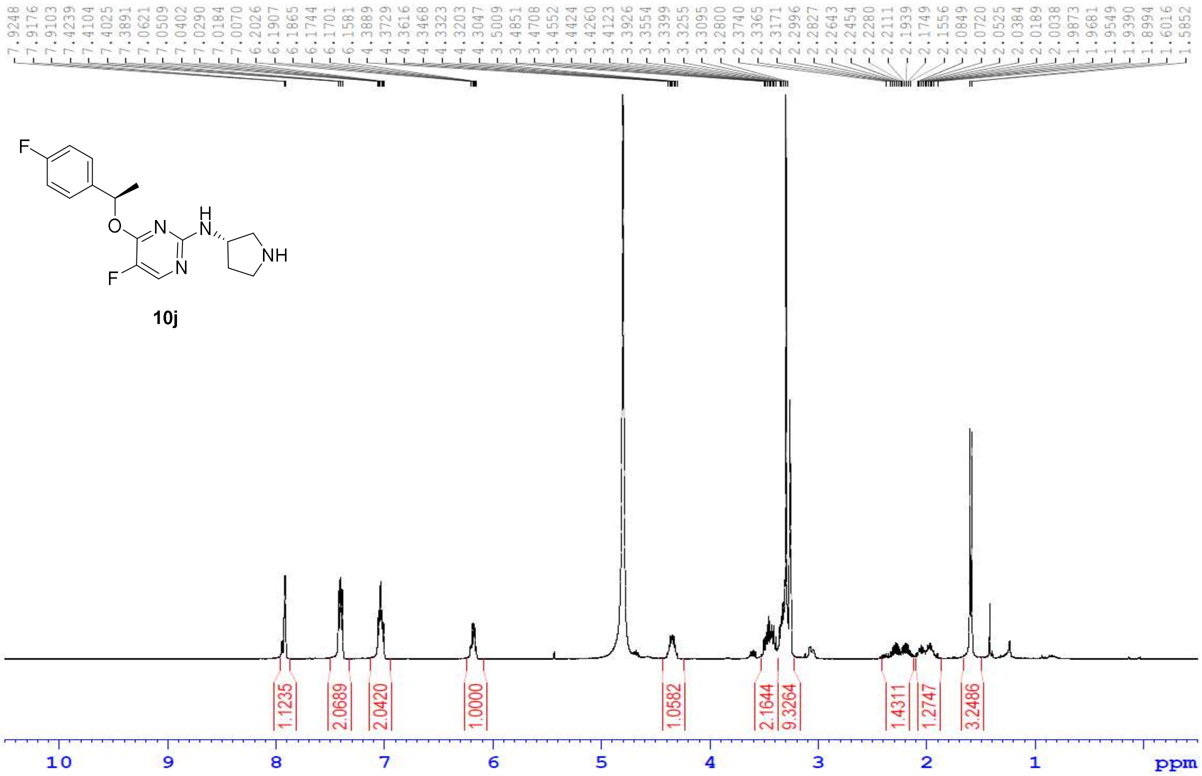

^{1}H NMR Spectrum of Compound **10j** (400 MHz)

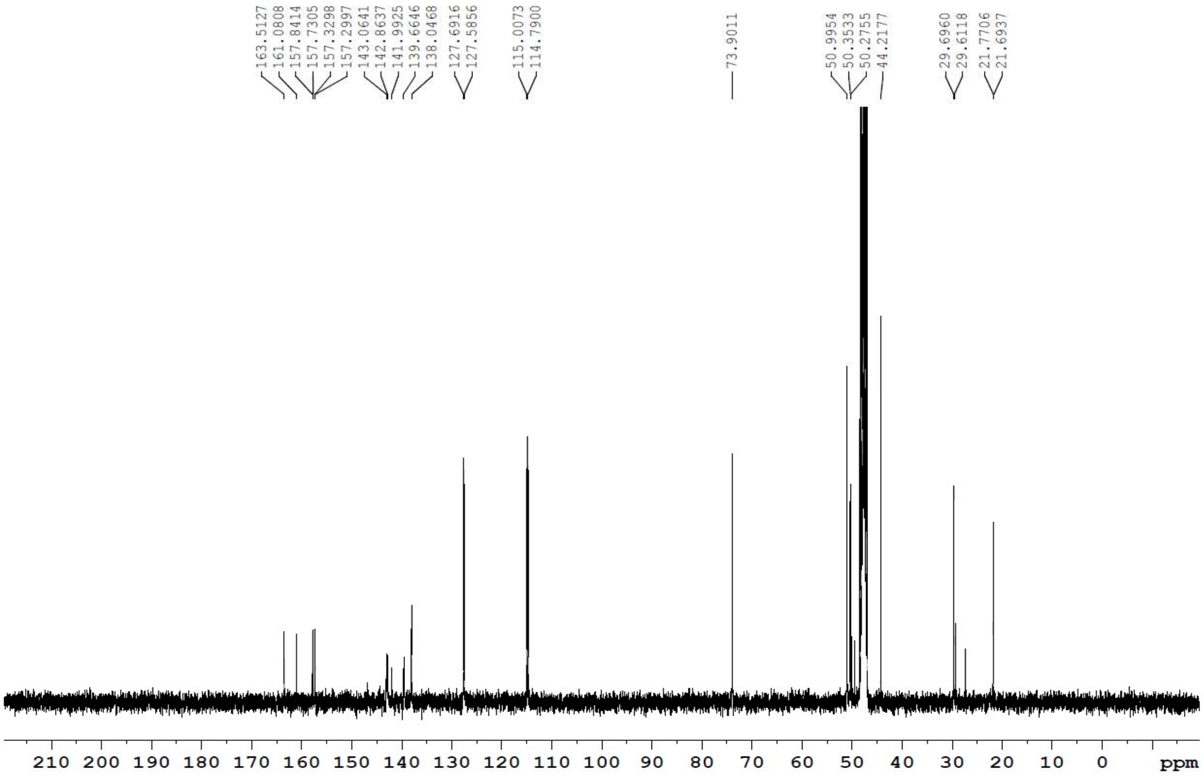

^{13}C NMR Spectrum of Compound **10j** (100 MHz)

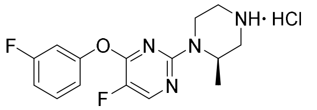

20a

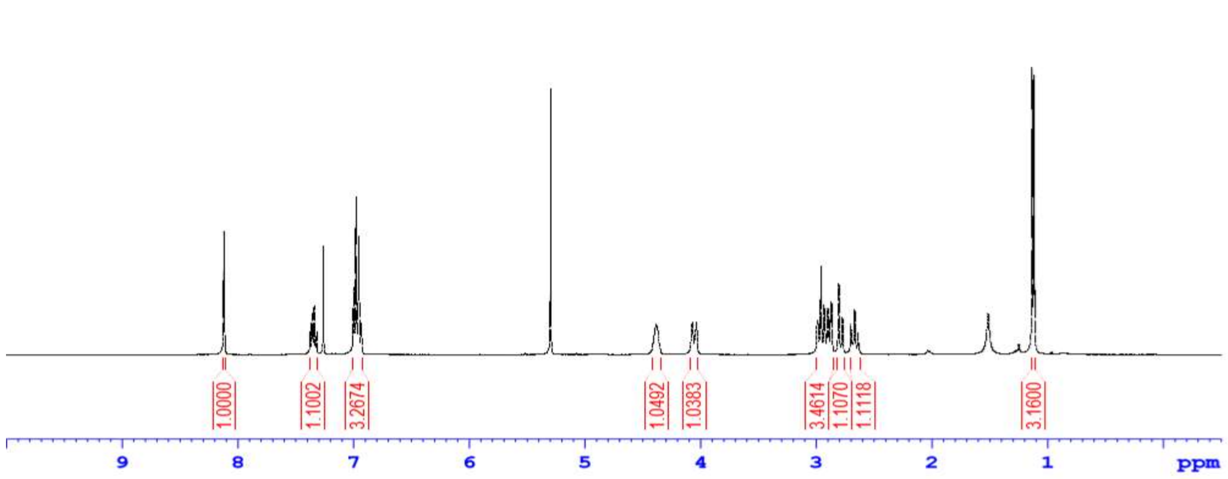

^{1}H NMR Spectrum of Compound **20a** (400 MHz)

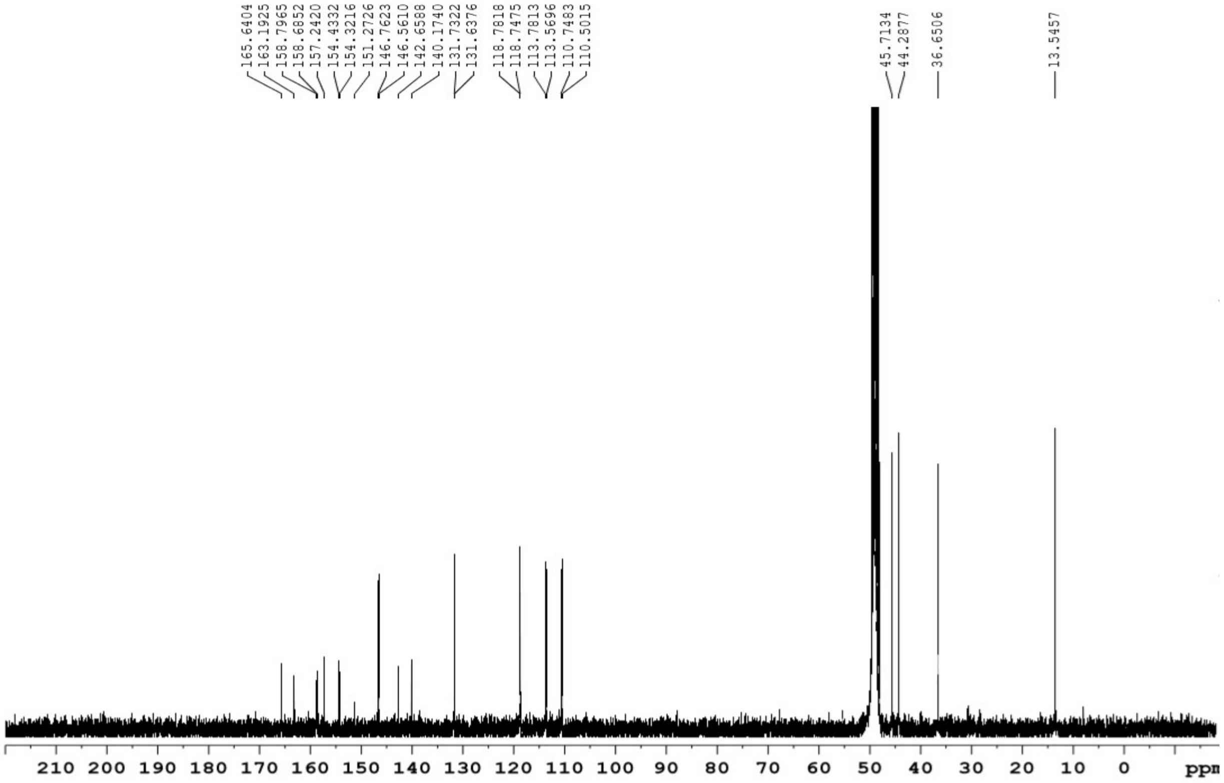

^{13}C NMR Spectrum of Compound **20a** (100 MHz)

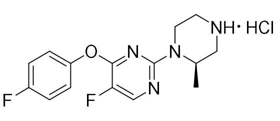

20b

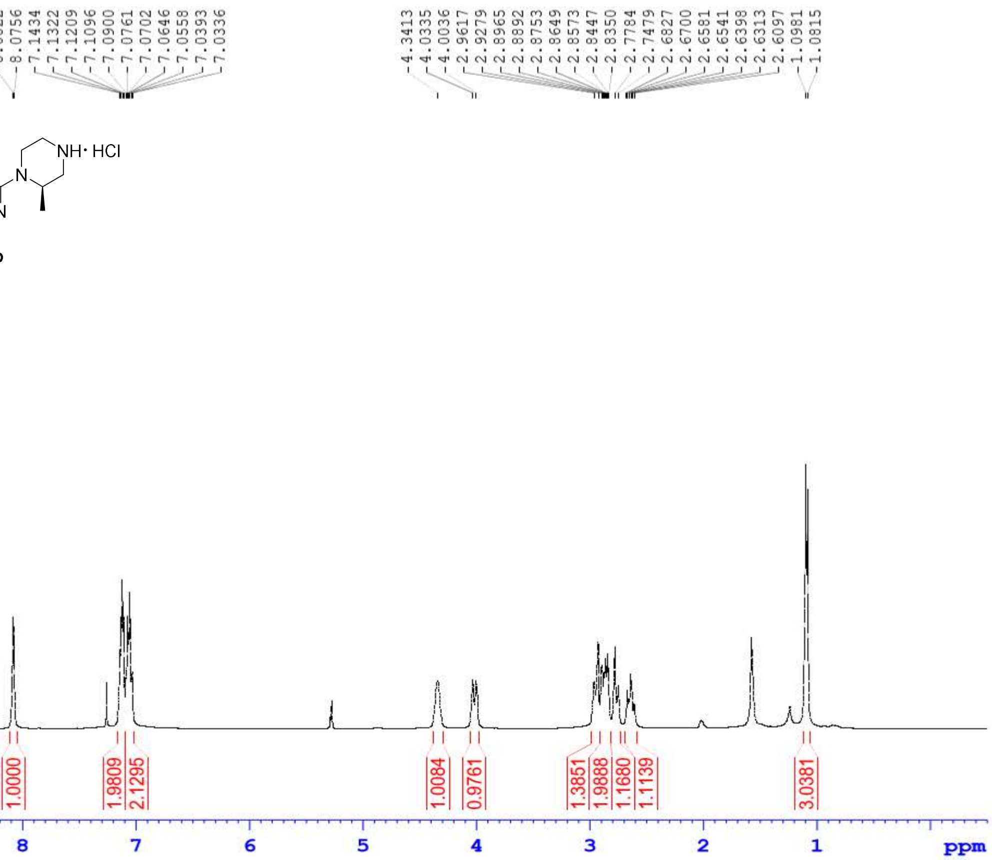

^{1}H NMR Spectrum of Compound **20b** (400 MHz)

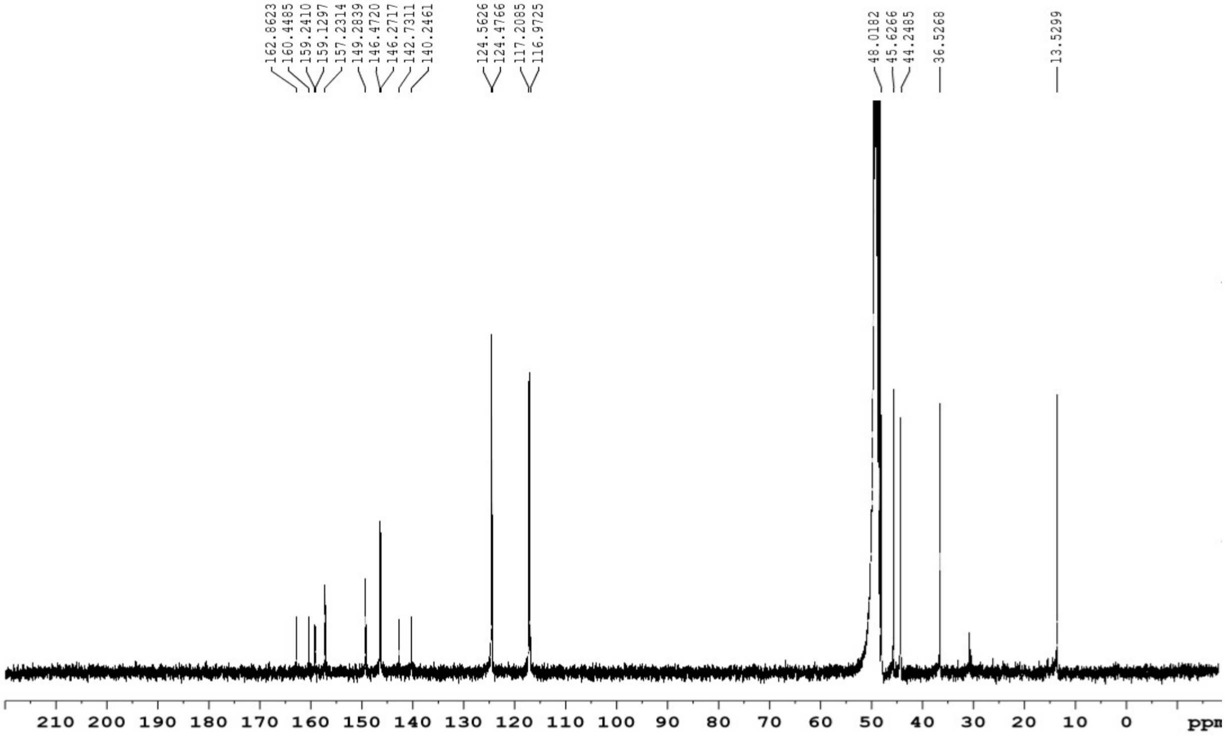

^{13}C NMR Spectrum of Compound **20b** (100 MHz)

3. Radioligands and reference compounds for binding assay

Table S1. A list of 5-HT receptor radioligands and reference compounds for binding assay.

Receptor subtype	Radioligand	Reference compound
**1A**	[^{3}H]8-OH-DPAT	Methysergide
**1B**	[^{3}H]GR125743	Ergotamine
**1D**	[^{3}H]GR125743	Ergotamine
**1E**	[^{3}H]5-HT	5-HT
**2A**	[^{3}H]Ketanserin	Chlorpromazine
**2B**	[^{3}H]LSD	5-HT
**2C**	[^{3}H]Mesulergine	Chlorpromazine
**3**	[^{3}H]LY278584	LY278584
**5A**	[^{3}H]LSD	Ergotamine
**6**	[^{3}H]LSD	Chlorpromazine
**7**	[^{3}H]LSD	Chlorpromazine
